# Ligand survey results in identification of PNP pincer complexes of iridium as long-lived and chemoselective catalysts for dehydrogenative borylation of terminal alkynes†
†Electronic supplementary information (ESI) available: Supporting information in the form of descriptions of experiments, characterization data, crystallographic information in the form of CIF files, and a video recording of hydrogen evolution from the mixture of 3-methyl-3-trimethylsiloxy-1-butyne, **17-Ir-COE** and HBpin in toluene. CCDC 1014977–1014979 and 1015119. For ESI and crystallographic data in CIF or other electronic format see DOI: 10.1039/c5sc02161h


**DOI:** 10.1039/c5sc02161h

**Published:** 2015-08-04

**Authors:** Chun-I Lee, Jessica C. DeMott, Christopher J. Pell, Alyson Christopher, Jia Zhou, Nattamai Bhuvanesh, Oleg V. Ozerov

**Affiliations:** a Department of Chemistry , Texas A&M University , College Station , TX 77842 , USA . Email: ozerov@chem.tamu.edu; b Department of Chemistry , Brandeis University , MS 015, 415 South Street , Waltham , MA 02454 , USA; c Department of Chemistry , Harbin Institute of Technology , Harbin 150001 , China

## Abstract

Following the report on the successful use of SiNN pincer complexes of iridium as catalysts for dehydrogenative borylation of terminal alkynes (DHBTA) to alkynylboronates, this work examined a wide variety of related pincer ligands in the supporting role in DHBTA. The ligand selection included both new and previously reported ligands and was developed to explore systematic changes to the SiNN framework (the 8-(2-diisopropylsilylphenyl)aminoquinoline). Surprisingly, only the diarylamido/bis(phosphine) PNP system showed any DHBTA reactivity. The specific PNP ligand (bearing two diisopropylphosphino side donors) used in the screen showed DHBTA activity inferior to SiNN. However, taking advantage of the ligand optimization opportunities presented by the PNP system *via* the changes in the substitution at phosphorus led to the discovery of a catalyst whose activity, longevity, and scope far exceeded that of the original SiNN archetype. Several Ir complexes were prepared in a model PNP system and evaluated as potential intermediates in the catalytic cycle. Among them, the (PNP)Ir diboryl complex and the borylvinylidene complex were shown to be less competent in catalysis and thus likely not part of the catalytic cycle.

## Abstract 2

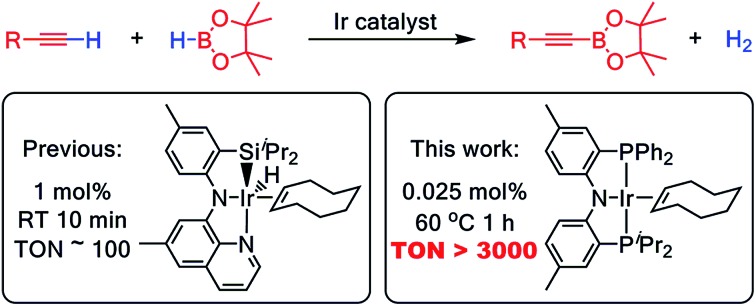
A new, highly active Ir catalyst for dehydrogenative borylation of terminal alkynes (DHBTA) has been identified.

## Introduction

**Fig. 1:**
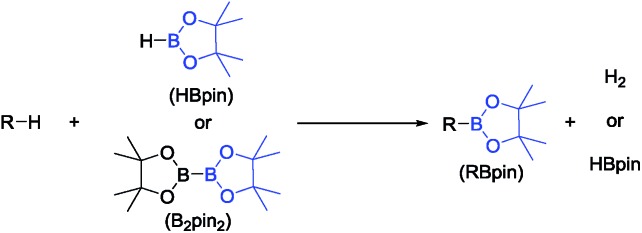
C–H borylation (dehydrogenative borylation).

Selective conversion of C–H bonds into C–B bonds (Fig. 1) has attracted broad attention over the last two decades.^1^,^2^ The resulting organoboron compounds are relatively stable, non-toxic, and can be easily transformed into C–O or C–C bonds *via* oxidation^3^–^6^ or Suzuki–Miyaura coupling.^7^–^9^ Many examples of alkane,^10^–^12^ arene,^13^,^14^ and alkene^15^,^16^ dehydrogenative borylation, as well as benzylic^17^ and allylic^18^ C–H borylation have been reported. The dehydrogenative arene borylation has been proven especially fruitful with very impressive advances by Hartwig *et al.*^19^,^20^ and Smith and Maleczka *et al.*^21^,^22^ already finding applications.^2^

The development of C–H borylation methods did not include the C(sp)–H bonds of terminal alkynes. The products of dehydrogenative borylation of terminal alkynes (DHBTA), alkynylboronates, are versatile building blocks in synthetic chemistry. Their synthetic value derives not just from the direct use in C–C_alkynyl_ coupling,^23^ but more so from pursuing the reactions of the triple bond. Cyclotrimerization,^24^ [3 + 2] cycloaddition,^25^ cyclopentenone synthesis,^26^ hydrozirconation,^27^ enyne metathesis,^28^ and others^29^–^32^ have been reported; these reactions yield more complex molecules that contain C–B bonds in positions that would be difficult to borylate by alternative means.

The classical synthesis of alkynylboronates was developed by Brown *et al.*: deprotonation of alkyne by *n*-BuLi, followed by reaction with a boric ester and quench with anhydrous acid.^33^ An Ag-catalyzed variation was reported in 2014 by Hu *et al.*^34^ Ingleson *et al.* also recently demonstrated that certain borenium cations can react with terminal alkynes to give alkynylboronates.^35^ Just as with borylation of sp^2^ and sp^3^ C–H bonds, catalysis of direct coupling of a C–H bond with a B–H bond (Fig. 1 and R = alkynyl for DHBTA) carries significant advantages (if the catalysis is efficient enough): better atom economy, as well as milder conditions allowing greater functional group compatibility. In comparison to C(sp^2^)–H and C(sp^3^)–H bonds, for the relatively acidic C(sp)–H bonds (p*K*_a_ ∼ 25) of terminal alkynes, the C–H activation itself is generally not a difficult task and C–H bond selectivity would not typically be an issue. On the other hand, in contrast to the non-olefinic C(sp^2^)–H and C(sp^3^)–H substrates, a combination of a triple C≡C bond, a B–H bond and a metal catalyst is very likely to lead to hydroboration.^36^,^37^ In addition, hydrogenation^38^ of the alkyne substrate or product with H_2_ (the by-product of DHBTA) may also be a concern.

**Fig. 2:**
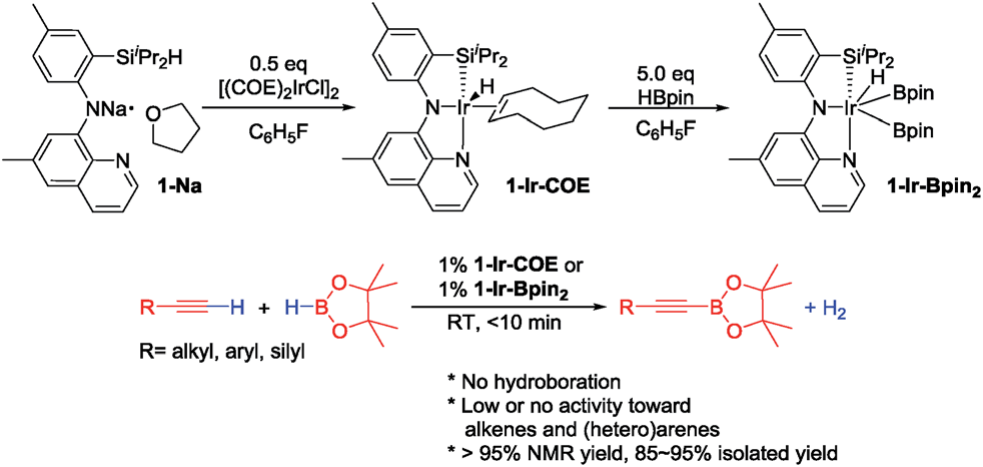
Synthesis of SiNN Ir complexes (top) and DHBTA catalyzed by SiNN Ir complexes (bottom).

In 2013, we reported the first example of catalytic DHBTA performed by Ir complexes of a SiNN pincer^39^ ligand (**1-Ir-COE** and **1-Ir-Bpin_2_**, Fig. 2).^40^ The reaction was strictly chemoselective and could be performed under very mild conditions (ambient temperature, *ca.* 10 turnovers per min) with a variety of alkyl-, aryl- and silyl- terminal alkynes in high yield. However, the catalyst longevity was limited to *ca.* 100 turnovers. Very recently, Tsuchimoto *et al.* described DHBTA catalysis by Zn(OTf)_2_/pyridine using 1,8-nathpthalenediamidoborane.^41^ The Tsuchimoto process displayed a wide scope similar to (SiNN)Ir, but operated much slower (*ca.* 1 turnover per hour) even at 100 °C. Our group also reported that (POCOP)Pd complexes are modest DHBTA catalysts for some substrates.^42^

The discovery of the prowess of the SiNN ligand in DHBTA was rather serendipitous, and we sought to explore the associated ligand space in a more systematic fashion. Here we report the exploration of a series of Ir complexes of related ligands as potential catalysts in DHBTA that has led to the discovery of a new highly active, much more long-lived catalyst with a broader scope, as well as to the insight into the role of possible intermediates in DHBTA.

## Results and discussion

### Synthesis and screening of ligands for DHBTA

**Fig. 3:**
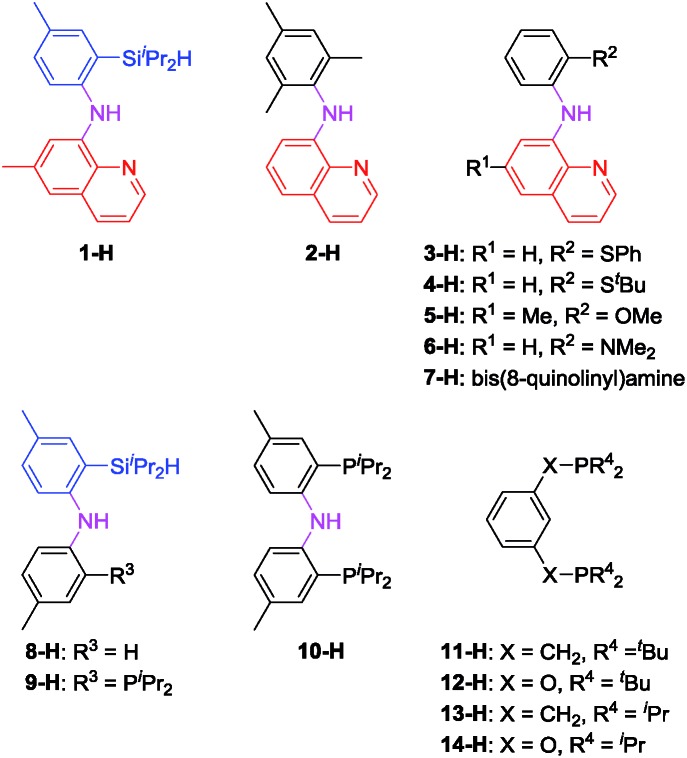
Ligands selected for DHBTA screening.

In light of the success of **1-Ir-COE** in DHBTA, we decided to examine a series of ligands that systematically explored variations of the SiNN ligand features (Fig. 3). From **2-H** to **7-H**, we preserved the central amido donor and the quinoline fragment of SiNN but removed the silane side arm (**2-H**) or replaced it with hemilabile donors (**3-H** to **7-H**). For **8-H** and **9-H**, the silane segment and the central amido donor were maintained while the quinoline moiety was eliminated (**8-H**) or substituted with a phosphine donor (**9-H**). We also included the PNP ligand (**10-H**) and the PCP/POCOP ligands (**11-H** to **14-H**) because these are commonly used pincer ligands with a rich history of C–H activation chemistry with Ir.^43^–^46^

**Scheme 1:**
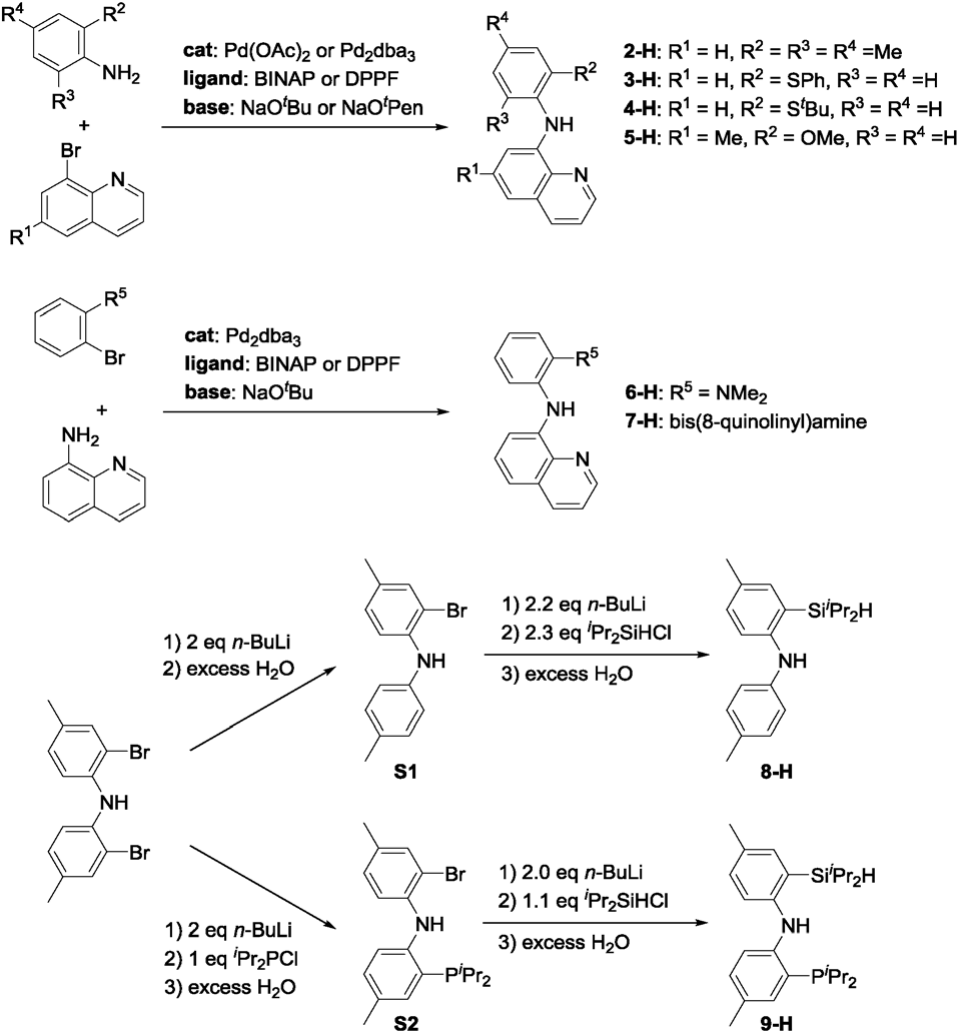
Synthesis of ligands used in screening for DHBTA.

The syntheses of ligands used in the screening of DHBTA are shown in Scheme 1. Quinoline derivatives (*i.e.***2-H**,^47^**3-H**, **4-H**, **5-H**, **6-H**,^48^**7-H**^48^) were readily synthesized *via* Buchwald–Hartwig coupling of 8-bromoquinoline with various anilines or 8-aminoquinoline with various bromoarenes. **8-H** was prepared *via* the intermediate **S1**. The synthesis of **S1** relied on the same selective dilithiation of bis(2-bromo-4-methylphenyl)amine we previously used in the synthesis of **S2**,^49^,^50^ followed by quenching with water. Pure **S1** was isolated in 86% yield by column chromatography. Treatment of **S1** with *n*-BuLi, followed by addition of ^i^Pr_2_SiHCl and workup gave **8-H** in 73% yield. The new SiNP ligand **9-H** was prepared from **S2** through a similar protocol.

**Scheme 2:**
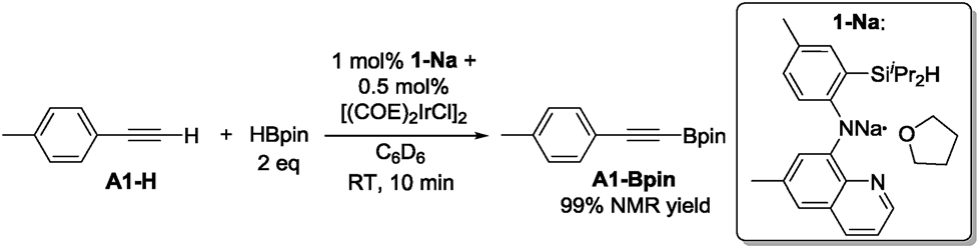
DHBTA catalyzed by **1-Ir-COE** generated *in situ* from **1-Na**.

In our original DHBTA report,^40^ we demonstrated that generation of the **1-Ir-COE** precatalyst *in situ* from **1-Na** and [(COE)_2_IrCl]_2_ (Scheme 2) produced results equivalent to those obtained using isolated **1-Ir-COE**. Therefore, we used a similar synthetic approach here for testing catalysis using the series of ligands with central amido donors (**2-H** to **10-H**). They were deprotonated with 1 equiv. of NaN(SiMe_3_)_2_*in situ*, allowed to react with 0.5 equiv. of [(COE)_2_IrCl]_2_ in C_6_D_6_ and the resultant solutions were tested for catalytic DHBTA activity. In the case of PCP/POCOP ligands **11–14**, we isolated dihydride complexes (**11-Ir-H_2_**^51^ and **12-Ir-H_2_**^52^) or alkene complexes (**13-Ir-C_2_H_4_** and **14-Ir-COE**) to be used in DHBTA testing.

**Scheme 3:**
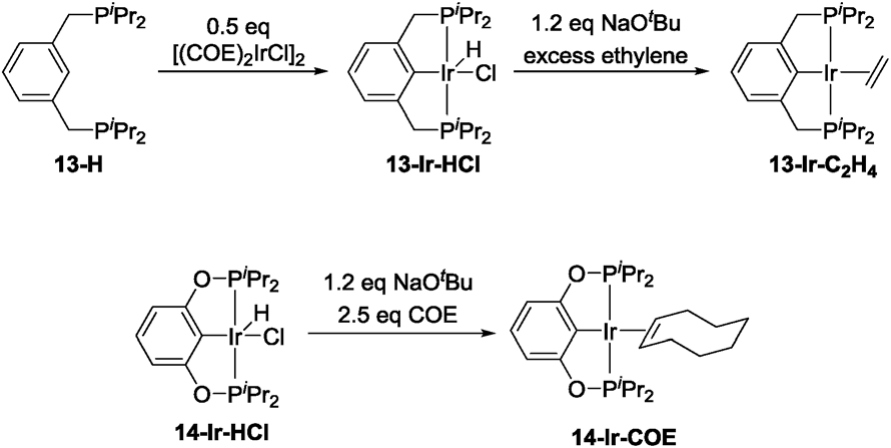
Synthesis of **13-Ir-C_2_H_4_** and **14-Ir-COE**.

The precatalyst **13-Ir-C_2_H_4_** was obtained after modification of previously reported procedures for related compounds (Scheme 3, top).^53^–^55^ Thermolysis of **13-H** with [(COE)_2_IrCl]_2_ at 80 °C overnight in toluene resulted in a dark red solution that contained *ca.* 85% of the desired product (^31^P NMR evidence). Column chromatography allowed for the collection of 99% pure **13-Ir-HCl** in 22% yield. This portion of **13-Ir-HCl** was then treated with a slight excess of NaO^*t*^Bu in toluene, degassed, and then stirred under an atmosphere of ethylene for 30 min. After filtration and removal of volatiles under vacuum, analytically pure **13-Ir-C_2_H_4_** was obtained as a dark brown solid in 66% isolated yield (based on **13-Ir-HCl**). **14-Ir-COE** was synthesized by reacting the previously reported **14-Ir-HCl**^56^,^57^ with a slight excess of NaO^*t*^Bu and COE in C_6_D_6_ (Scheme 3, bottom).

**Chart 1:**
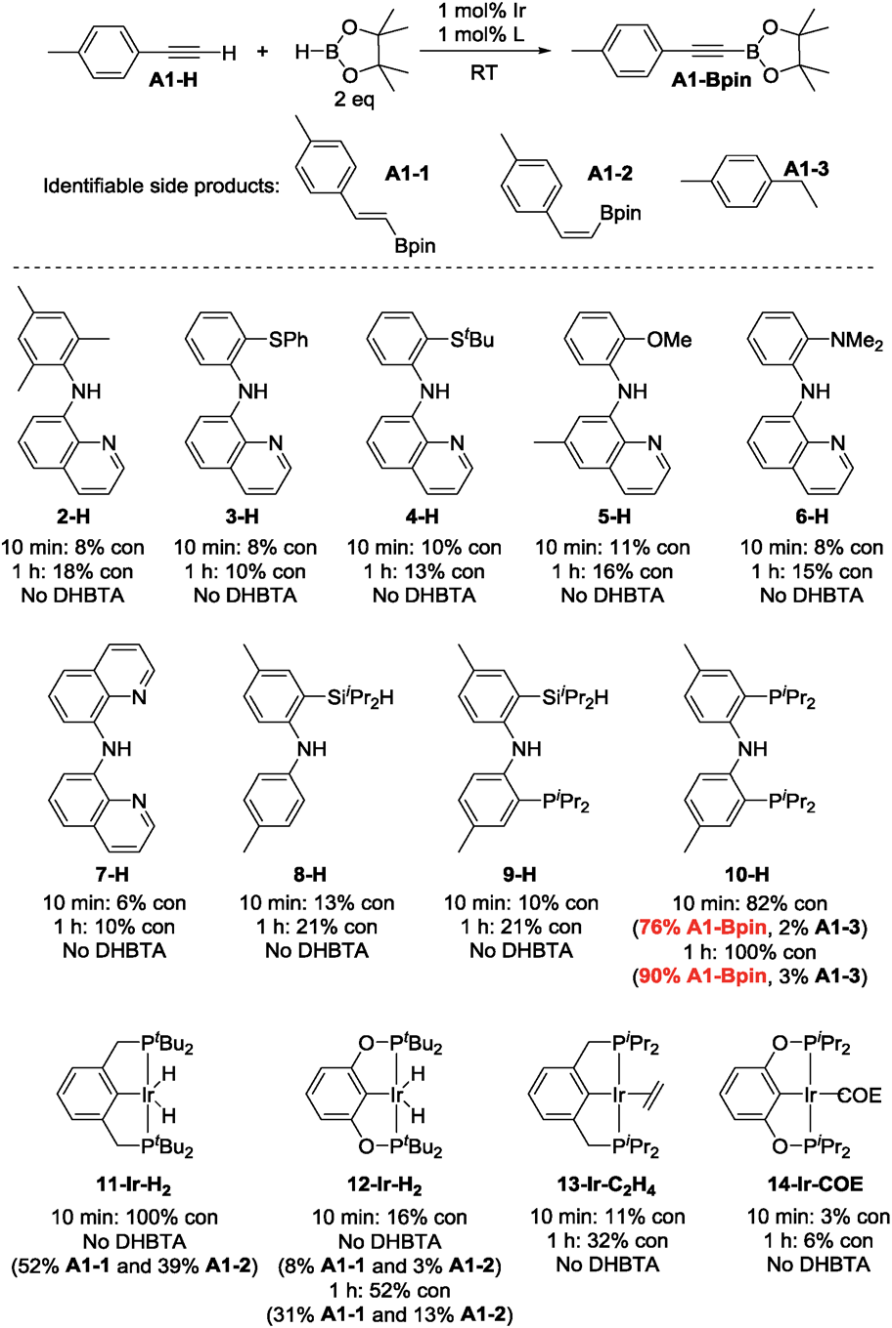
Ligand screening in DHBTA. For **2-H** to **10-H**: in the following order, the ligand (0.0010 mmol), NaN(TMS)_2_ (0.0010 mmol), [(COE)_2_IrCl]_2_ (0.00050 mmol) and HBpin (0.20 mmol) were mixed in C_6_D_6_ in a J. Young tube. 4-Ethynyltoluene (0.10 mmol) was then added in 4 portions with 1 min intervals and the mixture was allowed to stand at ambient temperature for 10 min (see ESI† for details). For **11-H** to **14-H**: the iridium complex (0.0010 mmol) and HBpin (0.20 mmol) were mixed in C_6_D_6_ in a J. Young tube. 4-ethynyltoluene (0.10 mmol) was then added in 4 portions with 1 min intervals and the mixture was allowed to stand at ambient temperature for 10 min (see ESI† for details). The numbers for “% con” refer to the conversion of **A1-H**.

4-Ethynyltoluene (**A1-H**) was selected as the alkyne for testing. We mimicked the conditions that were successful for the SiNN ligand **1**, with 1 mol% Ir loading and 2 equiv. of HBpin used at ambient temperature in C_6_D_6_ solvent. The results are summarized in Chart 1. Surprisingly, of all the ligands tested, only **10-H** showed any DHBTA reactivity. In all other cases, no evidence for the DHBTA product **A1-Bpin** was visible by ^1^H NMR spectroscopy after 1 h. In general, sluggish and nonselective hydrogenation and hydroboration was observed for **2-H** to **9-H**, and for the iso-propyl PCP/POCOP iridium complexes (**13-Ir-C_2_H_4_** and **14-Ir-COE**). For *tert*-butyl PCP/POCOP iridium complexes (**11-Ir-H_2_** and **12-Ir-H_2_**), a mixture of *trans*-alkenylboronate (**A1-1**) and *cis*-alkenylboronate (**A1-2**) were observed as major products. The use of **10-H** resulted in 76% **A1-Bpin** after 10 min and 90% (NMR evidence) after 1 h, with about 3% of 4-ethyltoluene (**A1-3**, from apparent hydrogenation of **A1-H**). We also tested one of the most active arene borylation catalyst systems ([(COD)Ir(OMe)]_2_ + 4,4′-di-*tert*-butyl-bipyridine)^58^ but no catalysis of any kind was observed after 1 h at RT.^59^ It is difficult to rationalize the results of the ligand screen other than to cautiously note that a central amido donor may be crucial and that selectivity for DHBTA is quite sensitive to the balance of steric and electronic factors.

**Chart 2:**
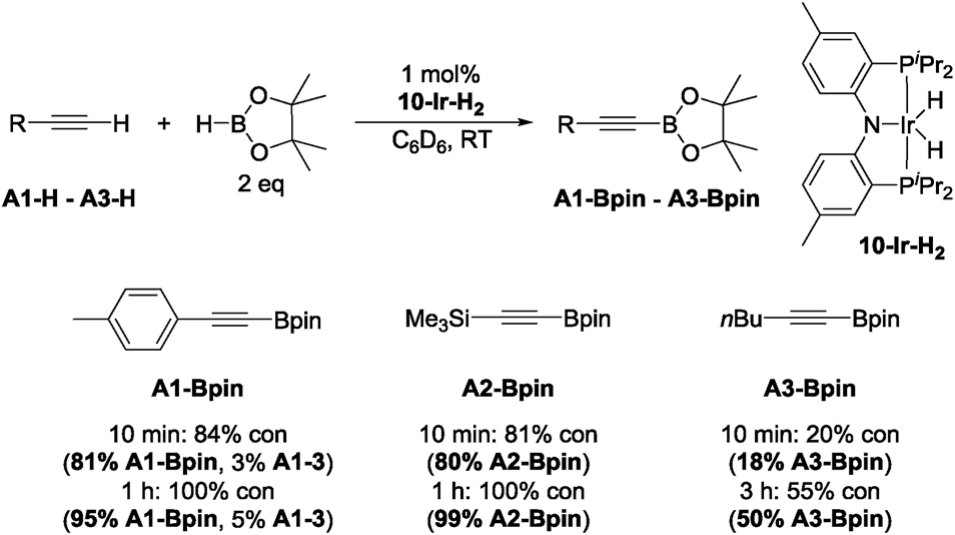
DHBTA catalyzed by **10-Ir-H_2_**.

With this lead in hand, we tested isolated **10-Ir-H_2_**^59^ as a catalyst in reactions with 4-ethynyltoluene (**A1-H**), trimethylsilylacetylene (**A2-H**), and 1-hexyne (**A3-H**) (Chart 2). The effectiveness of **10-Ir-H_2_** in the DHBTA of 4-ethynyltoluene (**A1-H**) was similar to the catalyst generated from **10-H***in situ*. DHBTA of trimethylsilylacetylene (**A2-H**) was finished in 1 h and gave an excellent yield of **A2-Bpin**. The catalytic activity of **10-Ir-H_2_** towards **A3-H** was significantly lower than towards **A1-H** and **A2-H** and only 50% yield was achieved after 3 h. A small amount of the hydrogenation product **A1-3** was observed in DHBTA of **A1-H**, but no hydrogenation products were detected in the reactions of **A2-H** and **A3-H**.

### Testing of (PNP)Ir complexes with various phosphine substituents

**Scheme 4:**
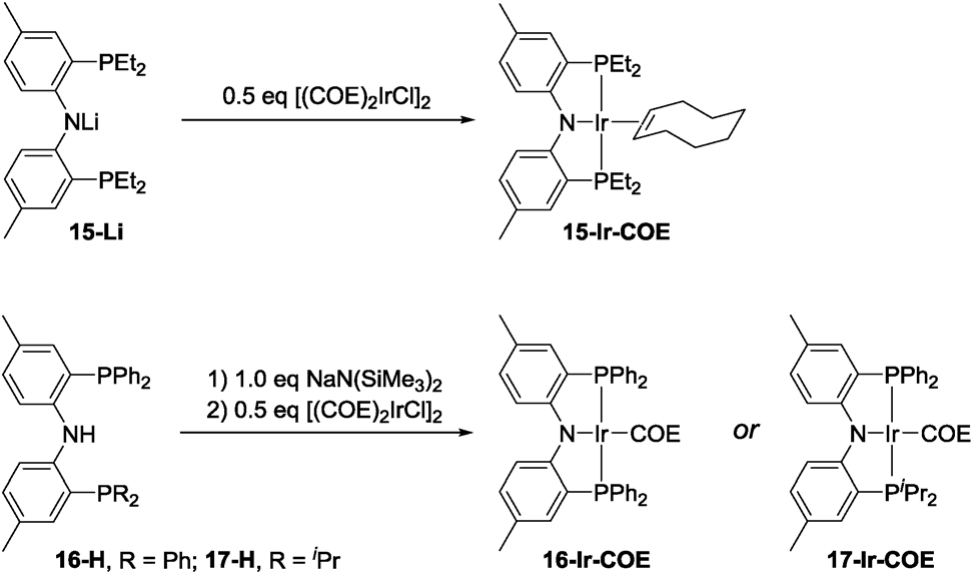
Synthesis of (PNP)Ir(COE) complexes.

The effectiveness of **10-Ir-H_2_** fell somewhat short of the SiNN-based catalysis, where >95% yield of **A1/2/3-Bpin** was obtained in <10 min and without any hydrogenation side products. Nonetheless, we were encouraged by the results because the PNP framework offers facile opportunities for optimization of the ligand *via* substituent variation. We selected previously reported PNP ligands **15-H**,^50^**16-H**,^60^ and **17-H**^50^,^61^ for further testing in DHBTA. The syntheses of the corresponding Ir-COE complexes are depicted in Scheme 4. **15-H** is an oil that is difficult to purify; however, the Li derivative (**15-Li**) could be isolated in in 56% yield as a pure solid. **15-Li** was then reacted with 0.5 equiv. of [(COE)_2_IrCl]_2_ to yield **15-Ir-COE**. **16-Ir-COE**^62^ and **17-Ir-COE** were synthesized *via* one-pot reactions by deprotonation of the neutral ligands *in situ* and treatment with [(COE)_2_IrCl]_2_.

**Fig. 4:**
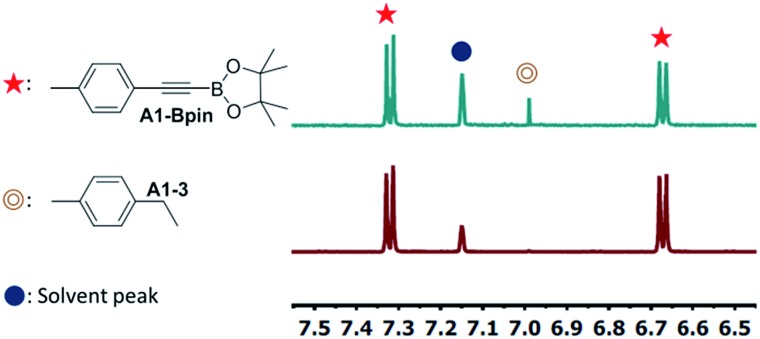
Partial ^1^H NMR spectra of DHBTA reaction mixtures catalyzed by (a) 1 mol% **15-Ir-COE** (entry 3 in Table 1) and (b) 1 mol% **1-Ir-COE** (entry 1 in Table 1).

The newly synthesized and isolated (PNP)Ir(COE) complexes (**15-Ir-COE**, **16-Ir-COE**, **17-Ir-COE**), **10-Ir-H_2_**, and the previously reported **1-Ir-COE** were all tested in DHBTA by using **A1-H** as the substrate with 2 equiv. of HBpin at ambient temperature in C_6_D_6_ solvent. The results are summarized in Table 1. At 1 mol% catalyst loading, **15-Ir-COE**, **17-Ir-COE**, **10-Ir-H_2_**, as well as **1-Ir-COE** gave excellent yields of **A1-Bpin** at ambient temperature, whereas **16-Ir-COE** did not (entry 4) and was eliminated from further consideration. At 0.25% Ir, **17-Ir-COE** showed superior reactivity to **1-Ir-COE**, **10-Ir-H_2_**, and **15-Ir-COE** by producing 92% **A1-Bpin** in 10 min. **1-Ir-COE** gave 43% yield after 1 h (entry 6) and the yield did not increase with longer reaction times, suggesting faster catalyst decomposition for the SiNN-based catalyst. **17-Ir-COE** was able to effect 100% conversion of **A-1H** and 85% yield of **A1-Bpin** (NMR evidence) even at 0.025% loading in only hours at ambient temperature. The reaction rate was higher at 60 °C (entry 12) without loss in yield. The 84% yield of **A1-Bpin** at 0.025 mol% catalyst loading (entry 12) corresponds, impressively, to 3400 turnovers. Under incomplete conversion with 0.01 mol% loading (entry 13), a turnover number of 6500 was achieved after 2 h at 60 °C. In terms of chemoselectivity, **1-Ir-COE** is superior in DHBTA of **A1-H** as it gave **A1-Bpin** as the product exclusively; 2–10% of hydrogenation product **A1-3** was observed in all reactions catalyzed by the (PNP)Ir complexes (Fig. 4). Because **15** and **17** gave faster catalysis than **10**, it is possible that a less sterically encumbered ligand is advantageous. The lower reactivity of **16** may in turn reflect sensitivity to the electronic factors.

**Table 1:** Catalytic results for DHBTA using various PNP Ir complexes and **1-Ir-COE**^*a*^

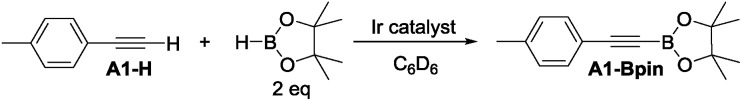						
#	Catalyst	[Ir] mol%	Time	Con (%)	Yield (%)	A1-3 (%)
1	**1-Ir-COE**	1	10 min	100	99	0
2	**10-Ir-H**_2_	1	1 h	100	95^*b*^	5
3	**15-Ir-COE**	1	10 min	100	97	2
4	**16-Ir-COE**	1	10 min	27	21	2
5	**17-Ir-COE**	1	10 min	100	97	2
6	**1-Ir-COE**	0.25	1 h	44	43^*c*^	0
7	**10-Ir-H**_2_	0.25	4 h	100	90^*d*^	6
8	**15-Ir-COE**	0.25	2 h	100	82^*e*^	5
9	**17-Ir-COE**	0.25	10 min	100	92	2
10	**17-Ir-COE**	0.05	2 h	100	85	7
11	**17-Ir-COE**	0.025	8 h	100	85^*f*^	9
12	**17-Ir-COE**	0.025	1 h^*g*^	100	84	10
13	**17-Ir-COE**	0.01	2 h^*g*^	77	65	5

^*a*^The iridium complex and HBpin (0.20 mmol) were mixed in C_6_D_6_ in a J. Young tube. **A1-H** (0.10 mmol) was then added in 4 portions with 1 min intervals and the mixture was allowed to stand at ambient temperature (see ESI for details).

^*b*^10 min: 81% yield.

^*c*^10 min: 37% yield.

^*d*^10 min: 34% yield.

^*e*^10 min: 45% yield.

^*f*^10 min: 13% yield.

^*g*^Run at 60 °C.

**Chart 3:**
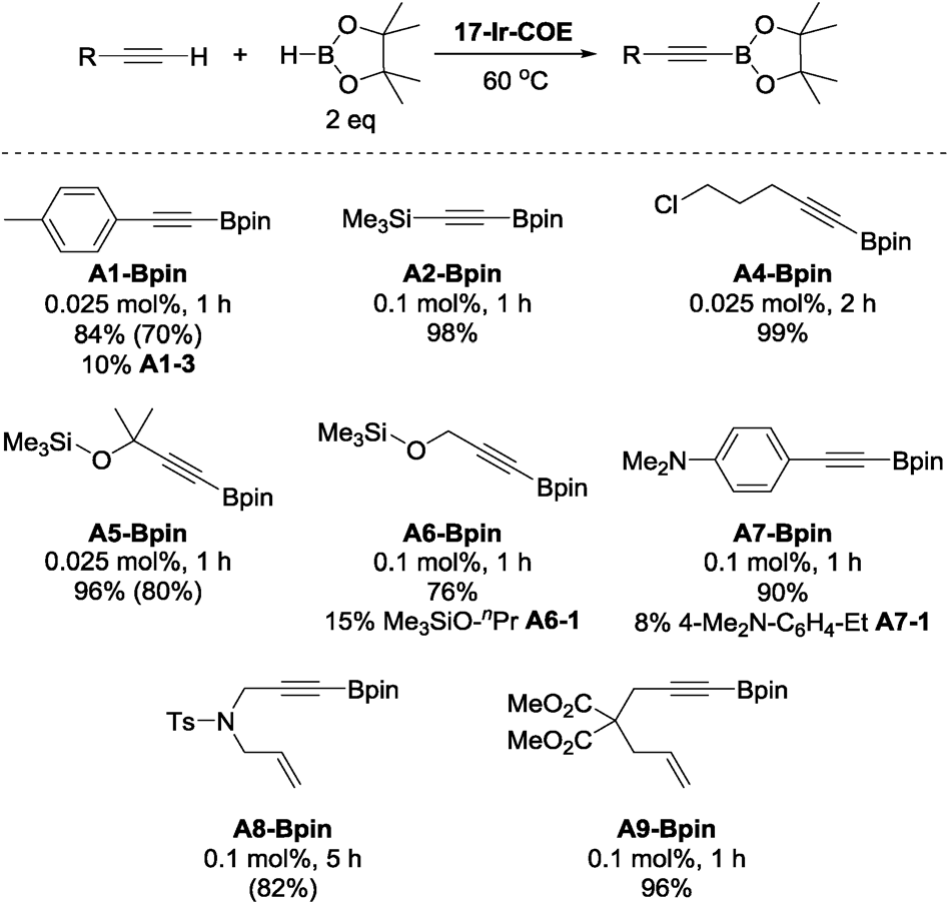
DHBTA of representative terminal alkynes catalyzed by **17-Ir-COE**. ^a^**17-Ir-COE** and HBpin (0.20 mmol) were mixed in C_6_D_6_ in a J. Young tube. Alkyne (0.10 mmol) was then added in 4 portions with 1 min intervals at RT and the mixture was heated at 60 °C (see ESI† for details). ^b^ NMR yield. ^c^ Yields in parentheses are isolated yields in preparative-scale (10 mmol alkyne) reactions that used toluene or fluorobenzene as solvent instead of C_6_D_6_.

To further explore the catalytic reactivity of **17-Ir-COE**, **A1-H**, **A2-H**, 5-chloro-1-pentyne (**A4-H**), as well as 3-methyl-3-trimethylsiloxy-1-butyne (**A5-H**), trimethylsilyl propargyl ether (**A6-H**), 4-dimethylamino-phenylacetylene (**A7-H**), *N*-tosylated allyl propargyl amine (**A8-H**), and dimethyl 2-allyl-2-(prop-2-yn-1-yl)malonate (**A9-H**) were chosen as representative substrates for aromatic, silyl, aliphatic terminal alkynes, propargyl derivatives and 1,6-enynes, respectively (Chart 3). For **A1-H**, 84% NMR yield was observed and accompanied with 10% hydrogenation product **A1-3**; similar results were obtained with **A6-H** and **A7-H**. 96–99% NMR yields were obtained for **A2-**, **A4-**, **A5**, and **A9-Bpin** with 0.025 to 0.1 mol% loading of **17-Ir-COE** as the catalyst. Borylation of **A2-H** and **A6-H** was also performed with reduced amount of HBpin (1.1 eq.) and comparable yields/side product (**A6-1** for **A6-H**) were obtained. No DHBTA products were observed for phenyl propargyl sulfide, 3-ethynylpyridine, 4-cyano-1-butyne, 3,3-diethoxy-1-propyne, and methyl propiolate with 0.1 mol% **17-Ir-COE**. **A1-Bpin**, **A5-Bpin** and **A8-Bpin** could be easily purified by recrystallization and were isolated in good yields in preparative-scale reactions. In contrast, **1-Ir-COE** requires 1% loading for high yields of **A1-**, **A2-**, **A4-**, and **A5-Bpin**, and is altogether ineffective for the synthesis of propargyl derivatives **A6-** and **A8-Bpin** (<10% yield at 1% catalyst loading). A mercury drop test^63^ was performed with 0.025 mol% **17-Ir-COE** loading and **A1-H** as substrate. No significant yield changes were observed for either **A1-Bpin** or the major side-product **A1-3** which suggested that the catalysis is homogeneous.

### Synthesis of plausible DHBTA intermediates

In order to gain new insight into the reaction mechanism, we set out to examine conceivable intermediates in DHBTA. Because of its NMR-friendly *C*_2v_-symmetric structure, and because a number of its iridium complexes are already known,^44^–^46^ we opted for ligand **10** for this study. We were particularly interested in determining the possible products arising from combining the **10-Ir** fragment with HBpin, terminal alkynes, and alkynylboronates and their catalytic competence.

**Scheme 5:**
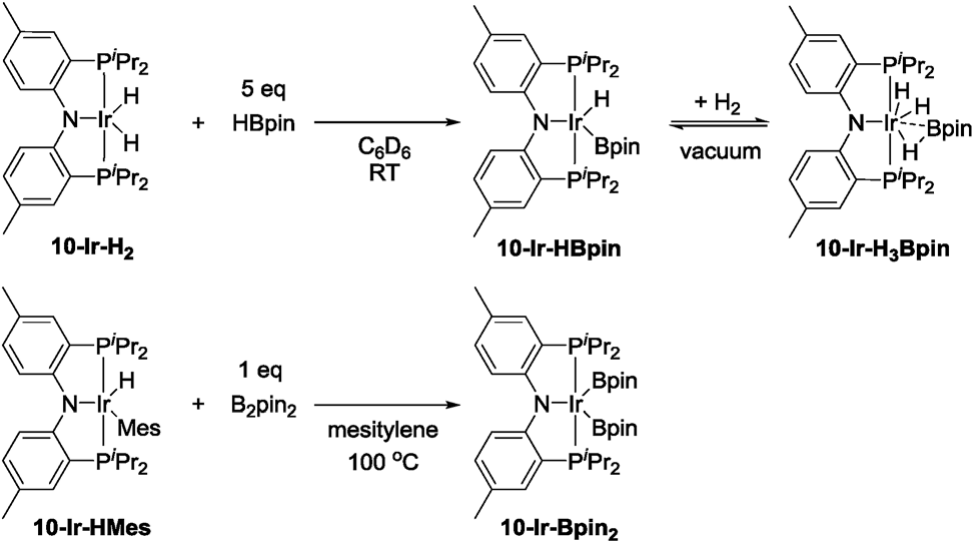
The synthesis of **10-Ir-HBpin** and its equilibrium with **10-Ir-H_3_Bpin** (top). The synthesis of **10-Ir-Bpin_2_** (bottom).

In our report on the DHBTA activity of SiNN-based catalysts, we showed that the iridium diboryl complex **1-Ir-Bpin_2_** can be synthesized by reacting **1-Ir-COE** with 5 equivalents of HBpin,^40^ and that isolated **1-Ir-Bpin_2_** exhibited the same catalytic activity as **1-Ir-COE**. Treating **10-Ir-H_2_** with 5 equivalents of HBpin, however, led to a mixture of **10-Ir-H_3_Bpin** and **10-Ir-HBpin** in equilibrium with free H_2_ (top, Scheme 5). To access **10-Ir-Bpin_2_**, we employed an alternative route of heating **10-Ir-HMes**, a good synthon for **10-Ir**,^45^ with 1 equiv. of B_2_pin_2_. This permitted isolation of **10-Ir-Bpin_2_** in 83% yield (bottom, Scheme 5).

**Fig. 5:**
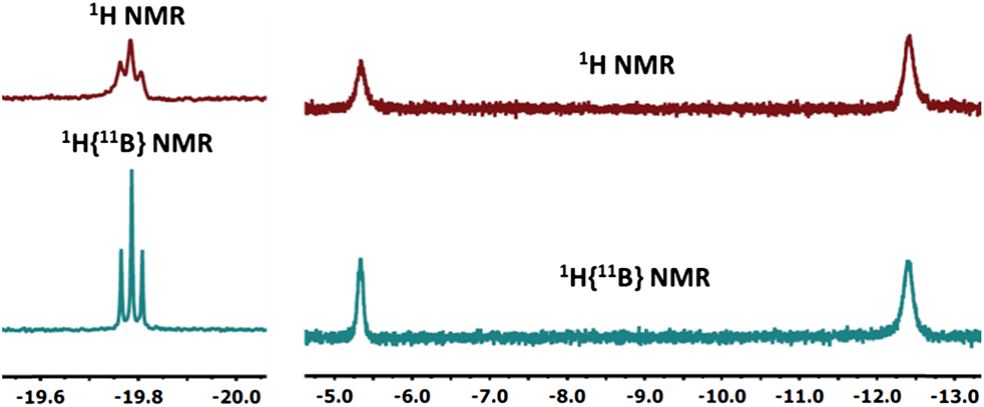
The upfield region of ^1^H and ^1^H{^11^B} NMR spectrum (400 MHz, C_6_D_6_) of **10-Ir-HBpin** (left) and **10-Ir-H_3_Bpin** (right).

**Fig. 6:**
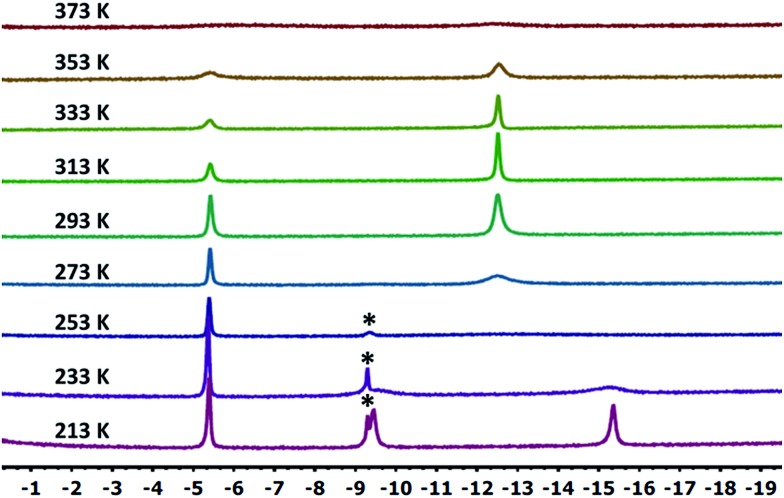
Partial (upfield region) ^1^H NMR spectrum (500 MHz, toluene-*d*_8_) of **10-Ir-H_3_Bpin** as a function of temperature. Small amount of unidentified impurity (marked with asterisks) was shown near –9.2 ppm.

**10-Ir-HBpin** exhibited an upfield signal at –19.8 ppm (t, *J*_P-H_ = 8.4 Hz, 1H) in its ^1^H NMR spectrum, and the peak sharpened upon ^11^B decoupling (Fig. 5, left) which suggested that this proton interacted with a boron atom of the boryl. **10-Ir-H_3_Bpin** displayed two broad upfield signals at –5.3 (1H, *ω*_1/2_ = 60 Hz) and –12.4 (2H, *ω*_1/2_ = 64 Hz) ppm in the ^1^H NMR spectrum at ambient temperature. The resonance at –5.3 ppm (*ω*_1/2_ = 35 Hz) sharpened upon ^11^B decoupling (Fig. 5, right), but the width of the peak at –12.4 ppm remained unchanged, indicating that only the proton associated with the resonance at –5.3 ppm displayed substantial coupling to the boron nucleus. The –12.4 ppm signal of **10-Ir-H_3_Bpin** resolved into two distinct resonances (–9.43, –15.35 ppm, Fig. 6) upon cooling to 213 K. On the basis of the ^1^H{^11^B} and VT ^1^H NMR spectroscopic data, **10-Ir-H_3_Bpin** is best described as an *exo*-σ-borane dihydride complex, similarly to **12-Ir-H_3_Bpin** in the study by Goldberg and Heinekey.^64^

Reactions of **10-Ir-H_2_** or **10-Ir-HMes** with one equivalent or excess of **A1-H** under various conditions all led to mixtures of unidentified products that have resisted our attempts at isolation and separation. The phenomenon might be related to the fact that Rh analog **10-Rh-H_2_** has been shown to be an alkyne dimerization catalyst^65^ and PCP/POCOP iridium complexes reacted with alkynes to form a variety of allene or enyne complexes.^66^,^67^

**Fig. 7:**
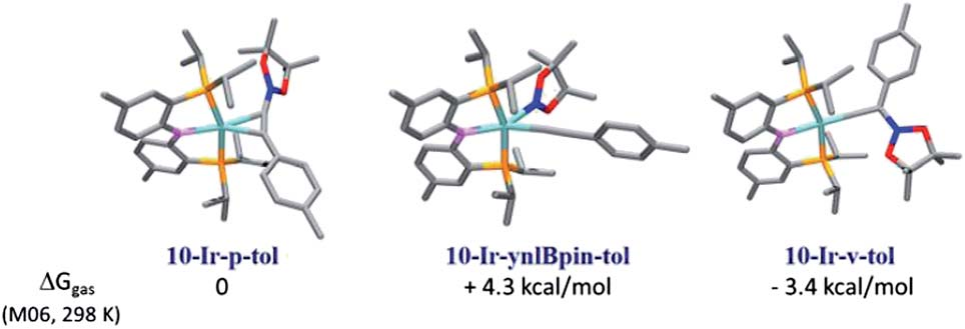
Relative free energies (DFT calculation) of three possible isomers of [**10-Ir** + **A1-Bpin**].

For the 1 : 1 combination of **10-Ir** with **A1-Bpin**, we used DFT calculations (M06/SDD/6-311G(d,p) level of theory, see details in ESI†) to evaluate the relative thermodynamic stability of the three conceivable isomeric structures: the alkynylboronate π-complex **10-Ir-p-tol**; the vinylidene complex **10-Ir-v-tol**; and the alkynyl boryl complex **10-Ir-ynlBpin-tol** (Fig. 7). **10-Ir-v-tol** was calculated to be the lowest energy isomer, with **10-Ir-p-tol** and **10-Ir-ynlBpin-tol** lying 3.4 and 7.7 kcal mol^–1^ higher in energy, respectively.

**Scheme 6:**
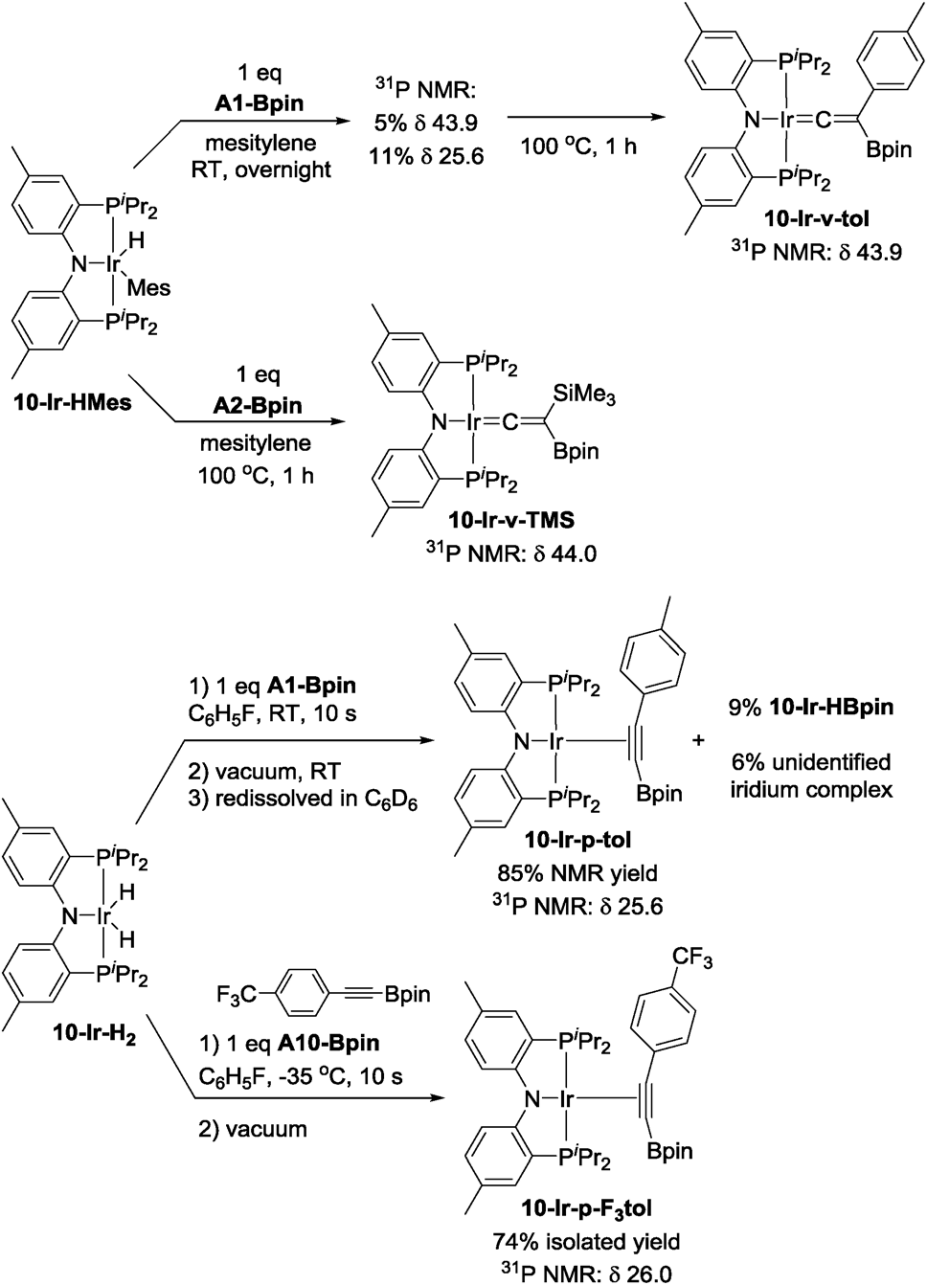
Synthesis of vinylidene complexes **10-Ir-v-tol** and **10-Ir-v-TMS**, and *p*-alkyne complexes **10-Ir-p-tol** and **10-Ir-p-F_3_tol**.

Mixing **10-Ir-HMes** with one equivalent of **A1-Bpin** at ambient temperature overnight led to two products which appeared at 43.9 (5%) and 25.6 (11%) ppm respectively in the ^31^P{^1^H} NMR spectrum (Scheme 6). Further heating the mixture at 100 °C for 1 h cleanly converted all iridium complexes to a single product that resonated at 43.9 ppm in the ^31^P NMR spectrum, which was isolated and identified as **10-Ir-v-tol**. The Me_3_Si-substituted vinylidene analog **10-Ir-v-TMS** was also characterized by NMR spectroscopy in solution by using **A2-Bpin** as the reactant (Scheme 6). The vinylidene resonances were observed at 282.8 and 269.3 ppm in the ^13^C NMR spectrum as expected for **10-Ir-v-tol** and **10-Ir-v-TMS**, respectively.^68^ These results are consistent with the DFT prediction of **10-Ir-v-tol** as the thermodynamically favored isomer.

Esteruelas and López^69^ were able to monitor the conversion of osmium boryl alkynyl complexes to vinylideneboronate esters, and we envisaged that other isomers of **10-Ir-v-tol** might be obtained if a suitable (^Me^PNP^iPr^)Ir precursor was reacted with **A1-Bpin** under milder conditions. We first attempted to mix **10-Ir-H_2_** with **A1-Bpin** with subsequent rapid removal of volatiles. The residue was redissolved in C_6_D_6_ and analyzed by ^1^H and ^31^P NMR spectroscopy. The major product was assigned as the alkynylboronate π-complex **10-Ir-p-tol** (Scheme 6), and its resonance in the ^31^P NMR spectrum appeared at 25.6 ppm, which was identical to the observed intermediate in the synthesis of **10-Ir-v-tol**. We also observed 9% of **10-Ir-HBpin** formation indicating that C_sp_–B bond cleavage is facile; the amount of **10-Ir-HBpin** increased over time. The assignment of **10-Ir-p-tol** was supported by the considerable downfield shift (8.28 ppm) of the ^1^H NMR resonances of the *ortho*-hydrogens of the *p*-tolyl group in the coordinated **A1-Bpin**. Such downfield chemical shift is characteristic of internal aromatic alkyne π-complexes.^67^,^70^,^71^ However, the isomerization from **10-Ir-p-tol** to **10-Ir-v-tol** proceeded at an appreciable rate at ambient temperature (about 50% after 15 h) and precluded the isolation of pure **10-Ir-p-tol**. We surmised that a more electron-poor alkyne should be thermodynamically less predisposed to form a vinylidene,^68^ and that the alkyne isomer may also be kinetically more long-lived. To this end, we replaced **A1-Bpin** with **A10-Bpin** as the reactant (Scheme 6) and mixed it with pre-cooled **10-Ir-H_2_** at –35 °C. We were able to isolate **10-Ir-p-F_3_tol** as a pure red-orange solid in 74% yield. To the best of our knowledge, this is the first alkynylboronate π-complex that has been isolated and characterized. ^31^P NMR spectroscopic analysis showed a singlet at 26.1 ppm, which was similar to that of **10-Ir-p-tol**. Consistent with our proposal, the conversion of **10-Ir-p-F_3_tol** to the vinylidene complex **10-Ir-v-F_3_tol** is significantly slower (about 5% after 15 h at ambient temperature) than the analogous transformation of **10-Ir-p-tol**. Similarly to **10-Ir-p-tol**, the aromatic proton signals of the 2,6-positions on **A10-Bpin** in **10-Ir-p-F_3_tol** were shifted downfield to 8.22 ppm in the ^1^H NMR spectrum. In the ^13^C NMR spectrum, the carbon signal of alkynyl–C (*C[combining low line]*≡C–B) in **10-Ir-p-F_3_tol** (*δ* 105.7) was slightly downfield of that in free **A10-Bpin**, as expected^72^ for a two-electron donor alkyne.

### Select X-ray and computational structural studies

**Fig. 8:**
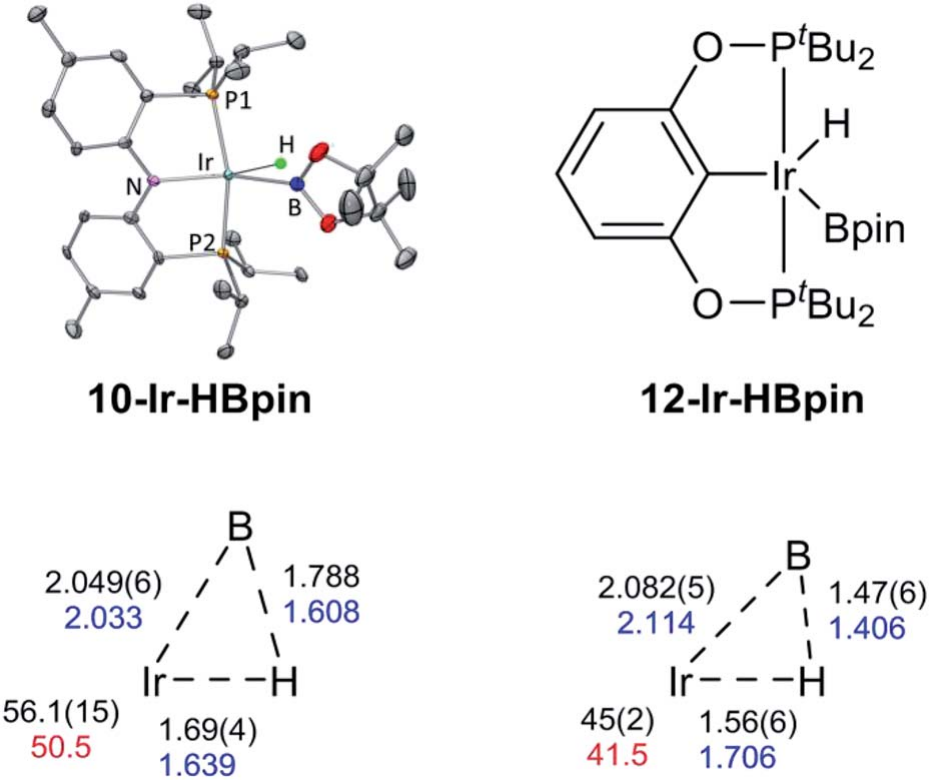
ORTEP drawing^75^ (50% probability ellipsoids) of **10-Ir-HBpin** (top left) showing selected atom labeling, and depiction of **12-Ir-HBpin** (top right). Hydrogen atoms are omitted for clarity in the ORTEP drawing, except for the hydride on the Ir atom. Metric parameters in the Ir/B/H triangles in compounds **10-Ir-HBpin** (bottom left) and **12-Ir-HBpin**^64^ (bottom right): DFT calculated distances (Å) in blue, B–Ir–H angles (°) in red, XRD-determined distances (Å) and B–Ir–H angles (°) in black.

**Fig. 9:**
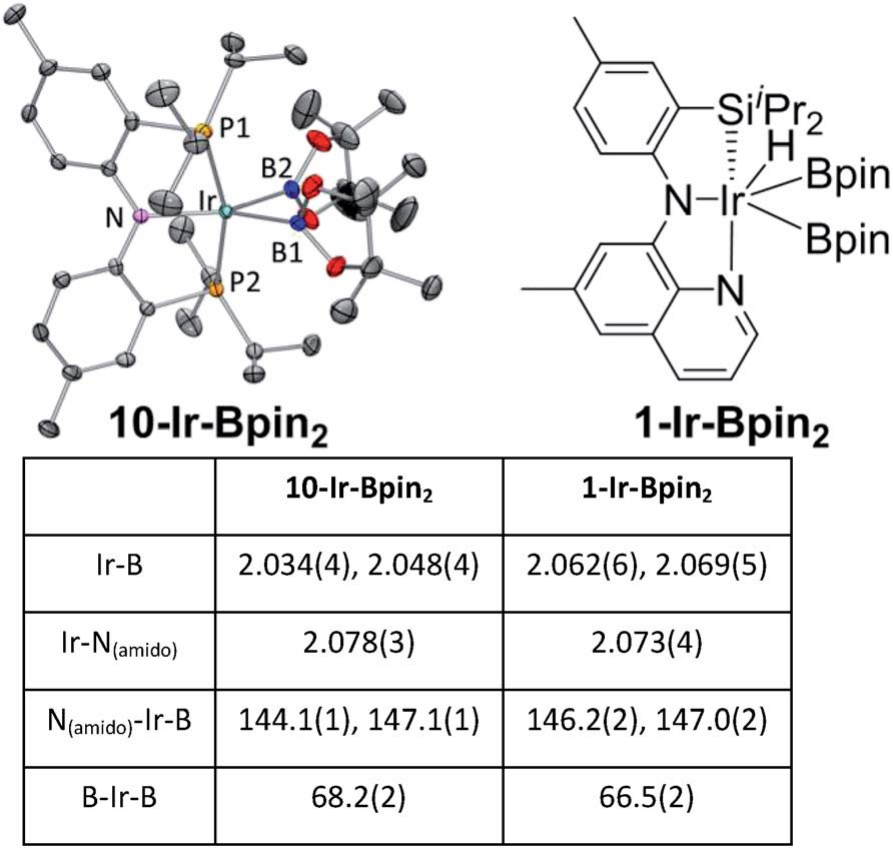
ORTEP drawing^75^ (50% probability ellipsoids) of **10-Ir-Bpin_2_** (top left) showing selected atom labeling and depiction of **1-Ir-Bpin_2_** (top right). Hydrogen atoms are omitted for clarity in the ORTEP drawing. The selected bond distances (Å) and angles (deg) for **10-Ir-Bpin_2_** and **1-Ir-Bpin_2_** are summarized in the table at bottom.

We were able to determine molecular structures of **10-Ir-HBpin**, **10-Ir-Bpin_2_**, **10-Ir-v-tol**, and **10-Ir-p-F_3_tol** in the solid state by X-ray diffractometry on corresponding single crystals. The structure of **10-Ir-HBpin** (Fig. 8, top left) can be compared against the analogous POCOP complex **12-Ir-HBpin**^64^ (Fig. 8, top right) reported by Heinekey *et al.***10-Ir-HBpin** is Y-shaped five-coordinate if viewed as a hydride boryl complex. To reinforce the X-ray studies, especially with respect to the location of the Ir–*H*, density functional theory (DFT) analysis of **10-Ir-HBpin** and **12-Ir-HBpin** in the gas phase using the M06 functional was also performed. DFT calculations show **10-Ir-HBpin** possesses shorter Ir–B and Ir–H bond distances and a B–H bond distance 0.2 Å longer than **12-Ir-HBpin** which was judged to be a σ-borane complex, suggesting greater degree of B–H bond activation in **10-Ir-HBpin**. The structure of **10-Ir-Bpin_2_** (Fig. 9, top left) can be described as Y-shaped five-coordinate where the Y is defined by N_(amido)_ and the two boryls with an acute B–Ir–B angle (68.2°). The Y-shaped geometry is expected for a five-coordinate *d*^6^ complex^73^,^74^ when the equatorial plane contains a single good π-donor (N_(amido)_) and two strong σ-donors (two boryls). The two Ir-bound Bpin fragments display essentially the same metrics, and the associated **Ir–B** distances are similar to the analogous **Ir–Bpin** distances reported in the literature (2.02–2.07 Å).^13^,^40^ In general, all parameters of bond distances and bond angles in the NIrB_2_ plane are very close to the previously reported **1-Ir-Bpin_2_** (Fig. 9, top right).^40^ The B···B distance of 2.29 Å is too long for boron–boron interaction, thus **10-Ir-Bpin_2_** should be unambiguously viewed as an Ir(iii) diboryl complex.

**Fig. 10:**
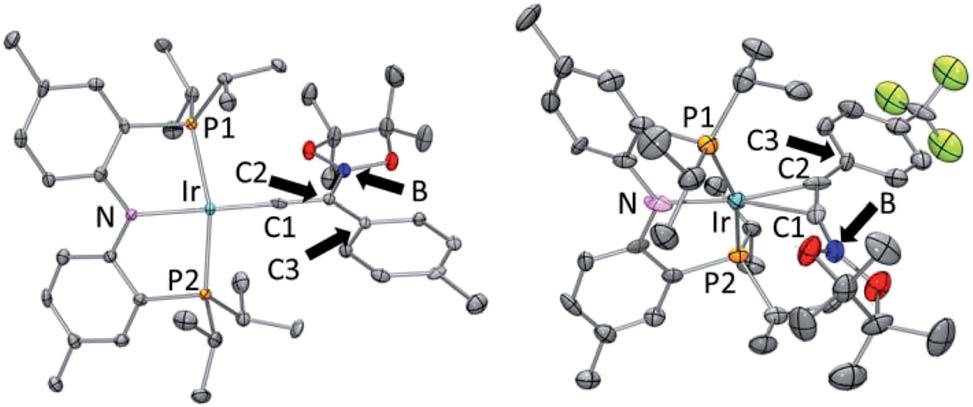
ORTEP drawings^75^ (50% probability ellipsoids) of **10-Ir-v-tol** (left) and **10-Ir-p-F_3_tol** (right) showing selected atom labeling and hydrogen atoms are omitted for clarity. For **10-Ir-v-tol**, one of two molecules in the asymmetric unit is shown, and a non-coordinated fluorobenzene molecule is omitted for **10-Ir-p-F_3_tol**. Selected bond distances (Å) and angles (deg) for **10-Ir-v-tol**: Ir–C1, 1.807(4); C1–C2, 1.334(5); P1–Ir–P2, 164.84(3); C2–C1–Ir, 178.2(3); C1–C2–C3, 120.1(3); C1–C2–B 113.1(3); C3–C2–B, 126.8(3). Selected bond distances (Å) and angles (deg) for **10-Ir-p-F_3_tol**: Ir–C1, 2.165(11); Ir–C2, 2.101(12); C1–C2, 1.301(15); P2–Ir–P1, 163.81(10); C1–C2–C3, 147.5(11); B–C1–C2, 161.9(11).

The coordination environment about Ir in the structures of **10-Ir-v-tol** and **10-Ir-p-F_3_tol** (Fig. 10) can be described as distorted square planar, with the greatest deviation corresponding to the P–Ir–P angles constrained by the pincer ligand. The C2–C1–Ir bond angle (178.2(3)°) in **10-Ir-v-tol** is very close to 180° which is typical for a vinylidene complex.^68^ The Ir–C1 and C1–C2 bond lengths of 1.807(4) and 1.334(5) Å, respectively, are similar to the analogous distances in the [(Ph_2_PCH_2_SiMe_2_)_2_N]Ir=C=CH_2_ vinylidene complex reported by Fryzuk.^76^ In the structure of **10-Ir-p-F_3_tol**, **A10-Bpin** is bound to iridium in an η^2^ fashion (Ir–C1: 2.165(11) Å, Ir–C2: 2.101(12) Å) and the Ir–C distances are within the range of other square planar Ir(i) alkyne complexes.^67^,^77^,^78^ Both the elongation of C≡C bond (1.304 Å) and the bending of C≡C–C_ipso_ (147.5(11)°) and C≡C–B (161.9(11)°) away from 180° indicate back-donation from the iridium center to the π* orbitals of C≡C bond.^72^,^79^

### Stoichiometric reactions of (^Me^PNP^iPr^)Ir complexes

**Scheme 7:**
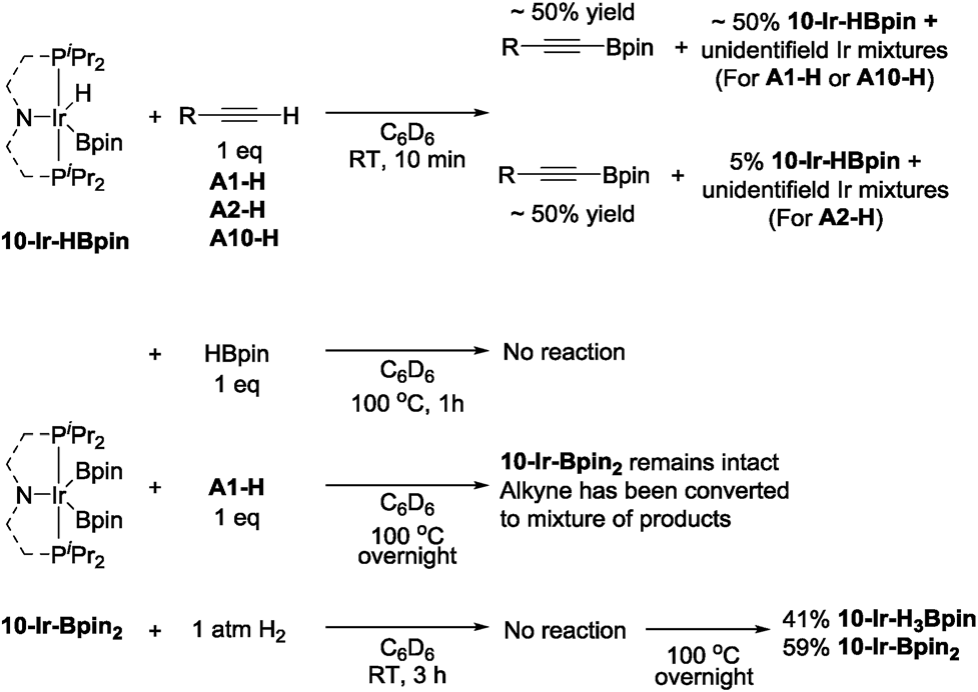
Boryl transfer from **10-Ir-HBpin** to terminal alkynes (top) and reactivity of **10-Ir-Bpin_2_**.

To examine the possible roles that four new isolated (^Me^PNP^iPr^)Ir complexes played in DHBTA, these compounds were examined in reactions with the three components in DHBTA: a terminal alkyne (substrate), HBpin (substrate), and H_2_ (by-product). **10-Ir-HBpin** was reacted with three different terminal alkynes to study the boryl transfer ability: **A1-H**, **A2-H** and **A10-H** (Scheme 7, top). After 10 min at ambient temperature, approximately 50% yield of the corresponding alkynylboronate was observed in the ^1^H NMR spectrum for each of the three substrates. The amount of alkynylboronate did not increase with longer reaction times; however, multiple side reactions including hydrogenation occurred. By ^31^P NMR spectroscopic analysis, different degrees of unreacted **10-Ir-HBpin** were observed along with multiple phosphorus-containing species formed, but they could not be assigned at this stage. Surprisingly, **10-Ir-Bpin_2_** was inert to all three major components in DHBTA: HBpin, terminal alkyne and H_2_ (Scheme 7, bottom) in stoichiometric reactions at ambient temperature. ^31^P NMR spectroscopic analysis showed **10-Ir-Bpin_2_** was the only observable phosphorus-containing compound in each reaction mixture. Even at 100 °C, **10-Ir-Bpin_2_** remained ostensibly intact in reactions with **A1-H** and HBpin. Only heating of **10-Ir-Bpin_2_** under 1 atm H_2_ at 100 °C overnight led to 41% **10-Ir-H_3_Bpin** formation.

**Scheme 8:**
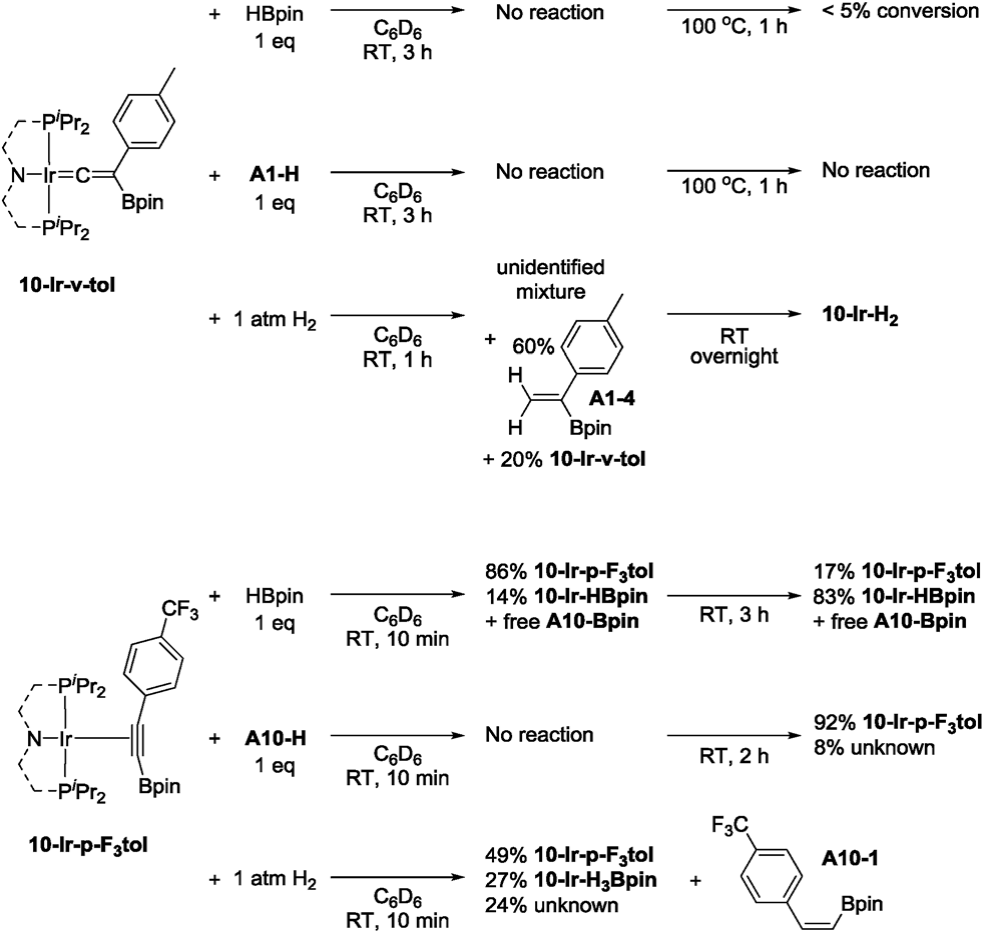
Reactivity of **10-Ir-v-tol** (top) and **10-Ir-p-F_3_tol** (bottom).

**10-Ir-v-tol** was stable toward both HBpin and **A1-H** at ambient temperature (Scheme 8, top). On the other hand, treating **10-Ir-v-tol** with H_2_ quickly resulted in 80% conversion and the formation of 60% *gem*-alkenylboronate (**A1-4**) and unidentified iridium compounds in 1 h at ambient temperature. After overnight, **10-Ir-H_2_** was the only observable species by ^31^P NMR spectroscopic analysis. Lack of observation of **A1-4** in catalytic reaction mixtures suggested that **10-Ir-v-tol** is not present in significant concentrations during catalysis. Treating **10-Ir-p-F_3_tol** with 1 equivalent of HBpin at ambient temperature cleanly led to 83% **10-Ir-HBpin** formation after 3 h (Scheme 8, bottom); meanwhile, equal amount of free **A10-Bpin** was observed in the ^1^H NMR spectrum. The reaction between **10-Ir-p-F_3_tol** and **A10-H** was relatively sluggish with no noticeable change after 10 min, and only resulted in 8% conversion after 2 h at ambient temperature based on analysis by ^31^P NMR spectroscopy. Exposing **10-Ir-p-F_3_tol** to 1 atm H_2_ quickly yielded 27% **10-Ir-H_3_Bpin** and 24% unknown iridium species in 10 min, and the formation of **10-Ir-H_3_Bpin** proved the C_sp_–B bond cleavage is facile. *cis*-Alkenylboronate (**A10-1**) was also observed.

### Competence of isolated compounds in catalytic DHBTA

**Chart 4:**
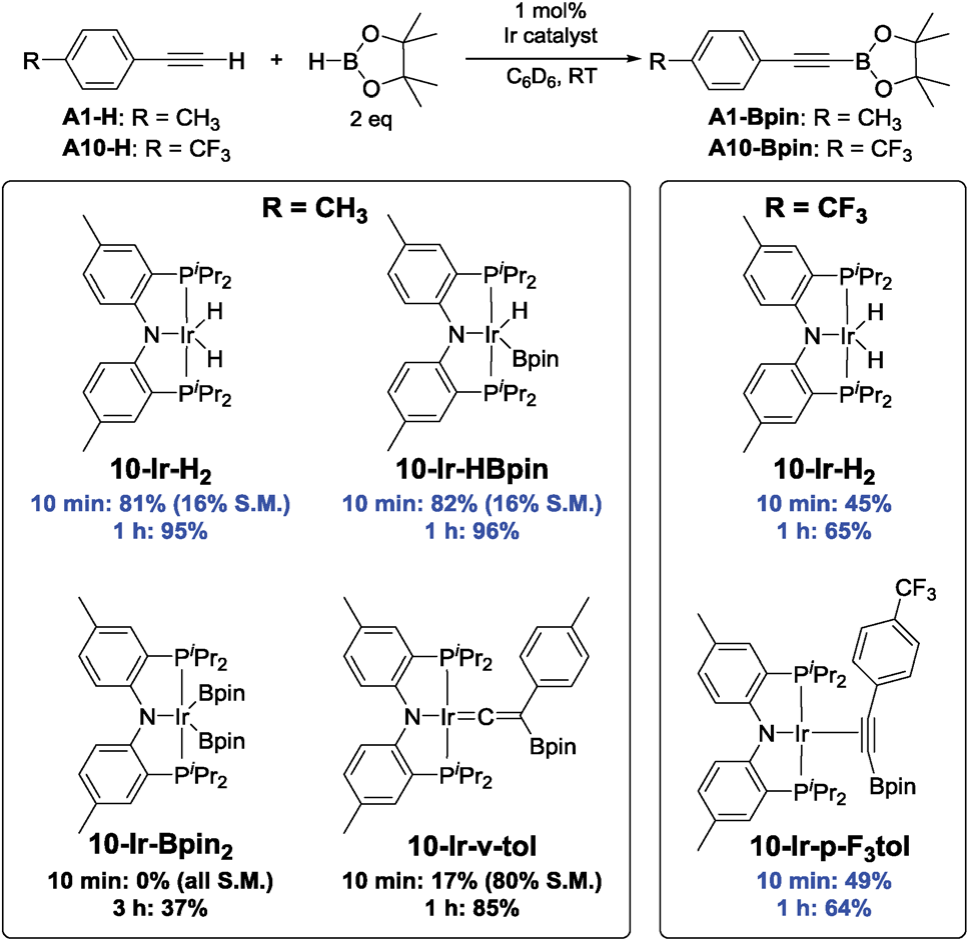
Catalytic results for DHBTA using various **10-Ir** complexes.

Chart 4 summarizes the results of catalytic DHBTA experiments that utilized various isolated (^Me^PNP^iPr^)Ir compounds as pre-catalysts. The catalytic reactions were carried out in the fashion consistent with our other studies – the Ir compound was treated with 200 equiv. of HBpin, followed by 100 equiv. of the terminal alkyne (*i.e.*, 1 mol% Ir). When **10-Ir-H_2_** was treated with excess HBpin, a yellow mixture of **10-Ir-HBpin** and **10-Ir-H_3_Bpin** immediately formed before the addition of alkyne. Not surprisingly, essentially identical yields of **A1-Bpin** and hydrogenation side-product **A1-3** was observed when using **10-Ir-HBpin** and **10-Ir-H_2_** as pre-catalysts. The use of **10-Ir-v-tol** did lead to the formation of **A1-Bpin**, but in a significantly smaller yield than with **10-Ir-HBpin** and **10-Ir-H_2_**. In contrast, **10-Ir-Bpin_2_** showed no DHBTA at all after the first 10 min and only gave 37% **A1-Bpin** after 3 h. The inertness of **10-Ir-Bpin_2_** in the DHBTA correlated with its lack of reactivity in the stoichiometric reactions described above. In the two reactions with **A10-H**, **10-Ir-H_2_** and **10-Ir-p-F_3_tol** led to the same yield of **A10-Bpin** and the hydrogenation side-product **A10-1** (4-CF_3_-C_6_H_4_-C_2_H_5_) at the 10 min and the 1 h mark.

On the basis of the stoichiometric and catalytic experiments the diboryl complexes analogous to **10-Ir-Bpin_2_** and the vinylidene complexes analogous to **10-Ir-v-tol** can be firmly ruled out as intermediates in the DHBTA catalysis by (PNP)Ir complexes. Interestingly, this raises the question of whether the previously reported (SiNN)Ir catalysis actually requires **1-Ir-Bpin_2_** as an intermediate or if it is merely an off-cycle precursor that can access the catalytic cycle rapidly enough.

## Conclusions

Building on a recent report of successful dehydrogenative borylation of terminal alkynes (DHBTA) with a pincer iridium catalyst,^40^ we examined a series of ligands structurally related to the successful SiNN ligand. Although most of the tested ligands failed to produce DHBTA products, we discovered that various PNP pincer ligands do result in active iridium DHBTA catalysts. Using the unsymmetric PNP-supported iridium complex **17-Ir-COE**, useful yields were obtained with 0.025% loading of catalyst, corresponding to thousands of turnovers. Good to excellent yields were obtained under mild conditions for aryl-, silyl-, and alkyl-substituted terminal alkynes, even propargyl derivatives and 1,6-enynes. Unlike the strict chemoselectivity in the (SiNN)Ir system, <10% hydrogenation products were observed as the main side-products in all the (PNP)Ir systems with arylacetylene substrates. This has not precluded isolation of alkynylboronate products in 70–82% yields on preparative scale.

Several iridium complexes of the symmetric PNP ligand **10** were synthesized and examined as potential intermediates in the catalytic cycle *via* testing in stoichiometric and catalytic reactions. The vinylidene (**10-Ir-v-tol**) and diboryl (**10-Ir-Bpin_2_**) complexes reacted too slowly with either terminal alkynes or HBpin under the conditions of catalysis, which ruled them out of the catalytic cycle of the **10-Ir** system. The inactivity of **10-Ir-Bpin_2_** is in contrast to the analogous **1-Ir-Bpin_2_**^40^ which suggests that a diboryl intermediate is not essential for successful DHBTA. On the other hand, the hydride boryl complex (**10-Ir-HBpin**) and the alkynylboronate π-complex (**10-Ir-p-F_3_tol**) showed nearly identical performance to **10-Ir-H_2_** indicating that they either are intermediates in the catalytic cycle or are connected to such *via* a low-barrier pathway. Although the full mechanistic picture remains uncertain, the presently reported results strongly suggest that a pincer ligand containing an amido donor is key to an active DHBTA catalyst with iridium. An intriguing possibility is that this is related to the facile migration of boryl from the metal to the amido nitrogen we recently discovered for **1-Ir-Bpin_2_** and **1-Rh-Bpin_2_**.^80^

## Supplementary Material

Supplementary information

Supplementary information

Supplementary movie

Crystal structure data
