# Correction: Ligand survey results in identification of PNP pincer complexes of iridium as long-lived and chemoselective catalysts for dehydrogenative borylation of terminal alkynes

**DOI:** 10.1039/c5sc90058a

**Published:** 2015-09-28

**Authors:** Chun-I Lee, Jessica C. DeMott, Christopher J. Pell, Alyson Christopher, Jia Zhou, Nattamai Bhuvanesh, Oleg V. Ozerov

**Affiliations:** a Department of Chemistry , Texas A&M University , College Station , TX 77842 , USA . Email: ozerov@chem.tamu.edu; b Department of Chemistry , Brandeis University , MS 015, 415 South Street , Waltham , MA 02454 , USA; c Department of Chemistry , Harbin Institute of Technology , Harbin 150001 , China

## Abstract

Correction for ‘Ligand survey results in identification of PNP pincer complexes of iridium as long-lived and chemoselective catalysts for dehydrogenative borylation of terminal alkynes’ by Chun-I Lee *et al.*, *Chem. Sci.*, 2015, ; DOI: 10.1039/c5sc02161h.



## 

The number of the US National Science Foundation grant listed in the manuscript was incorrect. The correct grant number should be CHE-1300299.

The Royal Society of Chemistry apologises for these errors and any consequent inconvenience to authors and readers.

