# Supplementary material for: Ligand survey results in identification of PNP pincer complexes of iridium as long-lived and chemoselective catalysts for dehydrogenative borylation of terminal alkynes
Source: Chem Sci. 2015 Aug 4;6(11):6572–82. doi: 10.1039/c5sc02161h (PMC6054099; doi:10.1039/c5sc02161h)
Supplement: Supplementary file 1 [file SC-006-C5SC02161H-s001.pdf]

Electronic Supplementary Material (ESI) for Chemical Science.
This journal is © The Royal Society of Chemistry 2015

Electronic Supplementary Information

Ligand Survey Results in Identification of PNP Pincer Complexes of Iridium as Long-lived and Chemoselective Catalysts for Dehydrogenative Borylation of Terminal Alkynes

*Chun-I Lee[1], Jessica C. DeMott[1], Christopher J. Pell[1], Alyson Christopher[2], Jia Zhou[3], Nattamai Bhuvanesh[1], and Oleg V. Ozerov[1]**

[1] Department of Chemistry, Texas A&M University, 3255 TAMU, College Station, TX 77842.

[2] Department of Chemistry, Brandeis University, MS 015, 415 South Street, Waltham, MA 02454

[3] Department of Chemistry, Harbin Institute of Technology, Harbin 150001, China

ozerov@chem.tamu.edu

Table of Contents.

I. General Considerations. S3

II. Computational Details. S5

III. X-ray Structural Determination Details. S6

IV. Synthesis and Screening of Potential Catalysts for DHBTA. S11

A. Synthesis of Ligands S11

B. Ligand Screening of DHBTA S19

V. Synthesis of (PNP)Ir Complexes & Examination of Their Performance in DHBTA. S23

VI. Synthesis of Proposed Intermediates in DHBTA. S33

VII. Stoichiometric Reactions of 10-Ir Complexes S42

VIII. NMR Spectra. S47

IX. SI References. S72

I. General Considerations.

Unless specified otherwise, all manipulations were performed under an Ar atmosphere using standard Schlenk line or glovebox techniques. Toluene, THF, Et_2O, pentane, C_6D_6 were dried over NaK/Ph_2CO/18-crown-6, distilled or vacuum transferred and stored over molecular sieves in an Ar-filled glovebox. Mesitylene, fluorobenzene, CH_2Cl_2, CD_2Cl_2 and $CDCl_3$ were dried with and then distilled from CaH_2 and stored over molecular sieves in an Ar-filled glove box. **1-Ir-COE**,[1] **2-H**,[2] 2-*tert*-butylmercaptoaniline,[3] 8-bromo-6-methylquinoline,[1] **6-H**,[4] **7-H**,[4] **S2**,[5] bis(2-bromo-4-methylphenyl)amine,[6] **10-Ir-H$_2$**,[7] **10-Ir-HMes**,[7] **11-Ir-H$_2$**,[8] **12-Ir-H$_2$**,[9] **13-H**,[10] **14-Ir-HCl**,[11,12] **16-H**,[13] **17-H**,[13] **A1-Bpin**,[1] **A2-Bpin**,[1] **A8-H** ,[14] **A9-H**[15] were prepared according to published procedures. $(Me_3Si)_2O$ and alkynes were deoxygenated by three freeze-pump-thaw cycles prior to use. All other chemicals were used as received from commercial vendors. NMR spectra were recorded on a Varian Inova 300, Mercury 300 (^{1}H NMR, 299.952 MHz; ^{13}C NMR, 75.421 MHz; ^{31}P NMR, 121.42 MHz), Varian Inova 400 (^{1}H NMR, 399.535 MHz; ^{11}B NMR, 128.185 MHz; ^{13}C NMR, 100.465 MHz; ^{29}Si NMR, 79.366 MHz) and NMRS 500 (^{1}H NMR, 499.703 MHz; ^{19}F NMR, 469.854 MHz; ^{13}C NMR, 125.697 MHz; ^{31}P NMR, 202.283 MHz) spectrometer. For ^{1}H and ^{13}C NMR spectra, the residual solvent peak was used as an internal reference (^{1}H NMR: δ 7.15 for C_6D_6, 5.32 for CD_2Cl_2, 7.24 for $CDCl_3$; ^{13}C NMR: δ 128.06 for C_6D_6, 53.84 for CD_2Cl_2, 77.16 for $CDCl_3$). For ^{29}Si NMR, spectra were referenced externally to $\delta = 0$ ppm by using Me_4Si. For ^{11}B NMR, spectra were referenced externally to $\delta = 0$ ppm by using $BF_3 \cdot Et_2O$. For ^{19}F NMR, spectra were referenced externally to $\delta = -78.5$ ppm by using CF_3COOH. For ^{31}P NMR, spectra were referenced externally to $\delta = 0$ ppm by using 85% H_3PO_4. Mass spectrometric analyses were carried out by the Texas A&M University Laboratory for

Biological Mass Spectrometry (LBMS). Elemental analyses were performed by CALI Labs, Inc. (Parsippany, NJ).

Note: In ^{13}C NMR spectra of alkynylboronates and vinylidenes, quaternary carbon atoms attached to boron were usually not observed due to low intensity.

II. Computational Details.

All computations were carried out with the Gaussian09 program.[16] All of the geometries were fully optimized by M06[17] functional. The Stuttgart basis set and the associated effective core potential (ECP) was used for Ir atom, and an all-electron 6-311G(d,p) basis set was used for the other atoms. The harmonic vibrational frequency calculations were performed to ensure that a minimum was obtained. The energies reported here are Gibbs free energies in the gas phase at 298.15 K and 1 atm unless noted otherwise.

III. X-ray Structural Determination Details.

X-Ray data collection, solution, and refinement for 10-Ir-HBpin (CCDC 1015119). A Leica MZ 75 microscope was used to identify a suitable yellow block with very well defined faces with dimensions (max, intermediate, and min) 0.65 mm x 0.55 mm x 0.30 mm from a representative sample of crystals of the same habit. The crystal mounted on a nylon loop was then placed in a cold nitrogen stream (Oxford) maintained at 110 K. A BRUKER GADDS X-ray (three-circle) diffractometer was employed for crystal screening, unit cell determination, and data collection. The goniometer was controlled using the FRAMBO software, v.4.1.05.[18] The sample was optically centered with the aid of a video camera such that no translations were observed as the crystal was rotated through all positions. The detector was set at 5.0 cm from the crystal sample. The X-ray radiation employed was generated from a Cu sealed X-ray tube (K_α = 1.5418 Å with a potential of 40 kV and a current of 40 mA) fitted with a graphite monochromator in the parallel mode (175 mm collimator with 0.5 mm pinholes). 180 data frames were taken at widths of 0.5°. These reflections were used to determine the unit cell using Cell_Now.[19] The unit cell was verified by examination of the *h k l* overlays on several frames of data. No super-cell or erroneous reflections were observed. After careful examination of the unit cell, an extended data collection procedure (26 sets) was initiated using omega and phi scans. Integrated intensity information for each reflection was obtained by reduction of the data frames with APEX2.[20] The integration method employed a three dimensional profiling algorithm and all data were corrected for Lorentz and polarization factors, as well as for crystal decay effects. Finally the data was merged and scaled to produce a suitable data set. SADABS[21] was employed to correct the data for absorption effects. Systematic reflection conditions and statistical tests indicated the space group *P21/c*. A solution was obtained readily using SHELXTL (SHELXS).[22]

Hydrogen atoms were placed in idealized positions and were refined using riding model. All non-hydrogen atoms were refined with anisotropic thermal parameters. The hydride on iridium was assigned from a Q peak near the expected position and refined. The presence of Ir-*H* is also indicated both by ^{1}H NMR spectroscopic data. The structure was refined (weighted least squares refinement on F^2) to convergence.[22,23] Platon[24,25] was used to verify the absence of additional symmetry.

X-Ray data collection, solution, and refinement for 10-Ir-Bpin$_2$ (CCDC 1014978). A yellow, multi-faceted block of suitable size (0.80 x 0.70 x 0.45 mm) was selected from a representative sample of crystals of the same habit using an optical microscope and mounted onto a nylon loop. Low temperature (110 K) X-ray data were obtained on a Bruker APEXII CCD based diffractometer (Mo sealed X-ray tube, K_α = 0.71073 Å). All diffractometer manipulations, including data collection, integration and scaling were carried out using the Bruker APEXII software.[20] An absorption correction was applied using SADABS.[21] The space group was determined on the basis of systematic absences and intensity statistics and the structure was solved by direct methods and refined by full-matrix least squares on F^2. The structure was solved in the monoclinic P21/c space group using XS[22] (incorporated in SHELXTL). All non-hydrogen atoms were refined with anisotropic thermal parameters. All hydrogen atoms were placed in idealized positions and refined using riding model. The structure was refined (weighted least squares refinement on F^2) and the final least-squares refinement converged.

X-Ray data collection, solution, and refinement for 10-Ir-v-tol (CCDC 1014977). A Leica MZ 75 microscope was used to identify a suitable red block with very well defined faces with dimensions (max, intermediate, and min) 0.08 mm x 0.02 mm x 0.02 mm from a representative sample of crystals of the same habit. The crystal mounted on a nylon loop was

then placed in a cold nitrogen stream (Oxford) maintained at 110 K. A BRUKER GADDS X-ray (three-circle) diffractometer was employed for crystal screening, unit cell determination, and data collection. The goniometer was controlled using the FRAMBO software, v.4.1.05.[18] The sample was optically centered with the aid of a video camera such that no translations were observed as the crystal was rotated through all positions. The detector was set at 5.0 cm from the crystal sample. The X-ray radiation employed was generated from a Cu sealed X-ray tube (K_{α} = 1.5418 Å with a potential of 40 kV and a current of 40 mA) fitted with a graphite monochromator in the parallel mode (175 mm collimator with 0.5 mm pinholes). 180 data frames were taken at widths of 0.5°. These reflections were used to determine the unit cell using Cell_Now.[19] The unit cell was verified by examination of the *h k l* overlays on several frames of data. No super-cell or erroneous reflections were observed. After careful examination of the unit cell, an extended data collection procedure (26 sets) was initiated using omega and phi scans. Integrated intensity information for each reflection was obtained by reduction of the data frames with APEX2.[20] The integration method employed a three dimensional profiling algorithm and all data were corrected for Lorentz and polarization factors, as well as for crystal decay effects. Finally the data was merged and scaled to produce a suitable data set. SADABS[21] was employed to correct the data for absorption effects. Systematic reflection conditions and statistical tests indicated the space group *P*-1. A solution was obtained readily (Z' = 2; Z = 4) using SHELXTL (SHELXS).[22] Hydrogen atoms were placed in idealized positions and were refined using riding model. All non-hydrogen atoms were refined with anisotropic thermal parameters. Elongated thermal ellipsoids on (O1-O2-C36 to C41) group suggested disorder which was modeled successfully between two positions. Restraints and constraints were used to keep the bond distances, angles, and the thermal ellipsoids meaningful.[25] The structure was refined (weighted

least squares refinement on F^2) to convergence.[22,23] Platon[24] was used to verify the absence of additional symmetry; however it suggested presence of 50 $Å^3$ voids with no (0 $e^-/Å^3$) electrons in them, which agreed with the difference map showing no electron density in the voids.

X-Ray data collection, solution, and refinement for 10-Ir-p-F_3tol (CCDC 1014979). A Leica MZ 75 microscope was used to identify a suitable orange block with very well defined faces with dimensions (max, intermediate, and min) 0.23 mm x 0.21 mm x 0.13 mm from a representative sample of crystals of the same habit. The crystal mounted on a nylon loop was then placed in a cold nitrogen stream (Oxford) maintained at 110 K. A BRUKER GADDS X-ray (three-circle) diffractometer was employed for crystal screening, unit cell determination, and data collection. The goniometer was controlled using the FRAMBO software, v.4.1.05.[18] The sample was optically centered with the aid of a video camera such that no translations were observed as the crystal was rotated through all positions. The detector was set at 5.0 cm from the crystal sample. The X-ray radiation employed was generated from a Cu sealed X-ray tube (K_α = 1.5418 Å with a potential of 40 kV and a current of 40 mA) fitted with a graphite monochromator in the parallel mode (175 mm collimator with 0.5 mm pinholes). 180 data frames were taken at widths of 0.5°. These reflections were used to determine the unit cell using Cell_Now.[19] The unit cell was verified by examination of the *h k l* overlays on several frames of data. No super-cell or erroneous reflections were observed. After careful examination of the unit cell, a standard data collection procedure (9 sets) was initiated using omega and phi scans. Integrated intensity information for each reflection was obtained by reduction of the data frames with APEX2.[20] The integration method employed a three dimensional profiling algorithm and all data were corrected for Lorentz and polarization factors, as well as for crystal decay effects. Finally the data was merged and scaled to produce a suitable data set. SADABS[21] was employed

to correct the data for absorption effects. Systematic reflection conditions and statistical tests indicated the space group $P2_1/n$. A solution was obtained readily using SHELXTL (SHELXS).[22] A molecule of fluorobenzene was found solvated. Hydrogen atoms were placed in idealized positions and were refined using riding model. All non-hydrogen atoms were refined with anisotropic thermal parameters. Thermal ellipsoids indicated fluorobenzene and CF_3 groups were disordered. While the latter disorder was successfully modeled the former could be only modeled only with strong restraints / constraints. The structure was refined (weighted least squares refinement on F^2) to convergence.[22,23,26] Platon[24] was used to verify the absence of additional symmetry and voids.

IV. Synthesis and Screening of Potential Catalysts for DHBTA.

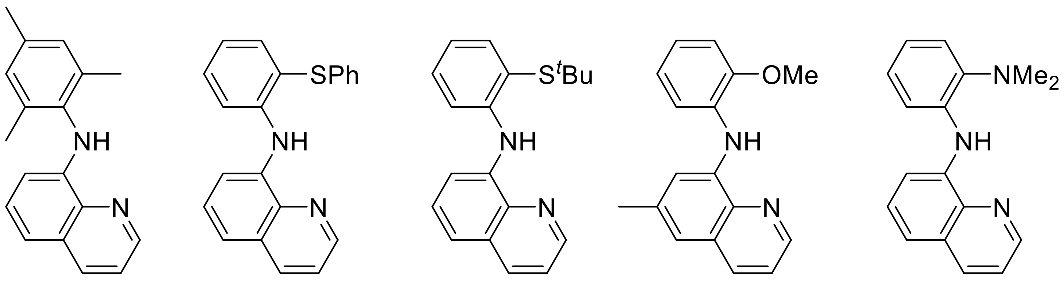

2-H **3-H** **4-H** **5-H** **6-H**

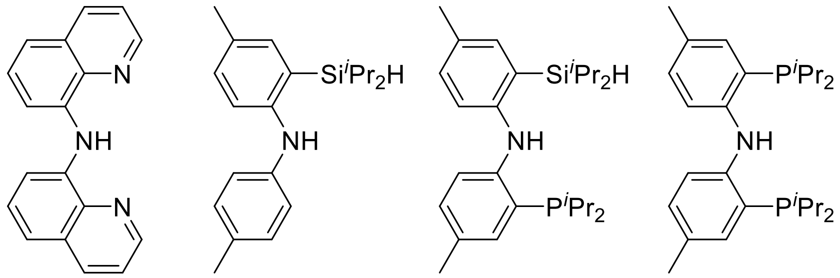

7-H **8-H** **9-H** **10-H**

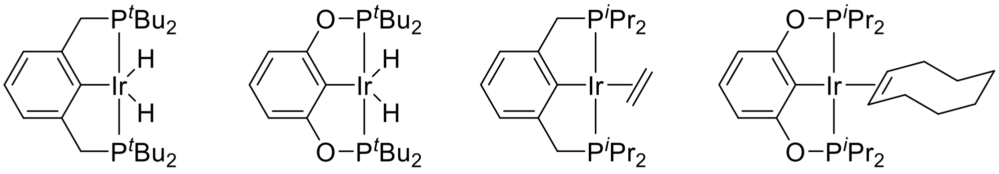

11-Ir-H$_2$ **12-Ir-H$_2$** **13-Ir-C$_2$H$_4$** **14-Ir-COE**

IV-A. Synthesis of Ligands

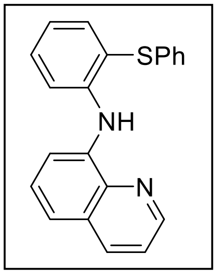

Synthesis of 3-H. In an Ar-filled glove box, $Pd_2(dba)_3$ (180 mg, 0.196 mmol) and BINAP (245 mg, 0.393 mmol) were transferred to a 25 mL PTFE-valved gas-tight flask and dissolved in 5 mL toluene. After stirring for 3 min, 8-bromoquinoline (1.25 mL, 9.58 mmol) and 2-aminophenyl phenyl sulfide (2.35 g, 11.7 mmol) were added to the mixture and stirred for 1 min. Sodium *tert*-butoxide (1.39 g, 14.5 mmol) was then added to the solution with 5 mL toluene to assist in transfer. The flask was taken outside the glovebox and heated at 115 °C for 4 d. After allowing the mixture to cool

to ambient temperature, 0.5 mL of H_2O was added and then the volatiles were removed *in vacuo*. The residue was redissolved with CH_2Cl_2 and filtered through Celite. The volatiles of filtrate were removed *in vacuo* and the residue was purified via column chromatography (1:10 ethyl acetate/hexanes on silica; Rf of **3-H**: 0.58; Rf of 2-aminophenyl phenyl sulfide: 0.48). The volatiles of the eluate were removed *in vacuo* to get a yellow solid. Its ^{1}H NMR spectroscopic analysis indicated >97% purity. Yield: 2.39 g (76%). ^{1}H NMR (500 MHz, $CDCl_3$): δ 8.91 (br s, 1H, N-*H*), 8.65 (m, 1H, Ar-*H*), 7.98 (d, J_{H-H} = 8.0 Hz, 1H, Ar-*H*), 7.68 (d, J_{H-H} = 8.0 Hz, 1H, Ar-*H*), 7.51 (d, J_{H-H} = 8.0 Hz, 1H, Ar-*H*), 7.46 (d, J_{H-H} = 8.0 Hz, 1H, Ar-*H*), 7.30 (m, 3H, Ar-*H*), 7.25 (m, 2H, Ar-*H*, overlapping with the solvent peak), 7.14 (m, 3H, Ar-*H*), 7.04 (m, 1H, Ar-*H*), 6.91 (m, 1H, Ar-*H*). $^{13}C\{^1H\}$ NMR (126 MHz, $CDCl_3$): δ 147.7, 143.2, 139.3, 139.2, 136.2, 136.1, 136.0, 129.8, 129.1, 129.0, 128.9, 127.0, 126.3, 123.4, 121.8, 121.6, 117.9, 117.5, 108.9. HRMS (ESI) calcd. for $C_{21}H_{17}N_2S$ $(M+H)^+$: 329.1112. Found: 329.1107.

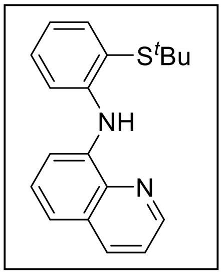

Synthesis of 4-H. In an Ar-filled glove box, $Pd_2(dba)_3$ (22 mg, 0.024 mmol) and BINAP (31 mg, 0.050 mmol) were transferred to a 25 mL PTFE-valved gas-tight flask and dissolved in 3 mL toluene. After stirring 3 min, 8-bromoquinoline (350 µL, 2.68 mmol) and 2-*tert*-butylmercaptoaniline (434 µL, 2.39 mmol) were added to the mixture and stirred for 1 min. Sodium *tert*-butoxide (361 mg, 3.75 mmol) was then added to the solution with 2 mL toluene to assist in transfer. The flask was taken outside the glovebox and heated at 115 °C for 3 d. After allowing the mixture to cool to ambient temperature, 0.5 mL of H_2O was added and then the volatiles were removed *in vacuo*. The residue was purified via flash column chromatography (1:10 ethyl acetate/hexanes on silica). The volatiles of the eluate were removed *in vacuo* to give a yellow oil. Its ^{1}H NMR spectroscopic analysis indicated >98% purity. Yield: 339 mg (46%). ^{1}H NMR (500 MHz, C_6D_6): δ 10.18 (br s,

1H, N-*H*), 8.59 (d, $J_{H\text{-}H}$ = 4.0 Hz, 1H, Ar-*H*), 7.67 (t, $J_{H\text{-}H}$ = 8.0 Hz, 2H, Ar-*H*), 7.59 (d, $J_{H\text{-}H}$ = 8.0 Hz, 1H, Ar-*H*), 7.53 (d, $J_{H\text{-}H}$ = 8.0 Hz, 1H, Ar-*H*), 7.19 (t, $J_{H\text{-}H}$ = 8.0 Hz, 1H, Ar-*H*), 7.13 (d, $J_{H\text{-}H}$ = 8.0 Hz, 1H, Ar-*H*), 6.99 (d, $J_{H\text{-}H}$ = 8.0 Hz, 1H, Ar-*H*), 6.76 (m, 2H, Ar-*H*), 1.27 (s, 9H, SC(C*H*$_3$)$_3$). ^{13}C{^{1}H} NMR (126 MHz, C_6D_6): δ 147.9, 146.1, 140.7, 140.1, 139.8, 135.9, 130.6, 129.4, 127.5, 122.0, 121.7, 120.6, 117.5, 116.7, 108.5, 47.5 (S*C*(CH$_3$)$_3$), 31.1 (SC(*C*H$_3$)$_3$). HRMS (ESI) calcd. for $C_{19}H_{21}N_2S$ (M+H)$^+$: 309.1425. Found: 309.1437.

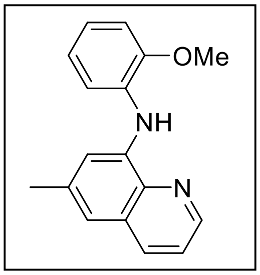

Synthesis of 5-H. In an Ar-filled glove box, 8-bromo-6-methylquinoline (532 mg, 2.40 mmol), 2-methoxyaniline (356 mg, 2.89 mmol) and BINAP (24 mg, 0.038 mmol) were transferred to a 25 mL PTFE-valved gas-tight flask and dissolved in 1.5 mL toluene. After stirring for 1 min, Pd(OAc)$_2$ (5.7 mg, 0.025 mmol Pd) and 0.5 mL toluene were added and stirred for 3 min. Sodium *tert*-pentoxide (379 mg, 3.44 mmol) was then added to the solution with 2 mL toluene to assist in transfer. The flask was taken outside the glovebox and heated at 120 °C for 3 d. All the volatiles were removed *in vacuo* and the residue was purified via column chromatography (1:8 ethyl acetate/hexanes on silica) to yield a yellow oil. Its ^{1}H NMR spectroscopic analysis indicated >98% purity. Yield: 565 mg (89%). ^{1}H NMR (500 MHz, $CDCl_3$): δ 8.75 (dd, $J_{H\text{-}H}$ = 4.0, 1.5 Hz, 1H), 8.50 (s, 1H, N-*H*), 7.99 (dd, $J_{H\text{-}H}$ = 8.0, 1.5 Hz, 1H, Ar-*H*), 7.69 (d, $J_{H\text{-}H}$ = 8.0 Hz, 1H, Ar-*H*), 7.42 (s, 1H, Ar-*H*), 7.36 (dd, $J_{H\text{-}H}$ = 8.0, 4.0 Hz, 1H, Ar-*H*), 6.99 (m, 4H, Ar-*H*), 3.94 (s, 3H, OC*H*$_3$), 2.48 (s, 3H, Ar-C*H*$_3$). ^{13}C{^{1}H} NMR (126 MHz, $CDCl_3$): δ 150.3, 146.8, 139.5, 138.1, 137.3, 135.5, 131.6, 129.1, 121.7, 121.4, 120.7, 117.5, 116.0, 110.9, 110.2, 55.8 (O*C*H$_3$), 22.5 (Ar-*C*H$_3$). HRMS (ESI) calcd. for $C_{17}H_{17}N_2O$ (M+H)$^+$: 265.1341. Found: 265.1332.

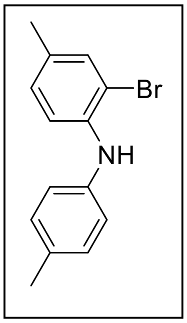

Synthesis of S1. In an Ar-filled glovebox, bis(2-bromo-4-methylphenyl)amine (8.03 g, 22.6 mmol) was dissolved in 80 mL Et_2O in a 250 mL Schlenk flask. The flask was then taken outside of glovebox, connected to a Schlenk line under Ar atomosphere, and placed in a -45 °C dry ice/acetone cooling bath to result in a slurry. *n*-BuLi (18.1 mL of 2.5 M solutions in hexanes, 45.3 mmol) was added dropwise over the course of 30 min via syringe. The solution color turned yellow and then white precipitate formed. After addition of *n*-BuLi, the solution was left to stir at -45 °C for 30 min further and the mixture was allowed to warm to ambient temperature. After stirring at room temperature for 1 h, the flask was placed in a 0 °C ice bath and water (3.0 mL, 166 mmol) was slowly added in 1 min via syringe. The volatiles were removed *in vacuo* to result in yellow liquid with a white precipitate. TLC analysis revealed that the liquid contained ~90% of desired product and ~10% di-4-tolylamine. The liquid part was purified via column chromatography (hexanes on silica, Rf of **S1**: 0.61, Rf of di-4-tolylamine: 0.13). The volatiles of eluate were removed to get a slightly yellow liquid. Its ^{1}H NMR spectroscopic analysis indicated >98% purity. Yield: 5.39 g (86%). ^{1}H NMR (500 MHz, C_6D_6): δ 7.22 (s, 1H, Ar-*H*), 7.05 (d, J_{H-H} = 8.0 Hz, 2H, Ar-*H*), 6.88 (d, J_{H-H} = 8.0 Hz, 2H, Ar-*H*), 6.80 (d, J_{H-H} = 8.0 Hz, 1H, Ar-*H*), 6.68 (d, J_{H-H} = 8.0 Hz, 1H, Ar-*H*), 5.80 (br s, 1H, N-*H*), 2.10 (s, 3H, Ar-CH_3), 1.93 (s, 3H, Ar-CH_3). ^{13}C{^{1}H} NMR (126 MHz, C_6D_6): δ 140.2, 140.0, 133.6, 132.0, 130.5, 130.2, 129.1, 120.9, 116.3, 112.5, 20.8 (Ar-*C*H_3), 20.1 (Ar-*C*H_3).

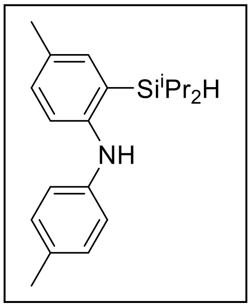

Synthesis of 8-H. In an Ar-filled glovebox, **S1** (1.00 g, 3.62 mmol) was dissolved in 20 mL Et_2O in a 50 mL Schlenk flask. *n*-BuLi (3.19 mL of 2.5 M solutions in hexanes, 7.98 mmol) was added dropwise over the course of 10 min via syringe. The solution color evolved from colorless to yellow and

then a white precipitate formed. After addition of *n*-BuLi, the solution was left to stir at ambient temperature for 2 h further, and $^{i}Pr_2SiHCl$ (1.42 mL, 8.32 mmol) was then added dropwise over the course of 10 min. The solution was left to stir at ambient temperature for 15 h. The flask was then taken outside of glovebox, 5 mL 1M $HCl_{(aq)}$ was added to the flask and the solution was left to stir at ambient temperature for 2 h. $NaHCO_3$ was added to neutralize the solution and remove all volatiles *in vacuo*. The liquid part of the residue was purified through column chromatography (hexanes on silica, Rf of **8-H**: 0.39). The volatiles of the eluate were removed to give a colorless liquid. Its ^{1}H NMR spectroscopic analysis indicated >98% purity. Yield: 827 mg (73%). ^{1}H NMR (500 MHz, C_6D_6): δ 7.35 (s, 1H, Ar-*H*), 7.27 (d, $J_{H\text{-}H}$ = 8.5 Hz, 1H, Ar-*H*), 6.96 (d, $J_{H\text{-}H}$ = 8.5 Hz, 1H, Ar-*H*), 6.93 (d, $J_{H\text{-}H}$ = 8.5 Hz, 2H, Ar-*H*), 6.84 (d, $J_{H\text{-}H}$ = 8.5 Hz, 2H, Ar-*H*), 5.64 (br s, 1H, N-*H*), 4.26 (t, $J_{Si\text{-}H}$ = 178 Hz, $J_{H\text{-}H}$ = 3.5 Hz, 1H, Si-*H*), 2.16 (s, 3H, Ar-CH_3), 2.12 (s, 3H, Ar-CH_3), 1.19 (m, 2H, C*H*Me_2), 1.08 (d, $J_{H\text{-}H}$ = 7.5 Hz, 6H, CHMe_2), 1.03 (d, $J_{H\text{-}H}$ = 7.5 Hz, 6H, CHMe_2). $^{13}C\{^{1}H\}$ NMR (126 MHz, C_6D_6): δ 147.2, 142.7, 137.8, 131.7, 130.9, 130.2, 129.7, 125.2, 120.0, 117.7, 20.9 (Ar-*C*H_3), 20.7 (Ar-*C*H_3), 19.3 (CHMe_2), 19.1 (CHMe_2), 11.3 (*C*HMe_2). ^{29}Si NMR (79 MHz, C_6D_6): δ 0.6 (d, $J_{Si\text{-}H}$ = 178 Hz). HRMS (ESI) calcd. for $C_{20}H_{30}NSi$ $(M+H)^+$: 312.2148. Found: 312.2139.

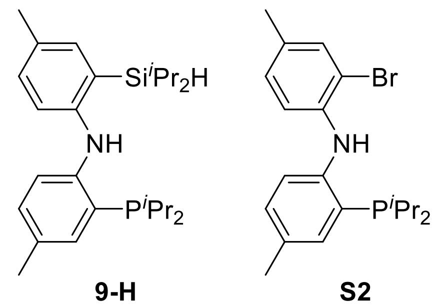

9-H **S2**

Synthesis of 9-H. A 100 mL Schlenk flask was charged with **S2** (515 mg, 1.31 mmol), diethyl ether (20 mL), and a stir bar. *n*-BuLi (1.10 mL of 2.5 M solutions in hexanes, 2.75 mmol) was added dropwise via syringe to a stirring solution. Stirred was continued for 1 h and then $^{i}Pr_2SiHCl$ (250 µL, 1.46 mmol) was added slowly via syringe. The solution became murky and yellow. The reaction mixture was then stirred for 12 h and prior to being quenched with degassed H_2O

(50 µL). The volatiles were then removed *in vacuo* and the resulting residue was dissolved in pentane and filtered through Celite. All volatiles were removed *in vacuo* to yield a white solid. Its ^{1}H NMR spectroscopic analysis indicated 95% purity. Yield: 479 mg (85%). The material was further recrystallized from pentane prior to testing its catalytic reactivity in DHBTA. ^{1}H NMR (500 MHz, C_6D_6): δ 7.48 (m, 2H, Ar-*H*), 7.37 (d, J_{H-H} = 8.2 Hz, 1H, Ar-*H*), 7.19 (dd, J_{H-H} = 8.4 Hz, J_{P-H} = 5.0 Hz, 1H, Ar-*H*), 7.13 (s, 1H, N-*H*), 6.97 (dd, J_{H-H} = 8.2, 2.0 Hz, 1H, Ar-*H*), 6.88 (dd, J_{H-H} = 8.4, 2.0 Hz, 1H, Ar-*H*), 4.46 (t, J_{Si-H} = 183 Hz, J_{H-H} = 4.0 Hz, 1H, Si-*H*), 2.18 (s, 3H, Ar-*Me*), 2.17 (s, 3H, Ar-*Me*), 1.97 (m, 2H, P(C*H*Me$_2$)$_2$), 1.47 (m, 2H, Si(C*H*Me$_2$)$_2$), 1.21 (d, J_{H-H} = 7.4 Hz, 6H, Si(CH*Me$_2$*)$_2$), 1.15 (d, J_{H-H} = 7.4 Hz, 6H, Si(CH*Me$_2$*)$_2$), 1.11 (dd, J_{P-H} = 15 Hz, J_{H-H} = 7.0 Hz, 6H, P(CH*Me$_2$*)$_2$), 0.98 (dd, J_{P-H} = 12 Hz, J_{H-H} = 7.0 Hz, 6H, P(CH*Me$_2$*)$_2$). ^{13}C{^{1}H} NMR (126 MHz, C_6D_6): δ149.1 (d, J_{C-P} = 19 Hz), 147.0, 138.6, 138.6, 133.5, 133.5, 131.6, 131.5, 131.0, 121.0, 120.4 (d, J_{P-C} = 15 Hz), 115.7, 23.6 (d, J_{P-C} = 10 Hz, P(CH*Me$_2$*)$_2$), 20.9 (Ar-*C*H$_3$), 20.8 (Ar-*C*H$_3$), 20.4, (d, J_{P-C} = 19 Hz, P(*C*HMe$_2$)$_2$), 19.6 (Si(CH*Me$_2$*)$_2$), 19.5 (Si(CH*Me$_2$*)$_2$), 19.2 (d, J_{P-C} = 9.0 Hz, P(CH*Me$_2$*)$_2$), 11.6 (Si(*C*HMe$_2$)$_2$). ^{31}P{^{1}H} NMR (202 MHz, C_6D_6): δ -15.4. ^{29}Si NMR (79 MHz, C_6D_6): δ 5.2 (d, J_{H-Si} = 183 Hz). HRMS (ESI) calcd. for $C_{26}H_{43}NPSi$ $(M+H)^+$: 428.2902. Found: 428.2908.

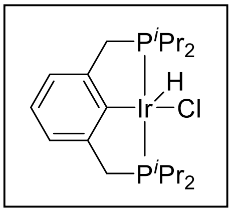

Synthesis of 13-Ir-HCl. **13-H** (176 mg, 0.521 mmol) was placed into a vial and diluted with 20 mL of toluene. This solution was transferred dropwise over five minutes to a 100 mL Hi-Vac valve round bottom flask containing $[(COE)_2IrCl]_2$ (234 mg, 0.261 mmol) in 30 mL of toluene. The orange solution was then placed in an oil bath at 110 °C. After 1 h, solution was dark red. The reaction was left to stir at 110 °C for a total of 14 h. After removing from the heat, ^{1}H and ^{31}P NMR analysis of an aliquot of the reaction mixture demonstrated there was no free ligand present and that the mixture was

composed of approximately 85% of the desired product. The solution was filtered over a pad of Celite and the volatiles were removed under vacuum, yielding a red-orange solid. To isolate of pure portion of **13-Ir-HCl**, a crude pipet silica gel column separation was performed. After removing volatiles, pentane was used to extract and transfer the solution to a pipet column with a plug of Celite and a tall silica gel layer. The silica gel became dark red and it was washed several times with pentane. Approximately 10 mL of a 12:1 pentane:toluene mixture was used to wash the product from the toluene to collect a 10 mL fraction. The percentage of toluene in the eluent was gradually increased and 5 more fractions were collected until the column was rinsed with pure toluene. NMRs of each of the fractions were taken and the initial fractions containing the cleanest product were combined. NMR of the red solid which remained undissolved from the initial pentane transferred also showed clean product. Overall, 81.2 mg (yield 22%) of greater than 99% pure **13-Ir-HCl** was collected. ^{1}H NMR (300 MHz, C_6D_6): δ 6.95 (br s, 3 H, Ar-*H*), 2.79 (m, 6 H, -C*H*$_2$-P overlapped with one set of P-C*H*(CH_3)$_2$), 1.96 (m, 2H, P-C*H*(CH_3)$_2$), 1.20 (m, 12H, -CH(C*H*$_3$)$_2$), 0.90 (dvt, $J_{P\text{-}H}$ = 7 Hz, $J_{H\text{-}H}$ = 7 Hz, 12H, -CH(C*H*$_3$)$_2$), -36.24 (t, $J_{P\text{-}H}$ = 13 Hz, Ir-*H*). ^{13}C{^{1}H} NMR (75 MHz, C_6D_6): δ 149.7 (vt, $J_{P\text{-}C}$ = 9 Hz), 141.1 (s), 122.6 (s), 122.1 (vt, $J_{P\text{-}C}$ = 8 Hz), 33.4 (vt, $J_{P\text{-}C}$ = 15 Hz, -*C*H$_2$-), 24.6 (vt, $J_{P\text{-}C}$ = 15 Hz, -*C*H(CH_3)$_2$), 18.7 (vt, $J_{P\text{-}C}$ = 2Hz, -CH(*C*H$_3$)$_2$), 18.6 (m, -CH(*C*H$_3$)$_2$), 17.6 (s, -CH(*C*H$_3$)$_2$). ^{31}P{^{1}H} NMR (121 MHz, C_6D_6): δ 58.4 (s). Anal. Calcd for $C_{20}H_{36}ClIrP_2$: C, 42.43; H, 6.41. Found: C, 42.57; H, 6.61.

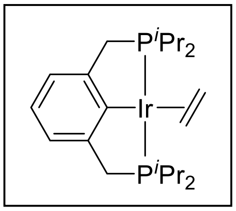

Synthesis of 13-Ir-C_2H_4. 13-Ir-HCl (70.9 mg, 0.125 mmol) and NaO^{t}Bu (14.8 mg, 0.154 mmol) were transferred into a 100 mL Hi-Vac valve round bottom flask. Approximately 10 mL of toluene was added. The solution was degassed then 1 atm of ethylene was added to the stirring brown solution. After stirring for about 30 min, the volatiles were removed and pentane was used to extract the product from the residual

brown solid. The pentane solution was filtered over a pad of Celite, the Celite was washed with about 2 mL of isooctane, and then the volatiles were removed under vacuum. The resulting brown solid was dried under vacuum and 46 mg (66% yield) was collected. ^{1}H NMR (400 MHz, C_6D_6): δ 7.35 (d, J_{H-H} = 7 Hz, 2H, Ar-*H*), 7.26 (t, J_{H-H} = 7 Hz, 1H, Ar-*H*), 3.06 (t, J = 4 Hz, 4H), 2.98 (m, 4H), 2.01 (m, 4H), 1.03 (dvt, J_{P-H} = 7 Hz, J_{H-H} = 7 Hz, 12H, -CH(C*H*$_3$)$_2$), 0.88 (dvt, J_{P-H} = 7 Hz, J_{H-H} = 7 Hz, 12H, -CH(C*H*$_3$)$_2$). ^{13}C{^{1}H} NMR (75 MHz, C_6D_6): δ 178.0 (t, J_{P-C} = 4 hz), 152.6 (vt, J_{P-C} = 11 Hz), 124.9 (s), 120.6 (vt, J_{P-C} = 11 Hz), 38.8 (vt, J_{P-C} = 15 Hz), 36.0 (br s), 25.0 (m), 18.7, 18.4. ^{31}P{^{1}H} NMR (121 MHz, C_6D_6): δ 51.4 (br s). Anal. Calcd for $C_{22}H_{39}IrP_2$: C, 47.38; H, 7.05. Found: C, 47.25; H, 6.98.

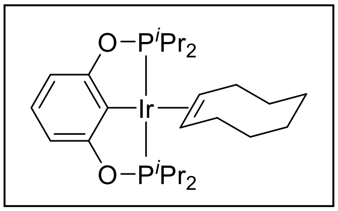

Synthesis of 14-Ir-COE. **14-Ir-HCl** (35 mg, 0.061 mmol) was dissolved in 0.7 mL C_6D_6 in J. Young NMR tube resulting in a dark red solution. NaO^{t}Bu (7.2 mg, 0.075 mmol) and cyclooctene (20 µL, 0.15 mmol) were added and the NMR tube was inverted several times to mix the solution. The solution remained red and ^{31}P and ^{1}H NMR after 10 min showed full conversion to the desired product. The sample was filtered over a pad of Celite and volatiles were removed *in vacuo* yielding a red oily solid. After two cycles of dissolving the oily solid in isooctane and removing volatiles, red solid was obtained. Yield: 23 mg (58%). ^{1}H NMR (300 MHz, C_6D_6): δ 7.03 (t, J_{H-H} = 7 Hz, 1H, Ar-*H*), 6.94 (d, J_{H-H} = 7 Hz, 2H, Ar-*H*), 3.97 (br d, J = 8 Hz, 2H, alkenyl-*H* on COE), 2.44 (br d, J = 12 Hz, 2H), 2.22 (m, 4H), 1.74 (br m, 6H), 1.46 (br m, 4H), 1.13 (m, 24 H, , PCH(C*H*$_3$)$_2$). ^{13}C{^{1}H} NMR (126 MHz, C_6D_6): δ 168.4 (vt, J_{P-C} = 8 Hz), 145.7 (t, J_{P-C} = 8 Hz), 128.8, 103.5 (vt, J_{P-C} = 6 Hz), 62.5 (alkenyl-*C* on COE), 34.9 (t, J_{P-C} = 3 Hz), 33.5, 30.6 (t, J_{P-C} = 14 Hz), 27.1, 18.0 (t, J_{P-C} = 3 Hz), 17.1. ^{31}P{^{1}H} NMR (121 MHz, C_6D_6): δ 174.2 (s). Anal. Calcd for $C_{26}H_{45}IrO_2P_2$: C, 48.51; H, 7.05. Found: C, 48.36; H, 7.19.

IV-B. Ligand Screening for DHBTA

(1) For central amido donor ligands (2-H to 10-H):

In an Ar-filled glove box, a ligand (40 µL of 0.025 M stock solution in C_6D_6, 0.0010 mmol) and sodium bis(trimethylsilyl)amide (40 µL of 0.025 M stock solution in C_6D_6, 0.0010 mmol) were mixed in a vial for 10 s. $[(COE)_2IrCl]_2$ (40 µL of 0.0125 M stock solution in C_6D_6, 0.00050 mmol) was added to the vial and mixed for 10 s, and the mixture was then transferred to a J. Young tube. Pinacolborane (200 µL of 1.0 M pinacolborane stock solution in C_6D_6, 0.20 mmol) was added to the J. Young tube. 4-ethynyltoluene (**A1-H**)/1,4-dioxane (200 µL of 0.50 M 4-ethynyltoluene (**A1-H**)/ 0.35 M 1,4-dioxane stock solution in C_6D_6, 0.10 mmol for 4-ethynyltoluene and 0.070 mmol for 1,4-dioxane) were then added in 4 portions with 1 min intervals. The J. Young tube was taken outside the glovebox after 10 min, and the reaction yield was determined by ^{1}H NMR spectroscopy using 1,4-dioxane as an internal standard. The reagent ratio was calibrated through the integration ratio from a ^{1}H NMR spectrum of a sample obtained by mixing 100 µL of the pinacolborane stock solution and 100 µL 4-ethynyltoluene (**A1-H**)/1,4-dioxane stock solution in 0.3 mL C_6D_6 in another J. Young tube without adding other reagents.

(2) For central aryl donor ligands (11-Ir-H_2, 12-Ir-H_2, 13-Ir-C_2H_4 and **14-Ir-COE):**

In an Ar-filled glove box, an iridium complex (80 µL of 0.0125 M stock solution in C_6D_6, 0.0010 mmol) was transferred to a J. Young tube. Pinacolborane (200 µL of 1.0 M pinacolborane stock solution in C_6D_6, 0.20 mmol) was added to the J. Young tube. 4-ethynyltoluene (**A1-H**)/1,4-dioxane (200 µL of 0.50 M 4-ethynyltoluene / 0.35 M 1,4-dioxane stock solution in C_6D_6, 0.10 mmol for 4-ethynyltoluene (**A1-H**) and 0.070 mmol for 1,4-dioxane) was then added in 4 portions with 1 min intervals. The J. Young tube was taken outside the glovebox after 10 min, and the reaction yield was determined by ^{1}H NMR spectroscopy using 1,4-dioxane as an internal

standard. The reagent ratio was calibrated through the integration ratio from a ^{1}H NMR spectrum of a sample obtained by mixing 100 µL of the pinacolborane stock solution and 100 µL 4-ethynyltoluene (**A1-H**)/1,4-dioxane stock solution in 0.3 mL C_6D_6 in another J. Young tube without adding other reagents.

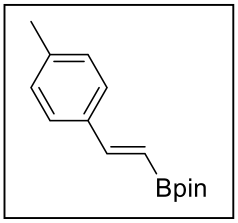

A1-1. ^{1}H NMR (500 MHz, C_6D_6): δ 7.75 (d, J_{H-H} = 18 Hz, 1H, alkenyl-*H*), 7.27 (d, J_{H-H} = 8.0 Hz, 2H, Ar-*H*), 6.85 (d, J_{H-H} = 8.0 Hz, 2H, Ar-*H*), 6.42 (d, J_{H-H} = 18 Hz, 1H, alkenyl-*H*), 2.01 (s, 3H, Ar-CH_3), 1.12 (s, 12H, CH_3 on Bpin).

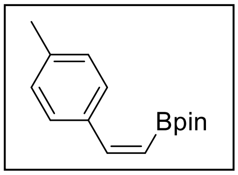

A1-2. ^{1}H NMR (500 MHz, C_6D_6): δ 7.61 (d, J_{H-H} = 8.0 Hz, 2H, Ar-*H*), 7.17 (d, J_{H-H} = 15 Hz, 1H, alkenyl-*H*), 7.00 (d, J_{H-H} = 8.0 Hz, 2H, Ar-*H*), 5.78 (d, J_{H-H} = 15 Hz, 1H, alkenyl-*H*), 2.09 (s, 3H, Ar-CH_3), 1.06 (s, 12H, CH_3 on Bpin).

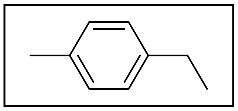

A1-3. ^{1}H NMR (500 MHz, C_6D_6): δ 7.00 (s, 4H, Ar-*H*), 2.46 (q, J_{H-H} = 7.6 Hz, 2H, Ar-CH_2CH$_3$), 2.14 (s, 3H, Ar-CH_3), 1.10 (t, J_{H-H} = 7.6 Hz, 3H, Ar-CH$_2$CH_3).

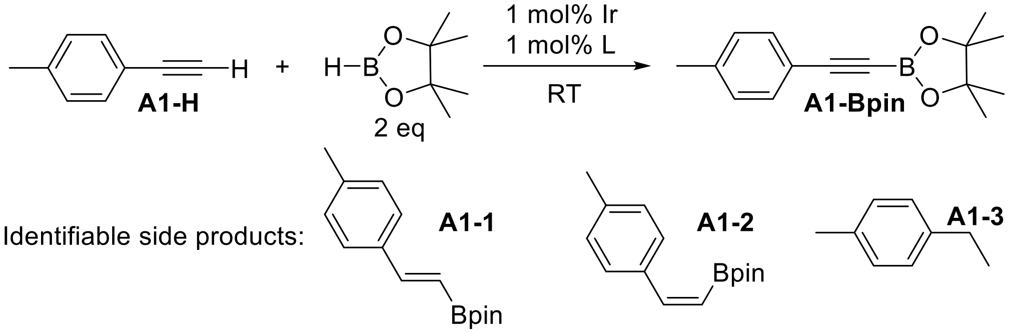

Ligand or Complex	10 min		1 h	
	Yield (**A1-Bpin**)	Conversion[a] (alkyne)	Yield (**A1-Bpin**)	Conversion[a] (alkyne)
**2-H**	0%	8%	0%	18%
**3-H**	0%	8%	0%	10%
**4-H**	0%	10%	0%	13%
**5-H**	0%	11%	0%	16%
**6-H**	0%	8%	0%	15%
**7-H**	0%	6%	0%	10%
**8-H**	0%	13%	0%	21%
**9-H**	0%	10%	0%	21%
**10-H**	76%[b]	82%	90%[c]	100%
**11-Ir-H$_2$**	0%[d]	100%	-	-
**12-Ir-H$_2$**	0%[e]	16%	0%[f]	52%
**13-Ir-C$_2$H$_4$**	0%	11%	0%	32%
**14-Ir-COE**	0%	3%	0%	6%

[a] The conversion was determined by ^{1}H NMR by the ratio of integration of Ar-C*H$_3$* on **A-0** to the internal standard. [b] 2% **A-3** was observed. [c] 3% **A-3** was observed. [d] 52% **A-1** and 39% **A-2** were observed. [e] 8% **A-1** and 3% **A-2** were observed. [f] 31% **A-1** and 13% **A-2** were observed.

Attempted catalysis of the reaction of pinacolborane with 4-ethynyltoluene by [(COD)Ir(OMe)]$_2$/dtbpy.

[(COD)Ir(OMe)]$_2$ (4.4 mg, 0.0066 mmol), 4,4'-di-*tert*-butyl bipyridne (3.6 mg, 0.013 mmol), hexamethylbenzene (17.0 mg, 0.105 mmol) were loaded in a J. Young tube, and pinacolborane

(156 µL, 1.08 mmol) and 0.2 mL cyclohexane-d_{12} were then added. After mixing for 1 min, 4-ethynyltoluene (68 µL, 0.54 mmol) in 0.2 mL cyclohexane-d_{12} was added in 4 portions in 1 min interval. After RT for 1 h, analysis by ^{1}H NMR spectroscopy revealed 4-ethynyltoluene and pinacolborane remained intact.

V. Synthesis of (PNP)Ir Complexes and Examination of their performance in DHBTA.

Synthesis of (PNP)Ir Complexes with Various Phosphine Substituents

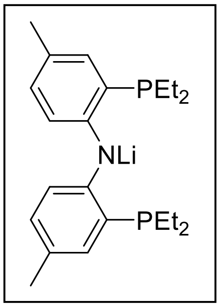

15-Li. In a 100 mL Schlenk flask under Ar atmosphere, bis(2-bromo-4-methylphenyl)amine (1.31 g, 3.69 mmol) was dissolved in 30 mL of Et_2O. The solution was placed in the glovebox freezer for 2 h at -35 °C. *n*-BuLi (5.00 mL of 2.5 M solution in hexanes, 12.5 mmol) was added and the solution was stirred overnight after which some white precipitate was observed. The solution was cooled to -35 °C in the freezer for 1 h, then Et_2PCl (1.66 mL, 13.6 mmol) was added. The mixture immediately became bright yellow-orange gradually fading to light yellow. After 3 h, the reaction was quenched with 200 μL degassed H_2O/MeOH 1:1 soln. The resultant solution was stirred for 20 min, then volatiles were removed. The residue was extracted with Et_2O then filtered over Celite yielding a pale yellow solution. Volatiles were reduced leaving a yellow oil. The oil was diluted in pentane then filtered over Celite, and all volatiles were removed *in vacuo*. 10 mL pentane was added to dissolve the residue, and *n*-BuLi (1.8 mL of 2.5 M solution in hexanes, 4.5 mmol) was added dropwise over 5 min. The solution color turned bright yellow immediately and a fine yellow powder formed. The solution was stirred for 1 h at RT and then filtered through a fritted funnel. The yellow powder was washed with cold pentane and dried *in vacuo*. Its ^{1}H NMR spectroscopic analysis indicated >98% purity. Yield: 785 mg (56%). ^{1}H NMR (500 MHz, C_6D_6): δ 7.03 (dd, 2H, $J_{H\text{-}H}$ = 8.5 Hz, $J_{P\text{-}H}$ = 5.5 Hz, Ar-*H*), 6.99 (m, 2H, Ar-*H*), 6.93 (dd, 2H, $J_{H\text{-}H}$ = 8.5, 2.0 Hz, Ar-*H*), 2.24 (s, 6H, Ar-CH_3), 1.48 (m, 4H, -CH_2CH$_3$), 1.19 (m, 4H, -CH_2CH$_3$), 0.88 (m, 12H, -CH$_2$CH_3). ^{13}C{^{1}H} NMR (126 MHz, C_6D_6): δ 167.2 (m), 132.3, 131.4, 126.1, 126.0, 125.5, 20.7, 19.8 (br), 10.6, 10.5. ^{31}P{^{1}H} NMR (202 MHz, C_6D_6): δ -26.9 (br).

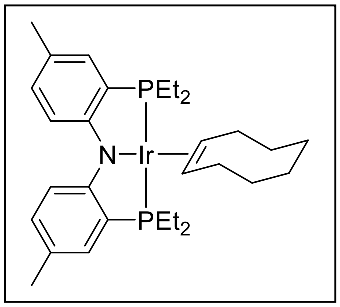

15-Ir-COE. $[(COE)_2IrCl]_2$ (130 mg, 0.145 mmol) was dissolved in 2 mL fluorobenzene in a 25 mL Schlenk flask and resulted an orange solution. Solution of **15-Li** (109 mg, 0.288 mmol) in 1 mL PhF was added dropwise over 30 s and the mixture turned clear deep orange. After 10 min, the solution was filtered through Celite and all volatiles were removed *in vacuo*. 5 mL pentane was added to the flask and a spatula was used to dislodge the orange powder from the walls of the flask. The solution was vigorously stirred for 1 h and filtered through a fritted funnel to collect the powdery material which was washed with cold pentane and dried *in vacuo* to yield an orange solid. Its ^{1}H NMR spectroscopic analysis indicated >98% purity. Yield: 125 mg (64%). ^{1}H NMR (500 MHz, C_6D_6): δ 7.82 (d, $J_{H\text{-}H}$ = 8.0 Hz, 2H, Ar-*H*), 6.85 (m, 4H, Ar-*H*), 2.72 (m, 2H, alkenyl-*H* on COE), 2.39 (m, 2H), 2.21 (s, 6H, Ar-C*H*$_3$) 1.89 (m, 2H), 1.59 (m, 16H), 1.00 (m, 12H, P(CH$_2$C*H*$_3$)$_2$).^{13}C{^{1}H} NMR (126 MHz, C_6D_6): δ 164.4, 131.7, 130.6, 128.6, 126.2, 115.5, 42.6, 33.1 (t, $J_{P\text{-}C}$ = 3.8 Hz), 32.9, 27.4, 22.9 (br), 20.4, 17.7 (br), 9.2. ^{31}P{^{1}H} NMR (121 MHz, C_6D_6): δ 19.6 (d, J = 15 Hz). Anal. Calcd for $C_{30}H_{46}IrNP_2$: C, 53.39; H, 6.87. Found: C, 53.25; H, 6.83.

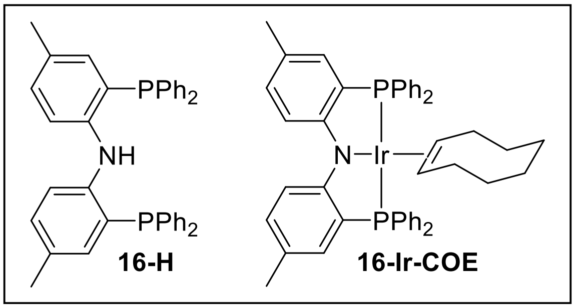

16-Ir-COE. 16-H (175 mg, 0.310 mmol) and $NaN(TMS)_2$ (57 mg, 0.31 mmol) were dissolved in 10 mL toluene in a 50 mL Schlenk flask to result in a yellow solution. $[(COE)_2IrCl]_2$ (138 mg, 0.154 mmol) in 5 mL toluene was added to the flask dropwise over 3 min. Solution color turned orange during the addition. After 1 h, the solution was filtered through Celite and silica gel, and all volatilves were removed in vacuo. 5

mL Et_2O was added to the flask and a spatula was used to scratch the filmy solid off the walls of the flask to produce an orange powder. The solution was vigorously stirred for 1 h and then filtered through a fritted funnel to collect the powder which was washed with cold Et_2O and dried *in vacuo* to yield an orange solid. Its ^1H NMR spectroscopic analysis indicated >95% purity. Yield: 199 mg (74%). The material was further recrystallized from CH_2Cl_2/Et_2O prior to testing its catalytic reactivity in DHBTA. The ^1H NMR (500 MHz, C_6D_6) spectral data were in agreement with those reported in the literature.[27] ^1H NMR (400 MHz, CD_2Cl_2): δ 7.82 (br, 8H, Ar-*H*), 7.43 (m, 12H, Ar-*H*), 7.34 (dt, $J_{H\text{-}H}$ = 8.5 Hz, $J_{P\text{-}H}$ = 2.7 Hz, 2H, Ar-*H*), 6.92 (td, $J_{P\text{-}H}$ = 4.8 Hz, $J_{H\text{-}H}$= 1.8 Hz, 2H, Ar-*H*), 6.83 (dd, $J_{H\text{-}H}$ = 8.5, 1.8 Hz, 2H, Ar-*H*), 2.62 (m, 2H, alkenyl-*H* on COE), 2.12 (s, 6H, Ar-C*H_3*), 1.76 (m, 2H), 1.20 (m, 2H), 1.08 (m, 2H), 0.94 (m, 2H), 0.78 (m, 4H). $^{13}C\{^1H\}$ NMR (100 MHz, CD_2Cl_2): δ 161.8, 134.2 (br, 4C), 133.1, 131.9, 130.3, 128.6 (t, $J_{P\text{-}C}$ = 4.6 Hz, 4C), 127.1 (t, $J_{P\text{-}C}$ = 3.4 Hz), 115.1, 50.1, 32.2, 31.7 (t, $J_{P\text{-}C}$ = 3.4 Hz), 26.6, 20.2. $^{31}P\{^1H\}$ NMR (121 MHz, CD_2Cl_2): δ 28.1 (br).

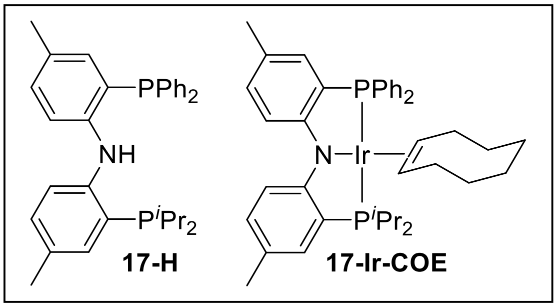

17-Ir-COE. 17-H (202 mg, 0.406 mmol) and $NaN(TMS)_2$ (74 mg, 0.41 mmol) were dissolved in 10 mL toluene in a 50 mL Schlenk flask to result in a yellow solution. $[(COE)_2IrCl]_2$ (182 mg, 0.203 mmol) in 5 mL toluene was added to the flask dropwise over 3 min. Solution color turned orange during the addition. After 1 h, the solution was filtered through Celite and silica gel, and all volatilves were removed in vacuo. 5 mL Et_2O was added to the flask and a spatula was used to dislodge the filmy solid off the walls of the flask to result in an orange powder. The solution was vigorously stirred for 1 h and filtered through a fritted funnel to collect the powder, which was washed with cold Et_2O and dried *in vacuo* to

yield an orange solid. Its ^{1}H NMR spectroscopic analysis indicated >95% purity. Yield: 223 mg (69%). The material was further recrystallized from CH_2Cl_2/Et_2O prior to testing its catalytic reactivity in DHBTA. ^{1}H NMR (500 MHz, C_6D_6): δ 8.13 (br, 4H, Ar-*H*), 7.61 (dd, J_{H-H} = 8.5 Hz, J_{P-H} = 5.0 Hz, 1H, Ar-*H*), 7.46 (dd, J_{H-H} = 8.5 Hz, J_{P-H} = 4.0 Hz, 1H, Ar-*H*), 7.12 (d, J_{H-H} = 8.5 Hz, 1H, Ar-*H*), 7.03 (m, 6H, Ar-*H*), 6.92 (d, J_{H-H} = 8.5 Hz, 1H, Ar-*H*), 6.74 (d, J_{H-H} = 8.5 Hz, 1H, Ar-*H*), 6.69 (d, J_{H-H} = 8.5 Hz, 1H, Ar-*H*), 3.48 (br, 2H, alkenyl-*H* on COE), 2.40 (m, 2H), 2.18 (m, 5H, included Ar-CH_3), 2.04 (s, 3H, Ar-CH_3), 1.61 (m, 2H), 1.42 (m, 2H), 1.33 (dvt, J = 14, 7.0 Hz, 6H, CHMe_2), 1.13 (m, 8H, included CHMe_2), 1.00 (br, 2H).$^{13}C\{^{1}H\}$ NMR (75 MHz, C_6D_6): δ 164.3 (dd, J_{P-C} = 16, 4.8 Hz), 162.5 (dd, J_{P-C} = 18, 3.4 Hz), 136.4 (d, J = 7.8 Hz), 135.9 (d, J_{P-C} = 7.8 Hz), 135.0 (d, J_{P-C} = 9.5 Hz), 132.7, 131.9, 131.6, 129.8, 128.2, 126.4 (d, J_{P-C} = 6.5 Hz), 126.0 (d, J_{P-C} = 6.5 Hz), 123.1 (d, J_{P-C} = 3.8 Hz), 122.5 (d, J_{P-C} = 3.8 Hz), 116.7 (d, J_{P-C} = 9.4 Hz), 114.3 (d, J_{P-C} = 9.1 Hz), 44.4, 32.8, 32.4 (d, J_{P-C} = 5.4 Hz), 27.1, 24.0 (d, J_{P-C} = 22 Hz), 20.5, 20.3, 18.8, 17.5. $^{31}P\{^{1}H\}$ NMR (202 MHz, C_6D_6): δ 31.2 (d, J_{P-P} = 360 Hz), 27.2 (d, J_{P-P} = 360 Hz). Anal. Calcd for $C_{40}H_{50}IrNP_2$: C, 60.13; H, 6.31. Found: C, 59.91; H, 6.27.

General procedure for the NMR-scale dehydrogenative borylation of alkynes (Table 1) In an Ar-filled glove box, a solution of an iridium complex (80 µL of **X** mM stock solution in C_6D_6, **Y** mmol; **1 mol%**: X = 12.5, Y = 1.0 × 10^{-3}; **0.25 mol%**: X = 3.1, Y = 2.5 × 10^{-4}; **0.05 mol%**: X = 0.62, Y = 5.0 × 10^{-5}; **0.025 mol%**: X = 0.31, Y = 2.5 × 10^{-5}; **0.01 mol%**: X = 0.125, Y = 1.0 × 10^{-5}) was transferred to a J. Young tube. Pinacolborane solution (200 µL of 1.0 M pinacolborane stock solution in C_6D_6, 0.20 mmol) was added to the J. Young tube. 4-ethynyltoluene/1,4-dioxane (200 µL of 0.50 M 4-ethynyltoluene / 0.35 M 1,4-dioxane stock solution in C_6D_6, 0.10 mmol for 4-ethynyltoluene and 0.070 mmol for 1,4-dioxane) was then added in 4 portions with 1 min intervals. The J. Young tube was taken outside the glovebox, and

the reaction yield was determined by ^{1}H NMR spectroscopy by using 1,4-dioxane as an internal standard. The reagent ratio was calibrated through the integration ratio from a ^{1}H NMR spectrum of a sample obtained by mixing 100 µL of the pinacolborane stock solution and 100 µL 4-ethynyltoluene/1,4-dioxane stock solution in 0.3 mL C_6D_6 in another J. Young tube without adding other reagents.

General procedure for the NMR-scale DHBTA catalyzed by 17-Ir-COE (Chart 3) In an Ar-filled glove box, stock solution of **17-Ir-COE** (80 µL of **X** mM stock solution in C_6D_6, **Y** mmol; **0.1 mol%**: X = 1.25, Y = 1.0 × 10^{-4}; **0.025 mol%**: X = 0.31, Y = 2.5 × 10^{-5}) was transferred to a J. Young tube. Pinacolborane stock solution (200 µL of 1.0 M pinacolborane stock solution in C_6D_6, 0.20 mmol) was added to the J. Young tube. Alkyne/1,4-dioxane (200 µL of 0.50 M alkyne / 0.35 M 1,4-dioxane stock solution in C_6D_6, 0.10 mmol for alkyne and 0.070 mmol for 1,4-dioxane) was then added in 4 portions with 1 min intervals. The J. Young tube was taken outside the glovebox, and the reaction yield was determined by ^{1}H NMR spectroscopy using 1,4-dioxane as an internal standard. The reagent ratio was calibrated through the integration ratio from an ^{1}H NMR spectrum of a sample obtained by mixing 100 µL of the pinacolborane stock solution and 100 µL alkyne/1,4-dioxane stock solution in 0.3 mL C_6D_6 in another J. Young tube without adding other reagents. The ^{1}H NMR spectral data of alkynylboronates were in agreement with those reported in the literature.[1] **NMR-scale DHBTA catalyzed by 1-Ir-COE for comparison (For A6-H and A8-H in Chart 3)** In an Ar-filled glove box, stock solution of **1-Ir-COE** (80 µL of 0.0125 M stock solution in C_6D_6, 0.0010 mmol) and pinacolborane stock solution (200 µL of 1.0 M pinacolborane stock solution in C_6D_6, 0.20 mmol) were mixed in a J. Young tube. Alkyne/1,4-dioxane (200 µL of 0.50 M alkyne/ 0.35 M 1,4-dioxane stock solution in C_6D_6, 0.10 mmol for alkyne and 0.070 mmol for 1,4-dioxane)

was then added in 4 portions with 1 min intervals. The J. Young tube was taken outside the glovebox, after 10 min, reaction yield was determined on ^{1}H NMR measurement by using 1,4-dioxane as internal standard. The reagent ratio was calibrated through the integration ratio from a ^{1}H NMR spectrum of a sample obtained by mixing 100 µL of the pinacolborane stock solution and 100 µL alkyne/1,4-dioxane stock solution in 0.3 mL C_6D_6 in another J. Young tube without adding **1-Ir-COE**. **A6-H**: 9% yield of **A6-Bpin**. **A8-H**: <5% yield of **A8-Bpin**.

Me_3Si-O-CH_2C≡C-Bpin (A6-Bpin)

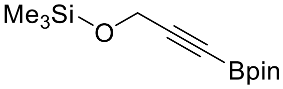

^{1}H NMR (500 MHz, C_6D_6): δ 4.08 (s, 2H, O-CH_2-C≡C), 0.96 (s, 12H, -CH_3 on Bpin), 0.72 (s, 9H, -CH_3 on TMS). ^{13}C{^{1}H} NMR (126 MHz, C_6D_6): δ 101.8 (br, <u>*C*</u>≡C-B), 84.0 (s, C_{quart}, Bpin), 51.5 (s, O-*C*H_2-C≡C), 24.7 (s, *C*H_3, Bpin), -0.22 (s, -*C*H_3 on TMS). ^{11}B NMR (128 MHz, C_6D_6): δ 24.0.

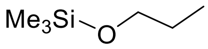

Me_3Si-O-$CH_2CH_2CH_3$ (A6-1)

^{1}H NMR (500 MHz, C_6D_6): δ 3.46 (t, J_{H-H} = 6.5 Hz, 2H, O-CH_2), 1.49 (m, 2H, O-CH_2-CH_2), 0.85 (t, J_{H-H} = 7.5 Hz, 3H, O-CH_2-CH_2-CH_3), 0.08 (s, 9H, -CH_3 on TMS).

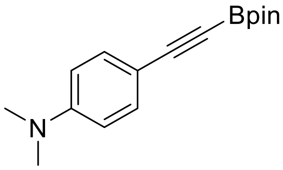

4-Me_2N-C_6H_4CCBpin (A7-Bpin)

^{1}H NMR (C_6D_6): δ 7.46 (d, J_{H-H} = 9 Hz, 2H, Ar-*H*), 6.18 (d, J_{H-H} = 9 Hz, 2H, Ar-*H*), 2.27 (s, 6H, N*Me*$_2$), 1.05 (s, 12H, CH_3 on Bpin); ^{13}C{^{1}H} NMR (C_6D_6): δ 150.8, 134.2, 111.9, 109.5,

104.1 (br, *C*≡C-B), 83.7 (s, C_{quart}, Bpin), 39.5 (s, N*Me*$_2$), 24.8 (s, *C*H_3, Bpin); ^{11}B (128 MHz, C_6D_6): δ 24.6.

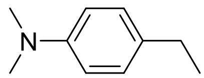
4-Me_2N-$C_6H_4CH_2CH_3$ (A7-1)

^{1}H NMR (C_6D_6): δ 7.07 (d, J_{H-H} = 9 Hz, 2H, Ar-*H*), 6.63 (d, J_{H-H} = 9 Hz, 2H, Ar-*H*), 2.56 (s, 6H, N*Me*$_2$), 2.52 (q, J_{H-H} = 7.5 Hz, 2H, C*H*$_2$CH$_3$), 1.17 (t, J_{H-H} = 7.5 Hz, 3H, CH$_2$C*H*$_3$).

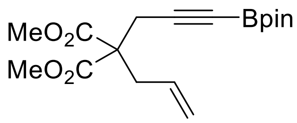

(MeO_2C)$_2$C(allyl)(CH_2CCBpin) (A9-Bpin)

^{1}H NMR (500 MHz, C_6D_6): δ 5.60 (ddt, J_{H-H} = 17 Hz, J_{H-H} = 10.5 Hz, J_{H-H} = 7.5 Hz, 1H, C*H*=CH$_2$), 5.09 (ddt, J_{H-H} = 17 Hz, J_{H-H} = 2 Hz, J_{H-H} = 1 Hz, 1H, CH=C*H*$_2$), 4.92 (ddt, J_{H-H} = 10.5 Hz, J_{H-H} = 2 Hz, J_{H-H} = 1 Hz, 1H, CH=C*H*$_2$), 3.25 (s, 6H, CO$_2$*Me*), 3.07 (s, 2H, C*H*$_2$CCBpin), 3.06 (dt, J_{H-H} = 7.5 Hz, J_{H-H} = 1.5 Hz, 2H, C*H*$_2$CH=CH$_2$), 0.94 (s, 12H, C*H*$_3$ on Bpin); ^{13}C NMR (126 MHz, C_6D_6): δ 169.9 (s, *C*O$_2$Me), 132.2 (s, *C*H=CH$_2$), 119.9 (s, CH=*C*H$_2$), 98.5 (s, *C*≡C-B), 84.0 (s, C_{quart}, Bpin), 57.3 (s, *C*(CO$_2$Me)$_2$), 52.3 (s, CO$_2$*Me*), 37.2 (s, *C*H$_2$), 24.5 (s, *C*H$_3$, Bpin), 24.3 (s, *C*H$_2$). ^{11}B (128 MHz, C_6D_6): δ 23.6.

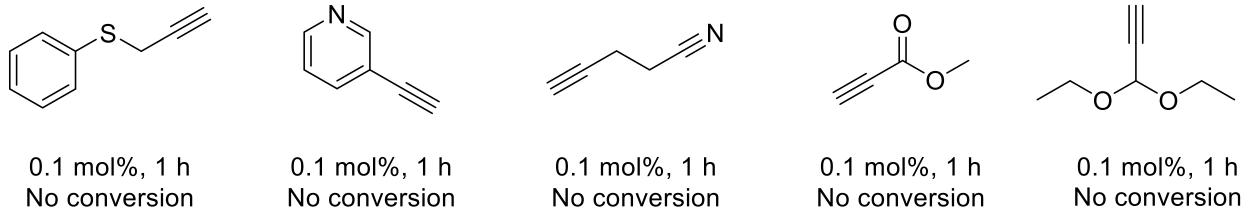

General procedure for NMR-scale DHBTA catalyzed by 17-Ir-COE using 1.1 eq. HBpin

In an Ar-filled glove box, a stock solution of **17-Ir-COE** (80 μL of 1.25 mM in C_6D_6, 1.0 × 10^{-4} mmol) was transferred to a J. Young tube. Pinacolborane/1,4-dioxane (110 μL of 1.0 M

pinacolborane / 0.35 M 1,4-dioxane stock solution in C_6D_6, 0.11 mmol for HBpin and 0.039 mmol for 1,4-dioxane) and C_6D_6 (90 µL) was added to the J. Young tube. Alkyne (200 µL of 0.50 M stock solution in C_6D_6, 0.10 mmol alkyne) was then added in 4 portions with 1 min intervals. The J. Young tube was taken outside the glovebox and heated at 60 °C for 1 h, and the reaction yield was determined by ^{1}H NMR spectroscopy using 1,4-dioxane as an internal standard. The reagent ratio was calibrated through the integration ratio from a ^{1}H NMR spectrum of a sample obtained by mixing 55 µL of the pinacolborane/1,4-dioxane stock solution with 100 µL of the alkyne stock solution in 0.35 mL C_6D_6 in another J. Young tube without adding **17-Ir-COE**.

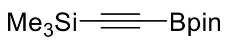

A2-Bpin
0.1 mol%, 1 h, 1.1 eq. HBpin
97%

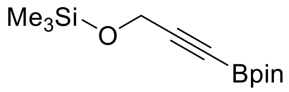

A6-Bpin
0.1 mol%, 1 h, 1.1 eq. HBpin
73%
18% Me_3SiO-$CH_2CH_2CH_3$ **A6-1**

Procedure for the preparative-scale DHBTA catalyzed by 17-Ir-COE (Chart 3) In an Ar-filled glove box, **17-Ir-COE** (200 µL of 0.0125 M stock solution in C_6D_6, 0.0025 mmol) and pinacolborane (2.90 mL, 20.0 mmol) were dissolved in 15 mL of specified solvent (toluene for **A 1–H** and **A5-H**, fluorobenzene for **A8-H**) to form a light yellow solution in a 100 mL PTFE-valved gas-tight flask. After stirring for 3 min at ambient temperature, alkyne (10.0 mmol) was then added dropwise in 30 s. Solution color remained light yellow and bubbles evolved slowly which indicated H_2 generation. After all alkyne was added, the flask was taken out glovebox and heated to 60 °C. After 1 h (5 h for **A8-H**), the flask was allowed to cool to ambient temperature and taken into the glove box. The solution was transferred to a 100 mL Schlenk flask and all volatiles were removed *in vacuo*. The crude product was redissolved in specified solvent

(pentane for **1–H** and **A5-H**, THF for **A8-H**) and placed in a -35 °C freezer for overnight. The next day the solid was collected, washed with cold pentane, and dried *in vacuo*. The decanted solution was combined with the washings, and the volatiles were removed *in vacuo*. The residue was then redissolved in specified solvent (pentane for **1–H** and **A5-H**, THF for **A8-H**), placed in the freezer and the 2nd fraction was collected in the same manner.

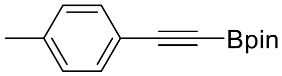

A1-Bpin

White solid, yield: 1.70 g (70%).

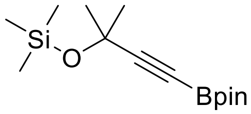

A5-Bpin

White solid, yield: 2.25 g (80%).

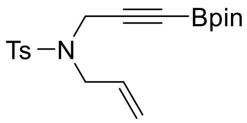

A8-Bpin

Light brown solid, yield: 3.09 g (82%). ^{1}H NMR (500 MHz, $CDCl_3$): δ 7.72 (d, J_{H-H} = 8.0 Hz, 2H, Ar-*H*), 7.27 (d, *J* = 7.5 Hz, 2 H, Ar-*H*), 5.69 (ddt, J_{H-H} = 17 Hz, J_{H-H} = 10 Hz, J_{H-H} = 6.5 Hz, 1H, C*H*=CH_2), 5.27 (apparent dq (ddt), J_{H-H} = 17 Hz, J_{H-H} = 1.5 Hz, 1H, CH=CH_2), 5.21 (apparent dq (ddt), J_{H-H} = 10.5 Hz, J_{H-H} = 1.5 Hz, 1H, CH=CH_2), 4.10 (s, 2H, CH_2-C≡C), 3.80 (d, J_{H-H} = 6 Hz, 2H, CH_2-CH=CH_2), 2.39 (s, 3H, Ar-*Me*), 1.21 (s, 12H, CH_3 on Bpin); ^{13}C{^{1}H} NMR (126 MHz, $CDCl_3$): δ 143.6 (s, *Ar*), 135.8 (s, *Ar*-Me), 131.9 (s, NCH_2*C*H=CH_2), 129.7 (s, *Ar*), 128.0 (s, *Ar*), 120.3 (NCH_2CH=*C*H_2), 95.3 (s, *C*≡C-B), 84.4 (s, C_{quart}, Bpin) 49.2 (s,

N*C*H_2CH=CH_2), 36.5 (s, N*C*H_2C≡CBpin), 24.7 (s, *C*H_3, Bpin), 21.7 (s, Ar-*Me*). $^{11}B\{^1H\}$ NMR (128 MHz, $CDCl_3$): δ 23.1.

VI. Synthesis of Proposed Intermediates in DHBTA.

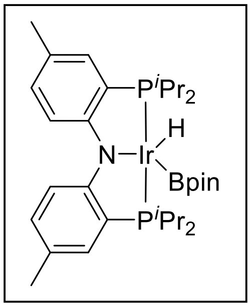

Synthesis of 10-Ir-HBpin. In a Ar-filled glove box, **10-Ir-H$_2$** (505 mg, 0.811 mmol) was dissolved in 5 mL fluorobenzene in a 25 mL Schlenk flask and pinacolborane (140 µL, 0.965 mmol) was added to it. The solution color changed from orange to yellow immediately. After 5 min, all volatiles were removed *in vacuo* to result in a yellow, sticky, foam-like solid. The residue was redissolved in 1 mL pentane and all volatiles were removed *in vacuo*. The residue was dissolved in 1 mL hexamethyldisiloxane then placed in a -35 °C freezer for 2 d. Then the supernatant solution was decanted and the remaining yellow solid was dried *in vacuo*. (Note: The synthesis on a smaller scale resulted in difficulty with isolation of the product due to its high solubility in hexamethyldisiloxane.) Its ^{1}H NMR spectroscopic analysis indicated >97% purity. Yield: 321 mg (53%). ^{1}H NMR (500 MHz, C_6D_6): δ 7.79 (d, $J_{H\text{-}H}$ = 8.5 Hz, 2H, Ar-*H*), 7.05 (s, 2H, Ar-*H*), 6.86 (d, $J_{H\text{-}H}$ = 8.5 Hz, 2H, Ar-*H*), 3.11 (m, 2H, C*H*Me$_2$), 2.27 (m, 2H, C*H*Me$_2$), 2.22 (s, 6H, Ar-C*H$_3$*), 1.34 (dvt, J = 14, 7.0 Hz, 6H, CH*Me$_2$*), 1.28 (dvt, J = 14, 7.0 Hz, 6H, CH*Me$_2$*), 1.24 (dvt, J = 14, 7.0 Hz, 6H, CH*Me$_2$*), 1.12 (s, 12H, C*H$_3$* on Bpin), 1.03 (dvt, J = 14, 7.0 Hz, 6H, CH*Me$_2$*), -19.82 (t, $J_{P\text{-}H}$ = 8.4 Hz, 1H, Ir-*H*). ^{13}C{^{1}H} NMR (126 MHz, C_6D_6): δ 164.4 (t, $J_{P\text{-}C}$ = 11 Hz), 133.1, 131.7, 126.0 (t, $J_{P\text{-}C}$ = 3.1 Hz), 124.3 (t, $J_{P\text{-}C}$ = 20 Hz), 115.8 (t, $J_{P\text{-}C}$ = 5.3 Hz), 81.2 (C_{quart}, Bpin), 24.7 (CH_3, Bpin), 24.5 (t, $J_{P\text{-}C}$ = 15 Hz), 24.0 (t, $J_{P\text{-}C}$ = 15 Hz), 20.1, 19.5 (t, $J_{P\text{-}C}$ = 3.0 Hz), 18.8, 18.7 (t, $J_{P\text{-}C}$ = 2.2 Hz), 16.4. ^{31}P{^{1}H} NMR (121 MHz, C_6D_6): δ 51.8. ^{11}B{^{1}H} NMR (128 MHz, C_6D_6): δ 26.4. Anal. Calcd for $C_{32}H_{53}BIrNO_2P_2$: C, 51.33; H, 7.14. Found: C, 51.24; H, 7.31.

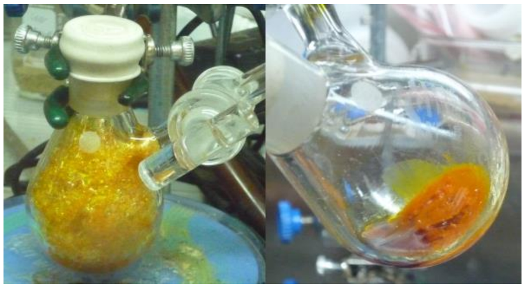

Figure S1. Left: Foam-like **10-Ir-HBpin**; Right: **10-Ir-HBpin** after recrystallization from hexamethyldisiloxane.

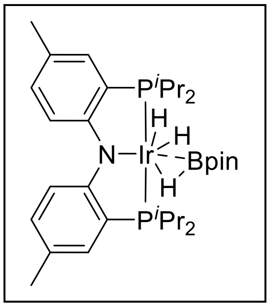

Observation of 10-Ir-H_3Bpin. In a Ar-filled glove box, **10-Ir-HBpin** (20 mg, 0.027 mmol) was dissolved in 0.5 mL C_6D_6 (toluene-d_8 was used instead for the variable-temperature NMR analysis in an otherwise analogous experiment) in a J. Young tube. The tube was taken out and degassed by three freeze-pump-thaw cycles and back-filled with H_2 (1 atm, excess). After 10 min at ambient temperature, the solution was examined by NMR spectroscopy. ^{1}H NMR (400 MHz, C_6D_6): δ 7.78 (d, $J_{H\text{-}H}$ = 8.5 Hz, 2H, Ar-*H*), 6.98 (s, 2H, Ar-*H*), 6.77 (d, $J_{H\text{-}H}$ = 8.5 Hz, 2H, Ar-*H*), 2.81 (m, 2H, C*H*Me$_2$), 2.22 (s, 6H, Ar-C*H$_3$*), 2.00 (m, 2H, C*H*Me$_2$), 1.31 (dvt, J = 14, 7.0 Hz, 6H, CH*Me$_2$*), 1.23 (dvt, J = 14, 7.0 Hz, 6H, CH*Me$_2$*), 1.17 (dvt, J = 14, 7.0 Hz, 6H, CH*Me$_2$*), 1.07 (s, 12H, C*H$_3$* on Bpin), 1.04 (dvt, J = 14, 7.0 Hz, 6H, -CH*Me$_2$*), -5.35 (br s, 1H, $\omega_{1/2}$ = 60 Hz), -12.40 (br s, 2H, $\omega_{1/2}$ = 64 Hz). Selected ^{1}H{^{11}B} NMR data (400 MHz, C_6D_6): -5.35 (br s, 1H, $\omega_{1/2}$ = 35 Hz), -12.40 (br s, 2H, $\omega_{1/2}$ = 64 Hz). Selected ^{1}H NMR data (293 K, 500 MHz, toluene-d_8): -5.43 (br s, 1H), -12.51 (br s, 2H). Selected ^{1}H NMR data (213 K, 500 MHz, toluene-d_8): -5.39 (br s, 1H), -9.43 (br s, 1H), -15.35 (br s, 1H). ^{31}P{^{1}H} NMR (202 MHz, C_6D_6): δ 40.8. ^{11}B{^{1}H} NMR (128 MHz, C_6D_6): δ 32.9.

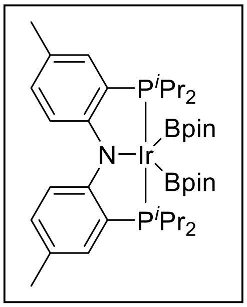

Synthesis of 10-Ir-Bpin$_2$. In an Ar-filled glove box, **10-Ir-HMes** (133 mg, 0.180 mmol) and B_2pin_2 (46 mg, 0.18 mmol) were transferred to a 10 mL PTFE-valved gas-tight flask and dissolved in 0.5 mL mesitylene. The flask was taken outside the glovebox and heated at 100 °C for 1 h. The solution color turned from brown to yellow gradually and yellow crystals formed. After allowing the mixture to cool to ambient temperature, the flask was taken into a glovebox. Et_2O was added to the flask to assist with transferring the solution to a 25 mL Schlenk flask, and then the volatiles were removed *in vacuo*. The residue was redissolved in CH_2Cl_2/pentane mixture, and the flask was then placed in a -35 °C freezer overnight. The next day the solid was collected by decantation, washed with cold pentane and dried under vacuum resulting in a yellow solid. The decanted solution was combined with the washings, and the volatiles were removed *in vacuo*. The residue was then redissolved in CH_2Cl_2/pentane, placed in the freezer and the 2nd fraction was collected in the same manner. Its ^{1}H NMR spectroscopic analysis indicated >98% purity. Combined yield: 130 mg (83%). ^{1}H NMR (500 MHz, C_6D_6): δ 7.57 (d, J_{H-H} = 8.5 Hz, 2H, Ar-*H*), 6.96 (s, 2H, Ar-*H*), 6.84 (d, J_{H-H} = 8.5 Hz, 2H, Ar-*H*), 2.93 (m, 4H, C*H*Me$_2$), 2.23 (s, 6H, Ar-C*H$_3$*), 1.48 (dvt, J = 14, 7.0 Hz, 12H, CH*Me$_2$*), 1.19 (dvt, J = 14, 7.0 Hz, 12H, CH*Me$_2$*), 1.18 (s, 24H, C*H$_3$* on Bpin). ^{13}C{^{1}H} NMR (126 MHz, $CDCl_3$): δ 161.9 (t, J_{P-C} = 9.2 Hz), 132.8, 130.8, 125.2 (t, J_{P-C} = 3.5 Hz), 124.2 (t, J_{P-C} = 20 Hz), 114.6 (t, J_{P-C} = 4.5 Hz), 81.6 (*C*$_{quart}$, Bpin), 25.7 (*C*H$_3$, Bpin), 23.8 (br), 20.6, 19.0 (br), 17.0. ^{31}P{^{1}H} NMR (202 MHz, C_6D_6): δ 46.4. ^{11}B{^{1}H} NMR (128 MHz, $CDCl_3$): δ 28.5. Anal. Calcd for $C_{38}H_{64}B_2IrNO_4P_2$: C, 52.18; H, 7.38. Found: C, 52.28; H, 7.24.

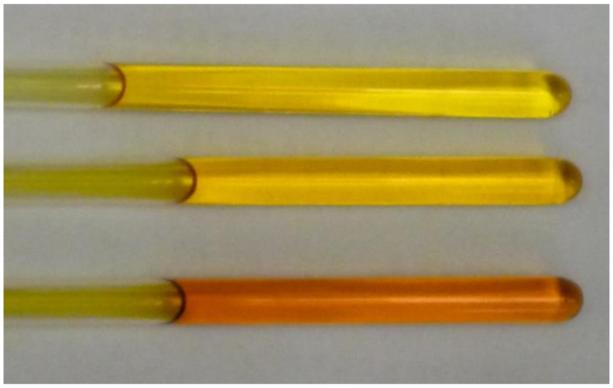

Figure S2. From top to bottom: **10-Ir-H$_3$Bpin**, **10-Ir-Bpin$_2$**, **10-Ir-HBpin** in C_6D_6 (same concentration).

Attempt at making terminal alkyne derived complexes (1). 10-Ir-H$_2$ (14 mg, 0.023 mmol) was dissolved in 0.5 mL PhF in a 10 mL Schlenk flask and followed by **A1-H** (226 μL of 0.1 M stock solution in C_6D_6, 0.0226 mmol). After 10 s at RT, all volatiles were removed *in vacuo*. The residue was redissolved in C_6D_6 and analysis by ^{31}P NMR spectroscopy revealed 36% **10-Ir-H$_2$** and 64% unidentified compounds: 6% unknown at δ 40.3, 32% unknown at δ 29.7, 26% unknown at δ 28.6.

Attempt at making terminal alkyne derived complexes (2). 10-Ir-H$_2$ (10 mg, 0.018 mmol) was dissolved in 0.5 mL C_6D_6 in a J. Young tube and followed by **A1-H** (11 μL, 0.087 mmol). After 10 min at RT, analysis by ^{31}P spectroscopy revealed 36% unknown at δ 37.5, 36%unknown at δ 30.4, 28% unknown at δ 28.3.

Attempt at making terminal alkyne derived complexes (3). 10-Ir-HMes (12 mg, 0.016 mmol) was dissolved in 0.5 mL mesitylene in a J. Young tube and followed by **A1-H** (32 μL of 0.5 M stock solution in mesitylene, 0.016 mmol). After 1 d at RT, analysis by ^{31}P spectroscopy revealed 58% **10-Ir-HMes** and 42% unidentified compounds: 6% unknown at δ 48.4, 5% unknown at δ 37.3, 16% unknown at δ 29.9, 4% unknown at δ 29.1, and 11% unknown at δ 28.2.

Observation of 10-Ir-v-tol and 10-Ir-p-tol. In a Ar-filled glove box, **10-Ir-HMes** (15 mg, 0.021 mmol) and **A1-Bpin** (210 µL of 0.1 M stock solution in mesitylene, 0.021 mmol) were dissolved in 300 µL mesitylene in a J. Young tube. After overnight, ^{31}P NMR spectroscopic analysis revealed 84% 10-Ir-HMes, 5% **10-Ir-v-tol** and 11% **10-Ir-p-tol**.

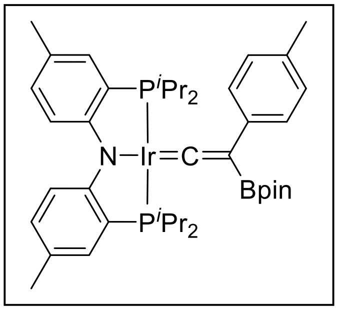

Synthesis of 10-Ir-v-tol. In a Ar-filled glove box, **10-Ir-HMes** (118 mg, 0.160 mmol) and **A1-Bpin** (39 mg, 0.16 mmol) were dissolved in 1.0 mL mesitylene in a J. Young tube. The tube was taken out of the glovebox and then placed in a 100 °C oil bath for 1 h. The J. Young tube was allowed to cool to ambient temperature and brought back into the glovebox. The solution was transferred to a 25 mL Schlenk flask with fluorobenzene to assist. All volatiles were removed *in vacuo*. The residue was redissolved in PhF/pentane, and the flask was then placed in a -35 °C freezer overnight. The next day, the solid was collected by decantation, washed with cold pentane, and dried under vacuum to produce a brick-red solid. The decanted solution was combined with the washings, and the volatiles were removed *in vacuo*. The residue was then redissolved in PhF/pentane, placed in the freezer and the second fraction was collected in the same manner. Its ^{1}H NMR spectroscopic analysis indicated >97% purity. Combined yield: 70 mg (51%). ^{1}H NMR (500 MHz, C_6D_6): δ 8.19 (d, J = 8.5 Hz, 2H, Ar-*H*), 7.67 (d, J_{H-H} = 8.5 Hz, 2H, Ar-*H*), 7.15 (d, J_{H-H} = 8.5 Hz, 2H, Ar-*H*, overlapping with the solvent peak), 7.06 (s, 2H, Ar-*H*), 6.83 (d, J_{H-H} = 8.5 Hz, 2H, Ar-*H*), 2.58 (m, 4H, C*H*Me$_2$), 2.21 (s, 6H, Ar-CH$_3$), 1.38 (m, 12H, CH*Me$_2$*), 1.18 (m, 24H, CH*Me$_2$* and C*H$_3$* on Bpin). ^{13}C{^{1}H} NMR (126 MHz, C_6D_6): δ 282.8 (t, J_{P-C} = 10 Hz, Ir=*C*), 163.4 (t, J_{P-C} = 11 Hz), 133.0, 132.6, 131.8, 129.0, 127.0 (t, J_{P-C} = 3.0 Hz), 126.8, 124.8 (t, J_{P-C} = 21 Hz), 120.9, 116.6 (t, J_{P-C} = 7.0 Hz), 82.0 (*C*$_{quart}$, Bpin), 26.4 (t, J_{P-C} = 15 Hz), 25.1 (*C*H$_3$, Bpin), 21.1, 20.4,

19.4, 18.5. (Note: In ^{13}C NMR spectra, the quaternary carbon atom attached to boron was not observed.) $^{31}P\{^1H\}$ NMR (202 MHz, C_6D_6): δ 43.9. $^{11}B\{^1H\}$ NMR (128 MHz, C_6D_6): δ 22.5. Anal. Calcd for $C_{41}H_{59}BIrNO_2P_2$: C, 57.07; H, 6.89. Found: C, 57.18; H, 6.89.

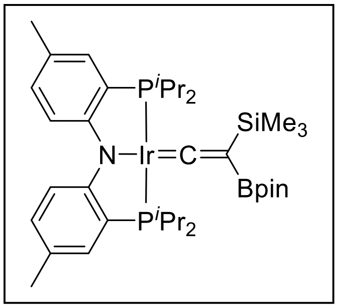

Synthesis of 10-Ir-v-TMS. In a Ar-filled glove box, **10-Ir-HMes** (52 mg, 0.070 mmol) and **A2-Bpin** (16 mg, 0.070 mmol) were dissolved in 0.5 mL mesitylene in a J. Young tube. The tube was left at RT overnight. ^{31}P NMR spectroscopic analysis revealed 40% of **10-Ir-HMes** and 60% of **10-Ir-v-TMS**. The tube was then placed in 100 °C oil bath for 1 h. ^{31}P NMR spectroscopic analysis revealed >90% conversion to **10-Ir-v-TMS**. The tube was brought into a glovebox and the solution was transferred to a 25 mL Schlenk flask. All volatiles were removed *in vacuo* to yield a red oil, and attempts at recrystallization of this residue failed. The residue was taken up in C_6D_6 for NMR analysis. ^1H NMR (500 MHz, C_6D_6): δ 7.66 (d, $J_{H\text{-}H}$ = 8.5 Hz, 2H, Ar-*H*), 7.04 (s, 2H, Ar-*H*), 6.81 (d, $J_{H\text{-}H}$ = 8.5 Hz, 2H, Ar-*H*), 2.70 (m, 2H, C*H*Me$_2$), 2.60 (m, 2H, C*H*Me$_2$), 2.22 (s, 6H, Ar-C*H$_3$*), 1.48 (dvt, J = 14, 7.0 Hz, 6H, CH*Me$_2$*), 1.42 (dvt, J = 14, 7.0 Hz, 6H, CH*Me$_2$*), 1.27 (dvt, J = 14, 7.0 Hz, 6H, CH*Me$_2$*), 1.18 (dvt, J = 14, 7.0 Hz, 6H, CH*Me$_2$*), 1.10 (s, 12H, C*H$_3$* on Bpin), 0.49 (s, 9H, C*H$_3$* on TMS). $^{13}C\{^1H\}$ NMR (126 MHz, C_6D_6): δ 269.3 (t, $J_{P\text{-}C}$ = 8.8 Hz, Ir=*C*), 163.8 (t, $J_{P\text{-}C}$ = 11 Hz), 132.9, 131.8, 126.0 (t, $J_{P\text{-}C}$ = 3.3 Hz), 124.4 (t, $J_{P\text{-}C}$ = 21 Hz), 116.3 (t, $J_{P\text{-}C}$ = 5.1 Hz), 81.7 (C_{quart}, Bpin), 26.7 (t, $J_{P\text{-}C}$ = 15 Hz), 26.6 (t, $J_{P\text{-}C}$ = 15 Hz), 24.9 (*C*H$_3$, Bpin), 20.5, 19.7, 19.6, 18.6, 18.5, 1.2 (Si*Me$_3$*). (Note: In ^{13}C NMR spectra, the quaternary carbon atom attached to boron was not observed.) $^{31}P\{^1H\}$ NMR (202 MHz, C_6D_6): δ 44.0. $^{11}B\{^1H\}$ NMR (128 MHz, C_6D_6): δ 21.6.

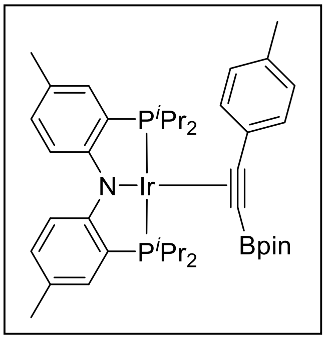

Synthesis of 10-Ir-p-tol. In an Ar-filled glove box, **10-Ir-H$_2$** (34 mg, 0.054 mmol) was dissolved in 1 mL flurobenzene in a 25 mL Schlenk flask and stock solution of **A1-Bpin** (540 µL of 0.1 M stock solution in C_6D_6, 0.054 mmol) was added to the solution, and the solution color turned red-orange immediately. After 10 s, all volatiles were removed *in vacuo*. The residue was taken up in C_6D_6 for NMR analysis. ^{31}P NMR spectroscopic analysis revealed 85% **10-Ir-p-tol**, 9% **10-Ir-HBpin** and 6% unidentified species. ^{1}H NMR (300 MHz, C_6D_6): δ 8.28 (d, $J_{H\text{-}H}$ = 8.1 Hz, 2H, Ar-*H*), 7.76 (d, $J_{H\text{-}H}$ = 8.1 Hz, 2H, Ar-*H*), 6.99 (d, $J_{H\text{-}H}$ = 8.1 Hz, 2H, Ar-*H*), 6.90 (s, 2H, Ar-*H*), 6.86 (d, $J_{H\text{-}H}$ = 8.1 Hz, 2H, Ar-*H*), 2.60 (m, 2H, CH*Me$_2$*), 2.20 (m, 8H, Ar-C*H$_3$* of PNP and CH*Me$_2$*), 2.00 (s, 3H, Ar-C*H$_3$* of alkynylboronate), 1.41 (dvt, J = 14, 7.0 Hz, 6H, CH*Me$_2$*), 1.20 (m, 24H, C*H$_3$* on Bpin and -CH*Me$_2$*), 0.94 (dvt, J = 14, 7.0 Hz, 6H, CH*Me$_2$*). ^{31}P{^{1}H} NMR (121 MHz, C_6D_6): δ 25.6.

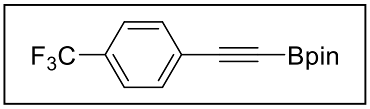

Synthesis of 4-$CF_3C_6H_4C{\equiv}CBpin$ (A10-Bpin). The procedure was adapted from the previously reported synthesis.[1] In an Ar-filled glove box, **1-Ir-COE** (14 mg, 0.020 mmol) and pinacolborane (580 µL, 4.00 mmol) were dissolved in 4 mL PhF in a 50 mL Schlenk flask. After stirring for 3 min at ambient temperature, 4-$CF_3C_6H_4C{\equiv}CH$ (**A10-H**, 326 µL, 2.00 mmol) in 3 mL PhF was then added dropwise over 5 min. Bubbles evolved immediately which indicated H_2 generation. After all alkyne was added, the mixture was stirred for 5 min and then the volatiles were removed *in vacuo*. The residue was recrystallized in PhF/pentane in a -35 °C freezer. After overnight, the solution was decanted and the solid was washed with 1 mL pentane three times. The solid was dried *in vacuo* to yield off-white crystals. Its ^{1}H NMR spectroscopic analysis indicated >98% purity. Yield: 441 mg (74%). ^{1}H NMR (500 MHz, C_6D_6): δ 7.11 (d, $J_{H\text{-}H}$ = 8.5 Hz, 2H, Ar-*H*), 6.97 (d, $J_{H\text{-}H}$ = 8.5 Hz, 2H, Ar-

H), 1.02 (s, 12H, -CH_3 on Bpin). ^{13}C{^{1}H} NMR (126 MHz, C_6D_6): δ 132.8, 130.8 (q, $J_{F\text{-}C}$ = 33 Hz), 126.1, 125.4 (q, $J_{F\text{-}C}$ = 3.8 Hz), 124.4 (q, $J_{F\text{-}C}$ = 270 Hz, *C*F$_3$), 100.0 (br, **C**≡C-B), 84.4 (*C*$_{quart}$, Bpin), 24.7 (*C*H$_3$, Bpin). ^{19}F NMR (470 MHz, C_6D_6): δ -63.7. ^{11}B{^{1}H} NMR (128 MHz, C_6D_6): δ 24.4.

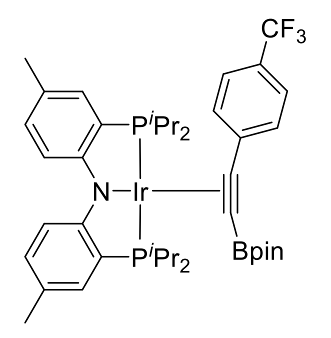

Synthesis of 10-Ir-p-F$_3$tol. In an Ar-filled glove box, **10-Ir-H$_2$** (204 mg, 0.327 mmol) was dissolved in 2 mL fluorobenzene in a 25 mL Schlenk flask. The flask was placed in a -35 °C freezer for 2 h. **A10-Bpin** (97 mg, 0.33 mmol) was added to the solution, and the solution color turned red-orange immediately. After 10 s, all volatiles were removed *in vacuo*. (Note: Longer reaction times would result in more **10-Ir-HBpin** formation.) 3 mL pentane was added to the flask and a spatula was used to dislodge the filmy solid off the walls of the flask to give an red-orange powder. The suspension was vigorously stirred for 10 min and then placed in a -35 °C freezer. After overnight, the solution was decanted and 3 mL pentane was added to the flask. The suspension was filtered through a fritted funnel.The solid was washed with pentane and dried under vacuum to get red-orange solid. Its ^{1}H NMR spectroscopic analysis indicated >98% purity. Yield: 221 mg (74%). ^{1}H NMR (500 MHz, C_6D_6): δ 8.22 (d, $J_{H\text{-}H}$ = 8.5 Hz, 2H, Ar-*H*), 7.71 (d, $J_{H\text{-}H}$ = 8.5 Hz, 2H, Ar-*H*), 7.39 (d, $J_{H\text{-}H}$ = 8.5 Hz, 2H, Ar-*H*), 6.87 (s, 2H, Ar-*H*), 6.84 (d, $J_{H\text{-}H}$ = 8.5 Hz, 2H, Ar-*H*), 2.54 (m, 2H, C*H*Me$_2$), 2.19 (s, 6H, Ar-CH_3), 2.08 (m, 2H, C*H*Me$_2$), 1.33 (dvt, J = 14, 7.0 Hz, 6H, CH*Me$_2$*), 1.19 (s, 12H, CH_3 on Bpin), 1.14 (dvt, J = 14, 7.0 Hz, 12H, CH*Me$_2$*), 0.87 (dvt, J = 14, 7.0 Hz, 6H, CH*Me$_2$*). ^{13}C{^{1}H} NMR (100 MHz, CD_2Cl_2): δ 164.4 (t, $J_{P\text{-}C}$ = 9.4 Hz), 132.6, 131.3, 131.0, 130.1, 127.6 (q, $J_{F\text{-}C}$ = 32 Hz), 126.7 (t, $J_{P\text{-}C}$ = 3.1 Hz), 125.4 (q, $J_{F\text{-}C}$ = 3.8 Hz), 125.2 (q, $J_{F\text{-}C}$ = 270 Hz, *C*F$_3$), 124.8 (t, $J_{P\text{-}C}$ = 20 Hz), 114.7 (t, $J_{P\text{-}C}$ = 4.8 Hz), 105.7 (**C**≡C-B), 83.5 (*C*$_{quart}$, Bpin), 25.0 (*C*H$_3$, Bpin), 24.1

(t, $J_{P\text{-}C}$ = 13 Hz), 23.5 (t, $J_{P\text{-}C}$ = 13 Hz), 20.3, 18.6, 18.4, 17.6, 17.1. $^{31}P\{^1H\}$ NMR (202 MHz, C_6D_6): δ 26.1. $^{11}B\{^1H\}$ NMR (128 MHz, C_6D_6): δ 25.5. ^{19}F NMR (470 MHz, C_6D_6): δ 62.9. Anal. Calcd for $C_{41}H_{56}BF_3IrNO_2P_2$: C, 53.71; H, 6.16. Found: C, 53.73; H, 6.29.

VII. Stoichiometric Reactions of 10-Ir Complexes.

Reaction of 10-Ir-HBpin with 4-$CH_3C_6H_4C{\equiv}CH$. 10-Ir-HBpin (14 mg, 0.019 mmol) and stock solution of 4-$CH_3C_6H_4C{\equiv}CH$ (190 µL of 0.1 M stock solution in C_6D_6, 0.019 mmol) was dissolved in 0.3 mL C_6D_6 in a J. Young tube. After 10 min at RT, analysis by ^{31}P NMR spectroscopy revealed ~50% **10-Ir-HBpin**, 16% unknown A (δ 48.8), 14% unknown B (δ 29.5), 14% unknown C (δ 28.7). ~50% Bpin groups were found in 4-$CH_3C_6H_4C{\equiv}CBpin$ based on analysis by ^{1}H NMR spectroscopy. The amount of 4-$CH_3C_6H_4C{\equiv}CBpin$ did not increase after a longer reaction time, and other side reactions such as hydrogenation occurred.

Reaction of 10-Ir-HBpin with 4-$CF_3C_6H_4C{\equiv}CH$. 10-Ir-HBpin (11 mg, 0.014 mmol) and stock solution of 4-$CF_3C_6H_4C{\equiv}CH$ (28 µL of 0.5 M stock solution in C_6D_6, 0.014 mmol) was dissolved in 0.4 mL C_6D_6 in a J. Young tube. After 10 min at RT, analysis by ^{31}P NMR spectroscopy revealed ~50% **10-Ir-HBpin**, 23% unknown A' (δ 49.2), 14% unknown B' (δ 28.9), 14% unknown C' (δ 28.5), and 3% **10-Ir-p-F_3tol**. ~50% Bpin groups were found in 4-$CF_3C_6H_4C{\equiv}CBpin$ based on analysis by ^{1}H NMR spectroscopy. The amount of 4-$CF_3C_6H_4C{\equiv}CBpin$ did not increase after a longer reaction time, and other side reactions such as hydrogenation occurred.

Reaction of 10-Ir-HBpin with $Me_3SiC{\equiv}CH$. 10-Ir-HBpin (11 mg, 0.014 mmol) and stock solution of trimethylsilylacetylene (28 µL of 0.5 M stock solution in C_6D_6, 0.014 mmol) was dissolved in 0.4 mL C_6D_6 in a J. Young tube. After 10 min at RT, analysis by ^{31}P NMR spectroscopy revealed 5% **10-Ir-HBpin**, 33% unknown A'' (δ 48.7), 44% unknown B'' (δ 44.6), 14% unknown C'' (δ 41.0), 6% unknown D'' (δ 39.3) and 3% unknown E'' (δ 34.5). ~50% Bpin groups were found in $Me_3Si\text{-}C{\equiv}C\text{-}Bpin$ based on analysis by ^{1}H NMR spectroscopy.

Reaction of 10-Ir-Bpin$_2$ with pinacolborane. 10-Ir-Bpin$_2$ (13 mg, 0.015 mmol) and stock solution of pinacolborane (15 µL of 1.0M stock solution in C_6D_6, 0.015 mmol) was dissolved in 0.5 mL C_6D_6 in a J. Young tube. The mixture was heated at 100 °C overnight, analysis by ^{1}H and ^{31}P NMR spectroscopy revealed that only unreacted **10-Ir-Bpin$_2$** and pinacolborane were present.

Reaction of 10-Ir-Bpin$_2$ with 4-$CH_3C_6H_4C{\equiv}CH$. 10-Ir-Bpin$_2$ (12 mg, 0.014 mmol) and stock solution of 4-ethynyltoluene (27 µL of 0.50M stock solution in C_6D_6, 0.014 mmol) was dissolved in 0.5 mL C_6D_6 in a J. Young tube. After 3 h at RT, analysis by ^{1}H and ^{31}P NMR spectroscopy revealed that only unreacted **10-Ir-Bpin$_2$** and 4-ethynyltoluene were present. The mixture was heated at 100 °C overnight, **10-Ir-Bpin$_2$** was still intact based on the analysis by ^{31}P NMR spectroscopy, but 4-ethynyltoluene was completely consumed to yield a mixture of products.

Reaction of 10-Ir-Bpin$_2$ with H_2. 10-Ir-Bpin$_2$ (10 mg, 0.012 mmol) was dissolved in 0.5 mL C_6D_6 in a J. Young tube. The tube was degassed by three freeze-pump-thaw cycles and back-filled with H_2 (1 atm, excess). After 3 h at RT, analysis by ^{1}H and ^{31}P NMR spectroscopy revealed that only unreacted **10-Ir-Bpin$_2$** and H_2 were present. Heating the mixture at 100 °C overnight resulted in the observation of 59% **10-Ir-Bpin$_2$** and 41% **10-Ir-H$_3$Bpin** by ^{31}P NMR spectroscopy.

Reaction of 10-Ir-v-tol with H_2. 10-Ir-v-tol (10 mg, 0.011 mmol) was dissolved in 0.5 mL C_6D_6 in a J. Young tube. The tube was degassed by three freeze-pump-thaw cycles and back-filled with H_2 (1 atm, excess). After 1 h at RT, 80% **10-Ir-v-tol** was converted to several unknown products (no observable signals in ^{31}P NMR) and 60% yield of $H_2C{=}C$(4-$CH_3C_6H_4$)(Bpin) (**A1-4**) based on the analysis by ^{1}H NMR spectroscopy. After overnight at

ambient temperature, **10-Ir-H$_2$** was the only observable species in ^{31}P NMR spectroscopy analysis. Selected NMR data for **A1-4**. ^{1}H NMR (500 MHz, C_6D_6): δ 6.39 (d, J = 3.0 Hz, 1H, alkenyl-*H*), 6.15 (d, J = 3.0 Hz, 1H, alkenyl-*H*), 1.05 (s, 12H, -CH_3 on Bpin).

Reaction of 10-Ir-v-tol with pinacolborane. 10-Ir-v-tol (12 mg, 0.014 mmol) and stock solution of pinacolborane (14 µL of 1.0M stock solution in C_6D_6, 0.014 mmol) was dissolved in 0.5 mL C_6D_6 in a J. Young tube. After 3 h at RT, analysis by ^{1}H and ^{31}P NMR spectroscopy revealed that only unreacted **10-Ir-v-tol** and pinacolborane were present. After heating the mixture for 1 h at 100 °C, <5% decomposition of **10-Ir-v-tol** was observed by ^{1}H NMR spectroscopy analysis and **10-Ir-v-tol** remained the only observable species in the ^{31}P NMR spectrum.

Reaction of 10-Ir-v-tol with 4-$CH_3C_6H_4C{\equiv}CH$. 10-Ir-v-tol (11 mg, 0.013 mmol) and stock solution of 4-ethynyltoluene (26 µL of 0.50M stock solution in C_6D_6, 0.013 mmol) were dissolved in 0.5 mL C_6D_6 in a J. Young tube. After 3 h at RT and 1 h at 100 °C, analysis by ^{1}H and ^{31}P NMR spectroscopy revealed that only unreacted **10-Ir-v-tol** and 4-ethynyltoluene were present. After 15 h at 100 °C, <5% decomposition of **10-Ir-v-tol** was observed by ^{1}H NMR spectroscopy analysis and **10-Ir-v-tol** remained the only observable species in the ^{31}P NMR spectrum.

Reaction of 10-Ir-p-F$_3$tol with H$_2$. 10-Ir-p-F$_3$tol (10 mg, 0.011 mmol) was dissolved in 0.5 mL C_6D_6 in a J. Young tube. The tube was degassed by three freeze-pump-thaw cycles and back-filled with H_2 (1 atm, excess). The solution color turned lighter orange gradually. After 10 min at RT, there were 49% **10-Ir-p-F$_3$tol**, 27% **10-Ir-H$_3$Bpin**, and 24% unknown (δ 31.1) based on the analysis by ^{31}P NMR spectroscopy.

Reaction of 10-Ir-p-F_3tol with pinacolborane. 10-Ir-p-F_3tol (11 mg, 0.011 mmol) and stock solution of pinacolborane (22 µL of 0.5 M stock solution in C_6D_6, 0.011 mmol) were dissolved in 0.5 mL C_6D_6 in a J. Young tube. After 10 min at RT, there was 14% **10-Ir-HBpin** and 86% **10-Ir-p-F_3tol** based on the analysis by ^{31}P NMR spectroscopy. After 4 h at RT, there was 83% **10-Ir-HBpin** and 17% **10-Ir-p-F_3tol** based on the analysis by ^{31}P NMR spectroscopy, and analysis by ^{1}H NMR spectroscopy revealed that only free 4-$CF_3C_6H_4C{\equiv}CBpin$, free pinacolborane, **10-Ir-HBpin**, **10-Ir-p-F_3tol** were present.

Reaction of 10-Ir-p-F_3tol with 4-$CF_3C_6H_4C{\equiv}CH$. 10-Ir-p-F_3tol (10 mg, 0.011 mmol) and stock solution of 4-$CF_3C_6H_4C{\equiv}CH$ (22 µL of 0.50M stock solution in C_6D_6, 0.011 mmol) was dissolved in 0.5 mL C_6D_6 in a J. Young tube. After 10 min at RT, analysis by ^{1}H and ^{31}P NMR spectroscopy revealed that only unreacted **10-Ir-p-F_3tol** and 4-ethynyltoluene were present. After 2 h at RT, analysis by ^{1}H NMR spectroscopy revealed 8% free 4-$CF_3C_6H_4C{\equiv}CBpin$ and 92% **10-Ir-p-F_3tol** were present as alkynylboronate related species.

General procedure for the NMR-scale dehydrogenative borylation of alkynes using various 10-Ir complexes (Chart 4)

In an Ar-filled glove box, stock solution of iridium catalyst (80 µL of 0.0125 M stock solution in C_6D_6, 0.0010 mmol) and pinacolborane stock solution (200 µL of 1.0 M pinacolborane stock solution in C_6D_6, 0.20 mmol) were mixed in a J. Young tube. Alkyne/1,4-dioxane (200 µL of 0.50 M alkyne/ 0.35 M 1,4-dioxane stock solution in C_6D_6, 0.10 mmol for alkyne and 0.070 mmol for 1,4-dioxane) was then added in 4 portions with 1 min intervals. The J. Young tube was taken outside the glovebox after 10 min, and the reaction yield was determined by ^{1}H NMR spectroscopy using 1,4-dioxane as an internal standard. The reagent ratio was calibrated through the integration ratio from a ^{1}H NMR spectrum of a sample obtained by mixing 100 µL of the pinacolborane stock solution and 100 µL alkyne/1,4-dioxane stock solution in 0.3 mL C_6D_6 in another J. Young tube without adding iridium catalyst.

Catalytic results for DHBTA using various (^{Me}PNP^{iPr})Ir complexes

Ir catalyst	Alkyne	DHBTA Yield (10 min)	Note
**10-Ir-H$_2$**	**A1-H**	81% (16% **A1-H**, 3% **A1-3**)	95% after 1 h (5% **A1-3**)
**10-Ir-HBpin**	**A1-H**	82% (16% **A1-H**, 2% **A1-3**)	96% after 1 h (4% **A1-3**)
**10-Ir-Bpin$_2$**	**A1-H**	0% (all **A1-H**)	37% after 3 h
**10-Ir-v-tol**	**A1-H**	17% (80% **A1-H**)	85% after 1 h
**10-Ir-H$_2$**	**A10-H**	45% (6% **A10-1**)	65% after 1 h (9% **A10-1**)
**10-Ir-p-F$_3$tol**	**A10-H**	49% (6% **A10-1**)	64% after 1 h (9% **A10-1**)

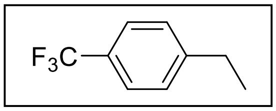

A10-1. ^{1}H NMR (500 MHz, C_6D_6): δ 7.32 (d, J_{H-H} = 8.0 Hz, 2H, Ar-*H*), 6.77 (d, J_{H-H} = 8.0 Hz, 2H, Ar-*H*), 2.23 (q, J_{H-H} = 7.6 Hz, 2H, Ar-C*H*$_2$CH$_3$), 2.14 (s, 3H, Ar-C*H*$_3$), 0.90 (t, J_{H-H} = 7.6 Hz, 3H, Ar-CH$_2$C*H*$_3$).

VIII. NMR Spectra.

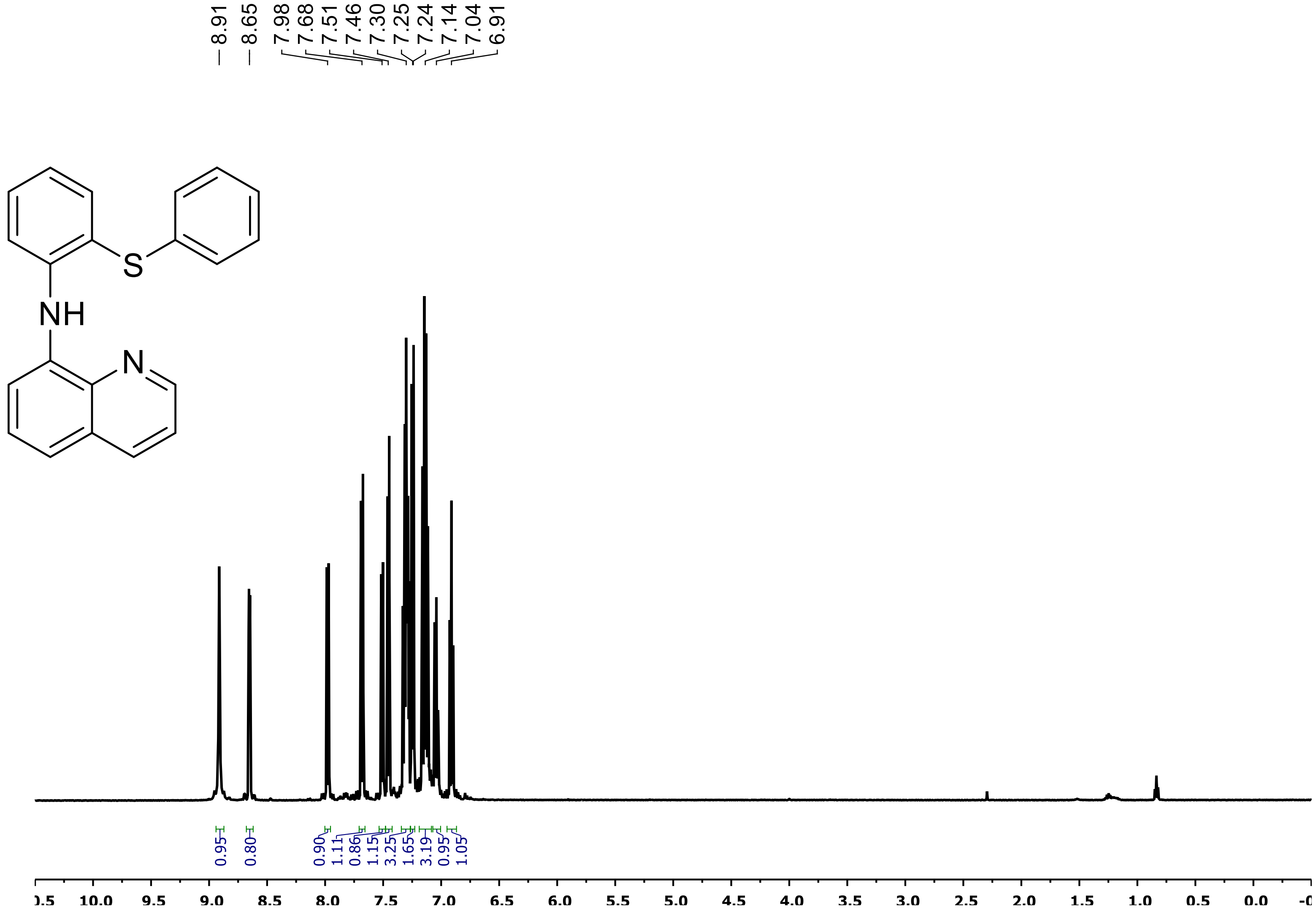

Figure S3. ^{1}H NMR (500 MHz, $CDCl_3$) spectrum of **3-H** (The peaks at 0.87 and 1.23 ppm belong to traces of pentane)

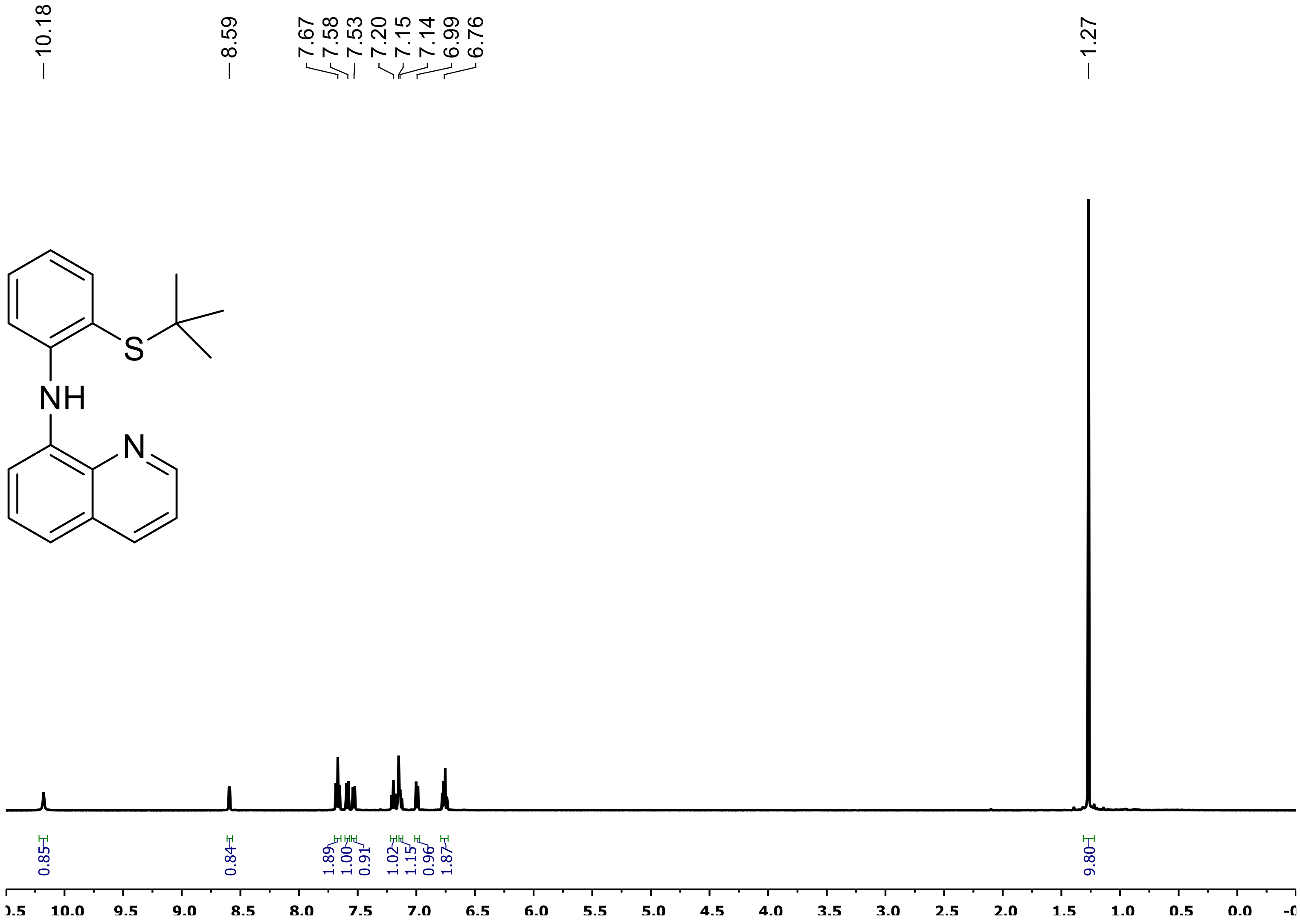

Figure S4. ^{1}H NMR (500 MHz, C_6D_6) spectrum of **4-H**

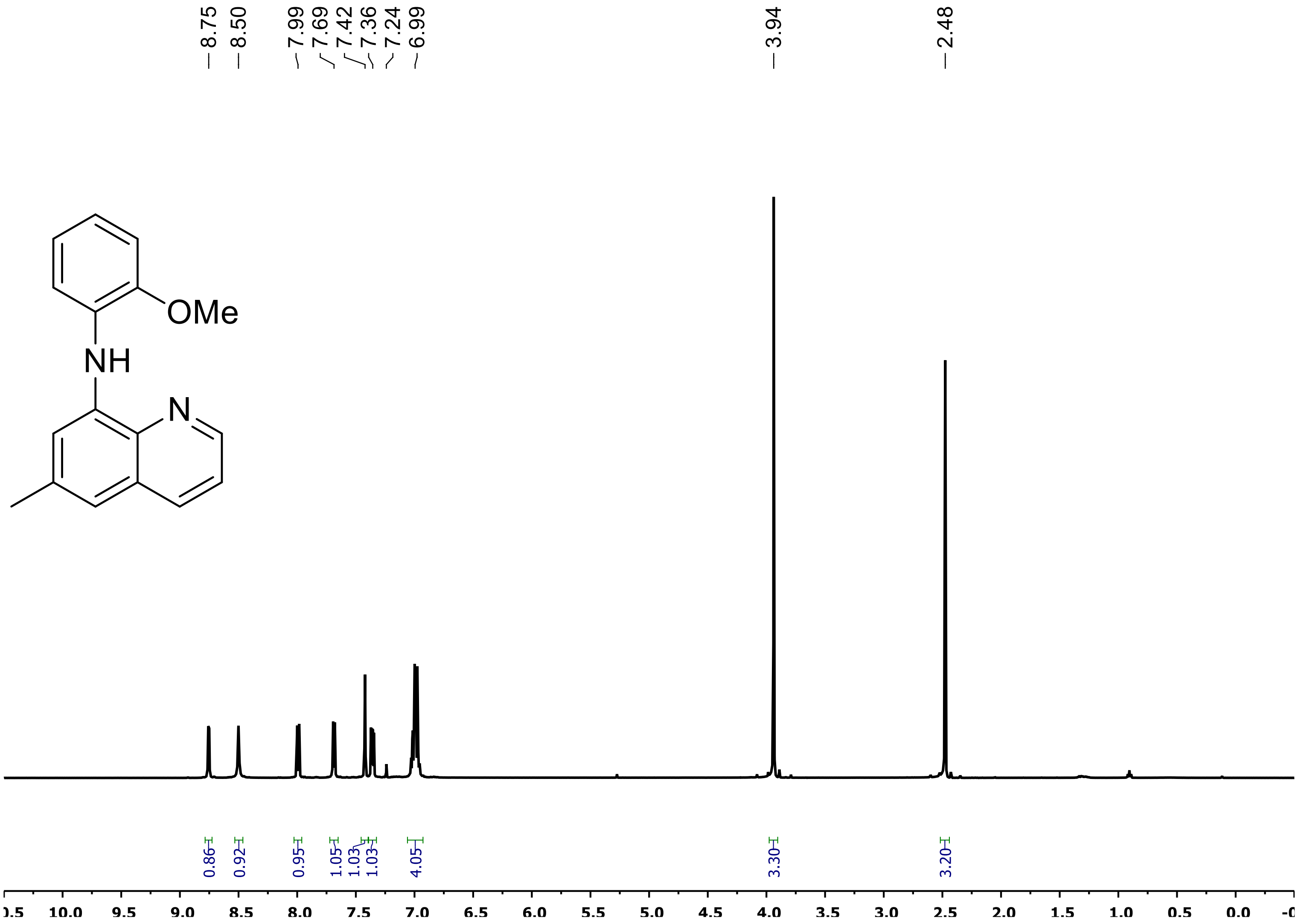

Figure S5. ^{1}H NMR (500 MHz, $CDCl_3$) spectrum of **5-H** (The peaks at 0.88 and 1.27 ppm belong to traces of pentane)

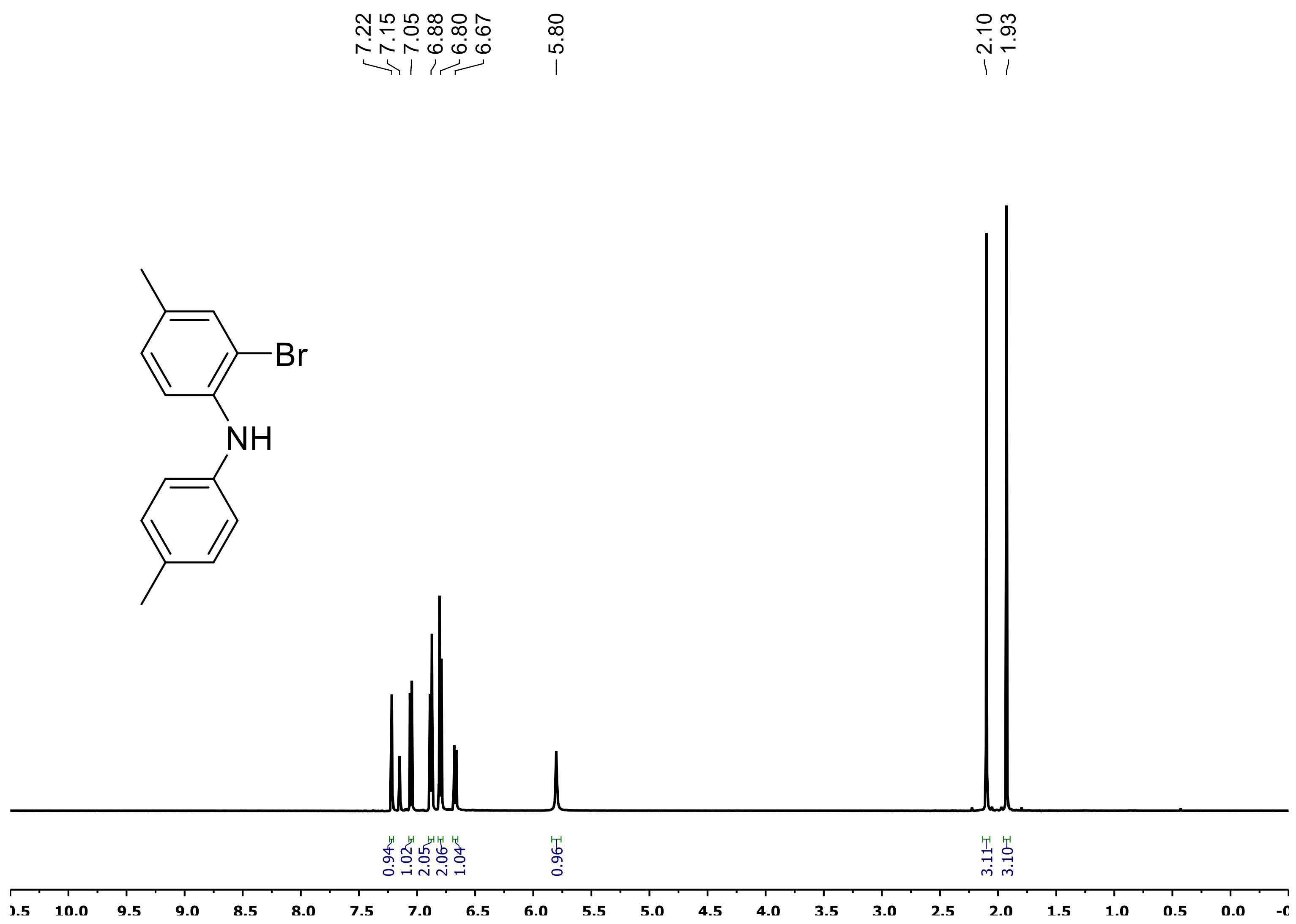

Figure S6. ^{1}H NMR (500 MHz, C_6D_6) spectrum of **S1**

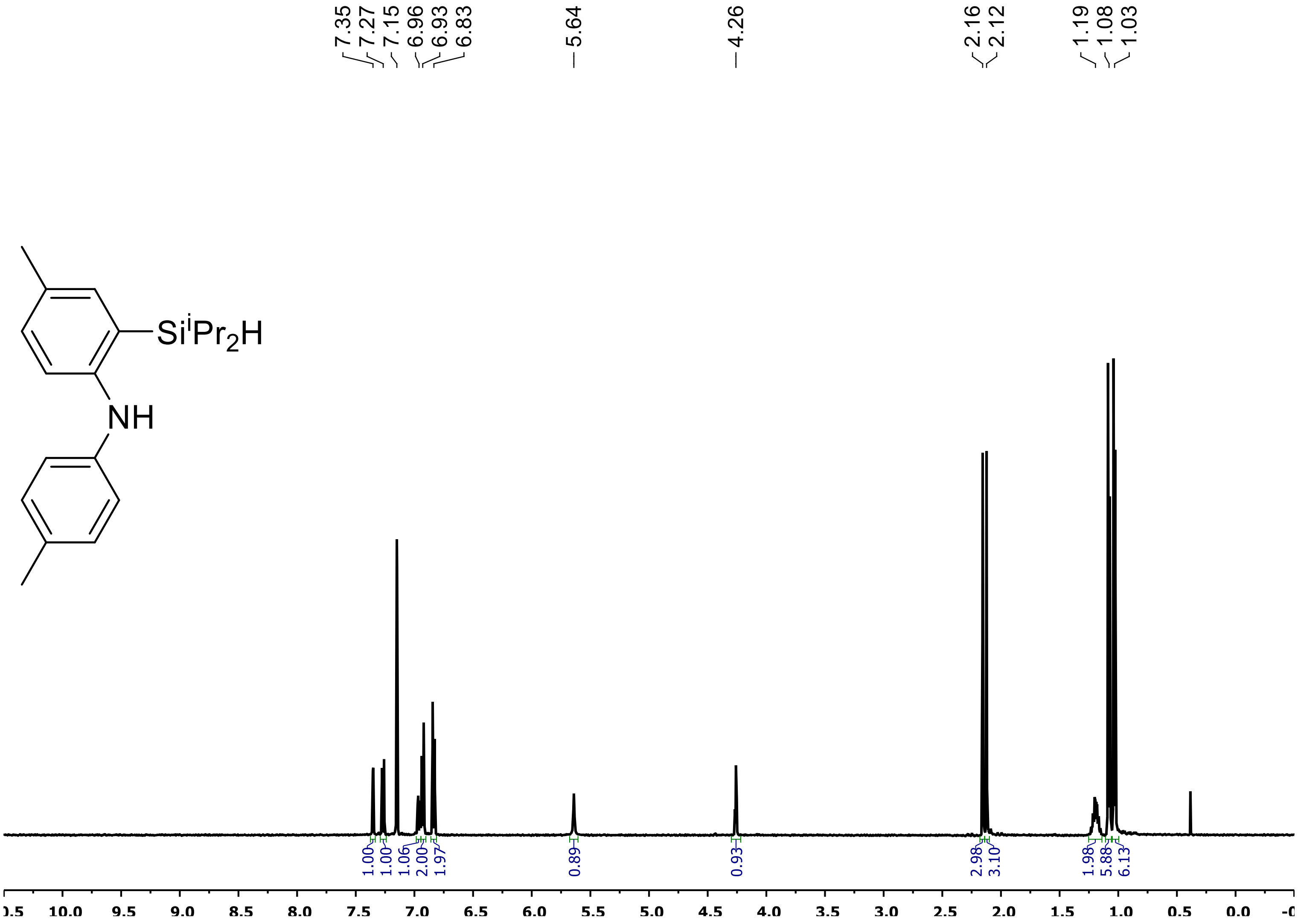

Figure S7. ^{1}H NMR (500 MHz, C_6D_6) spectrum of **8-H** (The peaks at 0.40 ppm belongs to residual water in C_6D_6)

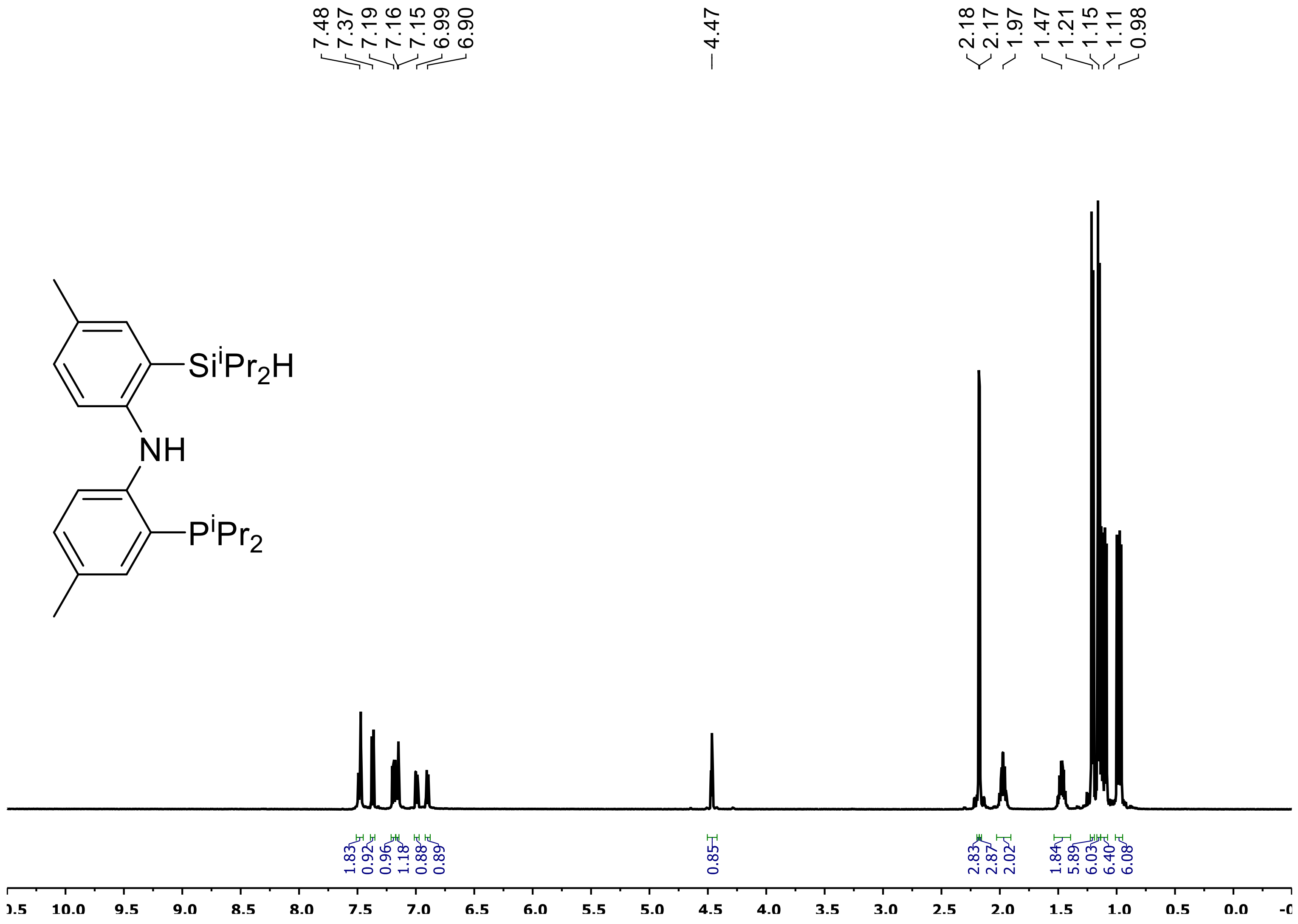

Figure S8. ^{1}H NMR (500 MHz, C_6D_6) spectrum of **9-H**

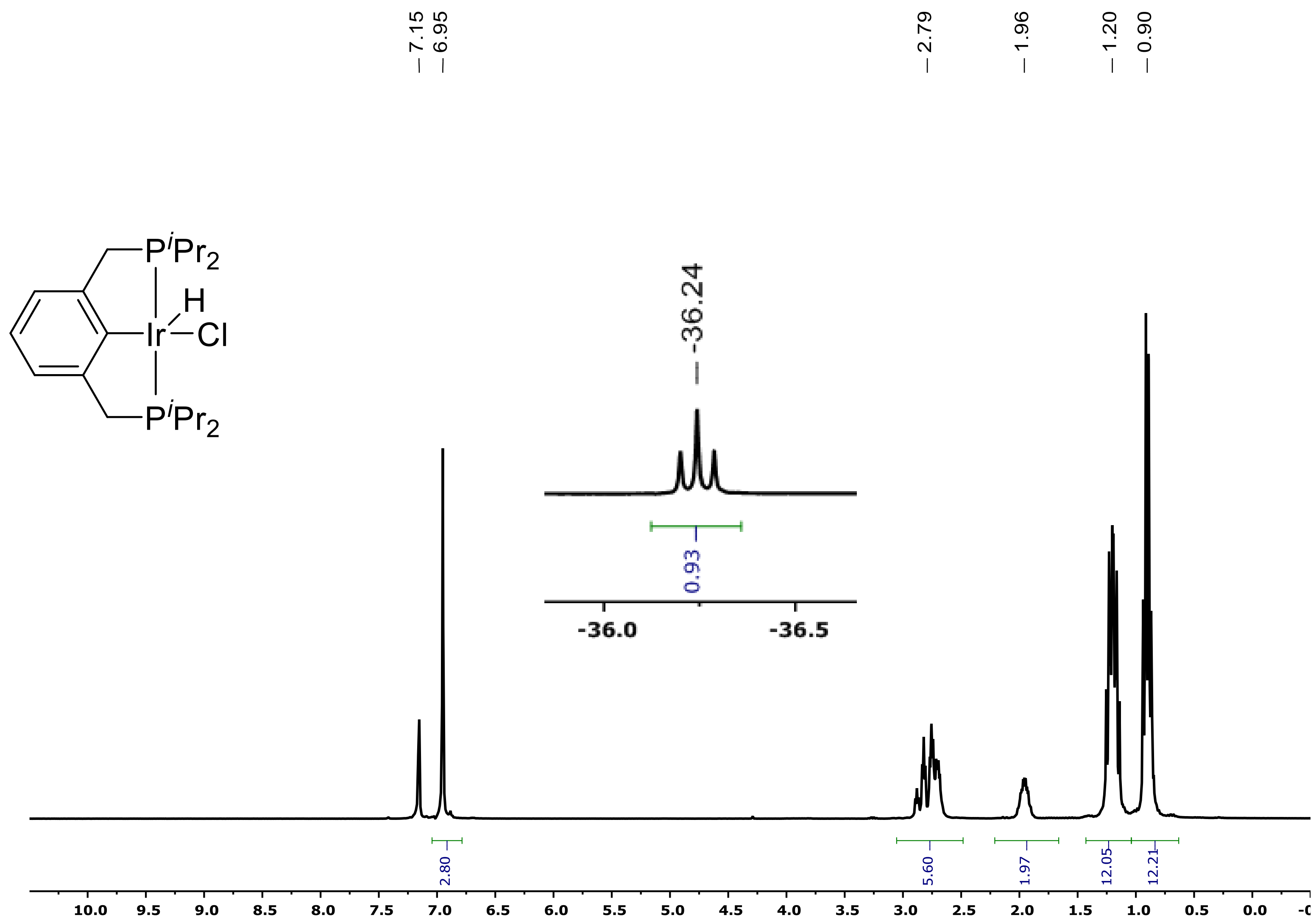

Figure S9. ^{1}H NMR (300 MHz, C_6D_6) spectrum of **13-Ir-HCl**

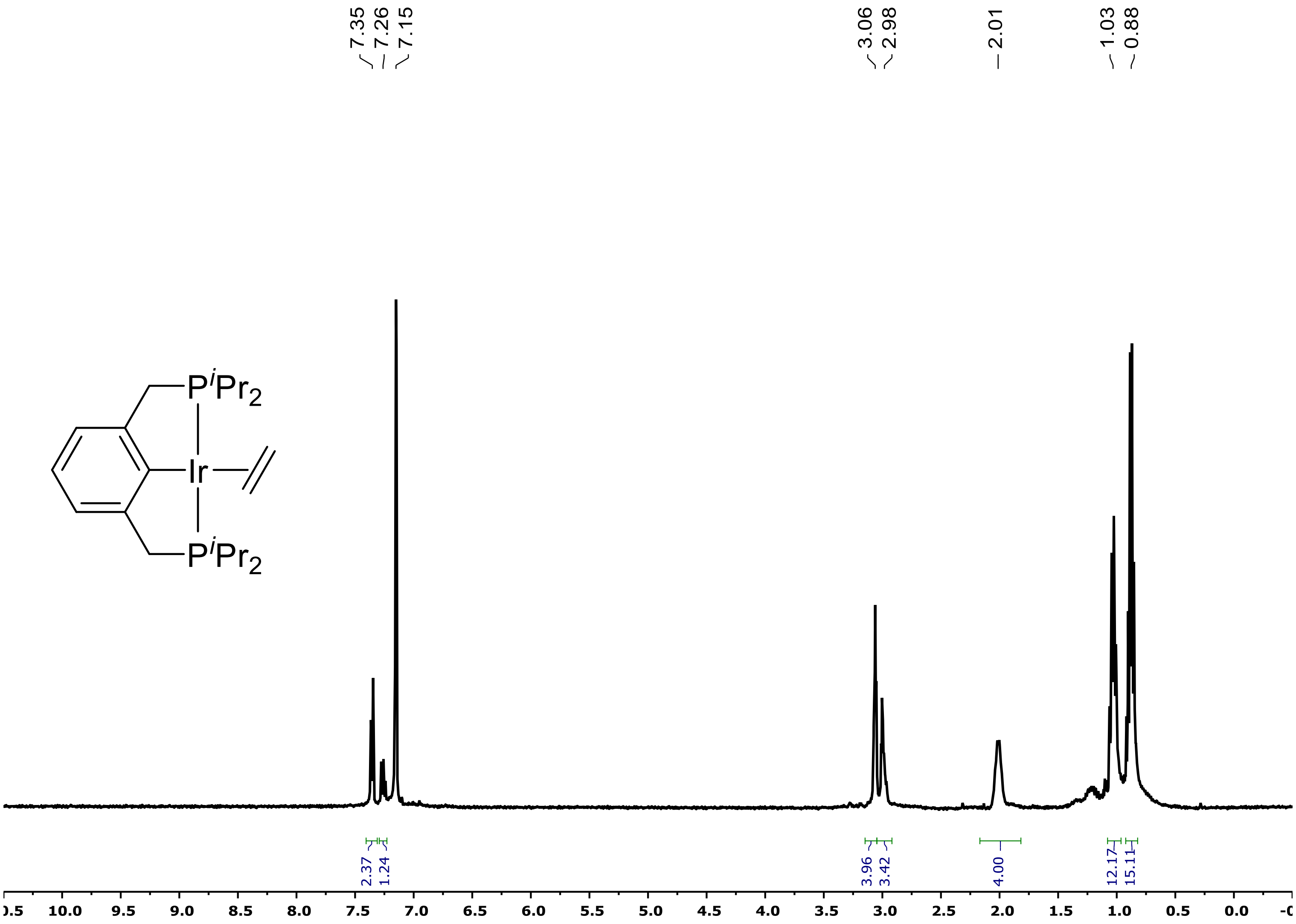

Figure S10. ^{1}H NMR (400 MHz, C_6D_6) spectrum of **13-Ir-C_2H_4** (The peaks at 0.87 and 1.23 ppm belong to traces of pentane)

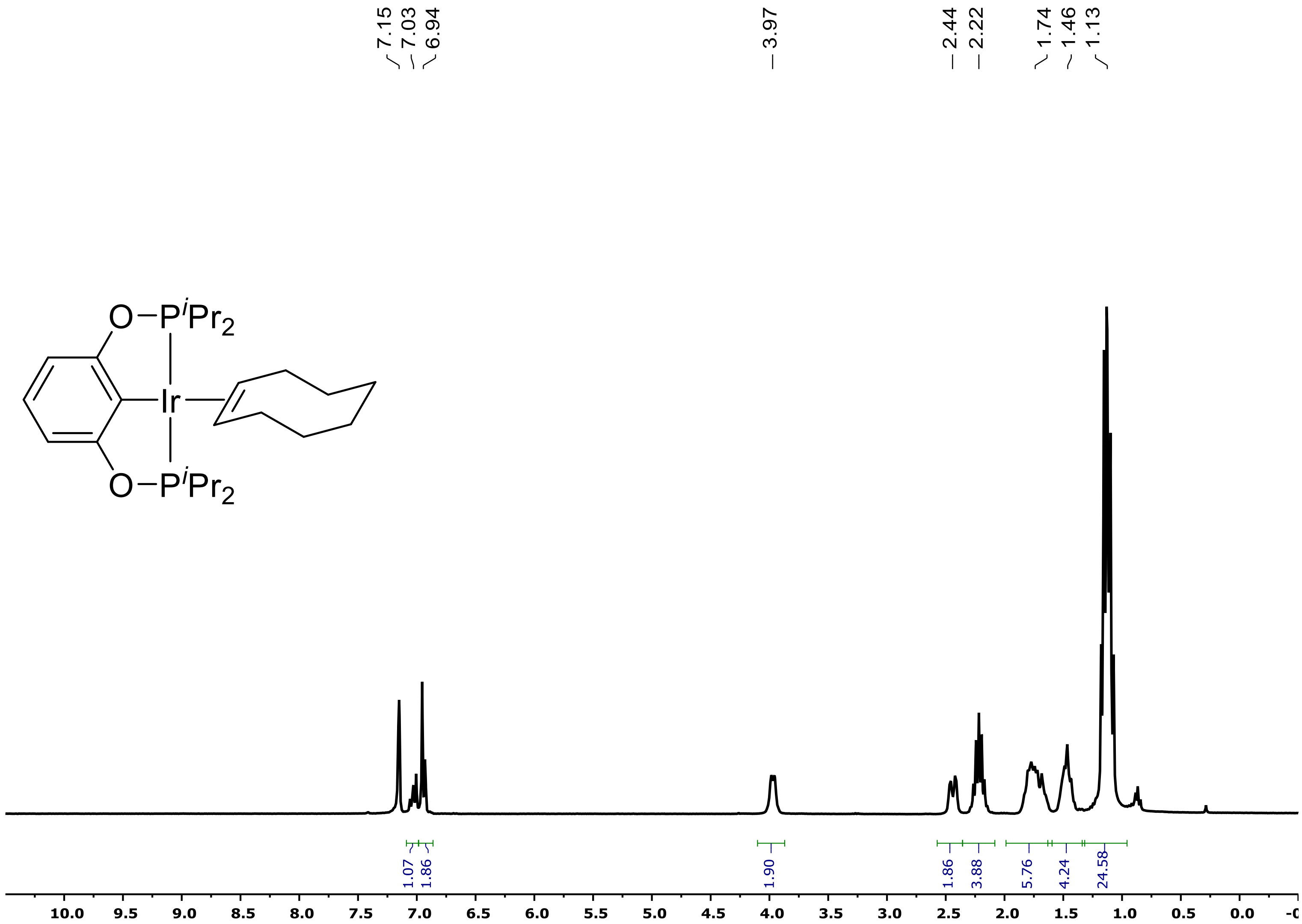

Figure S11. ^{1}H NMR (300 MHz, C_6D_6) spectrum of **14-Ir-COE** (The peaks at 0.87 ppm belong to traces of pentane)

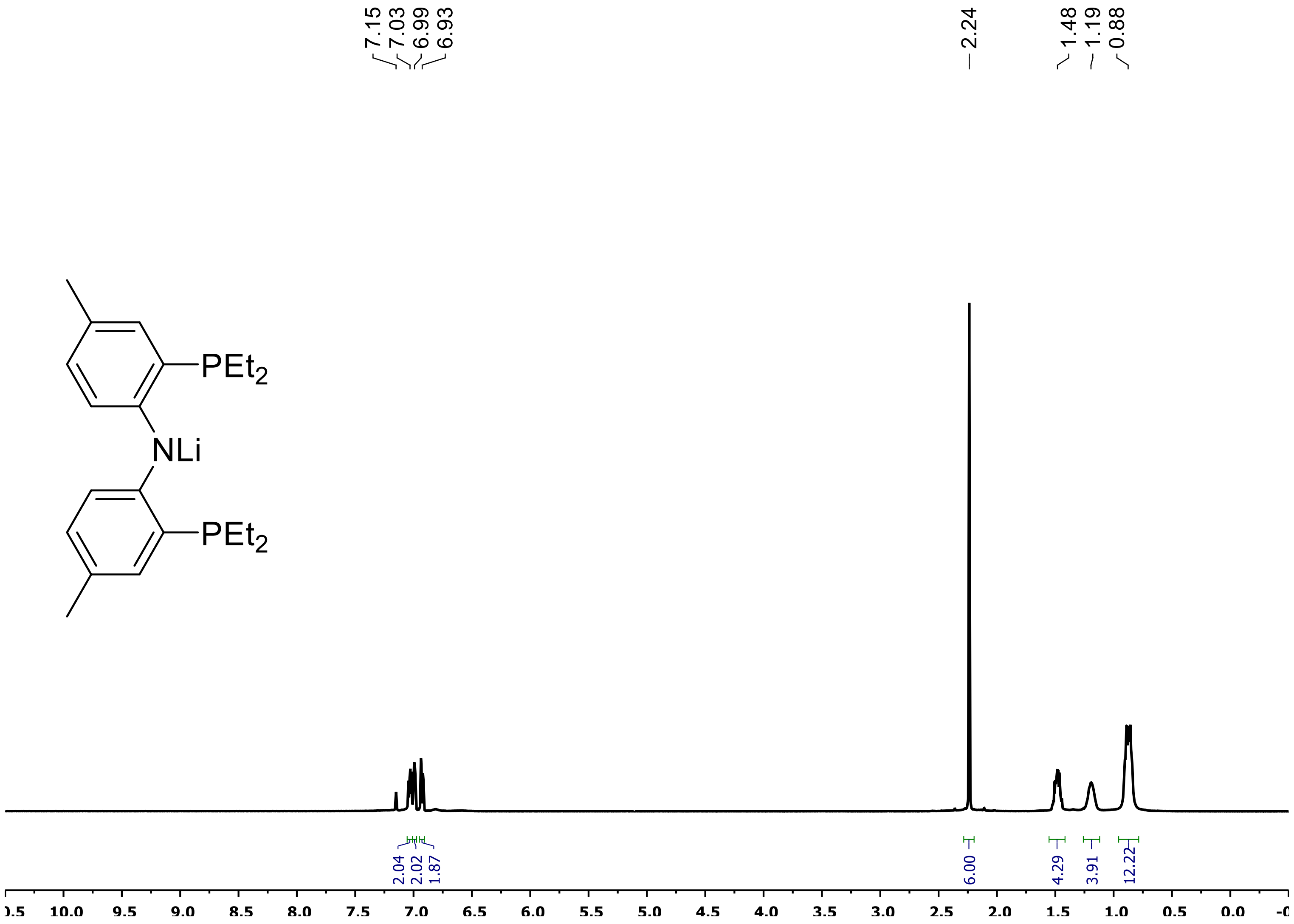

Figure S12. ^{1}H NMR (500 MHz, C_6D_6) spectrum of **15-Li**

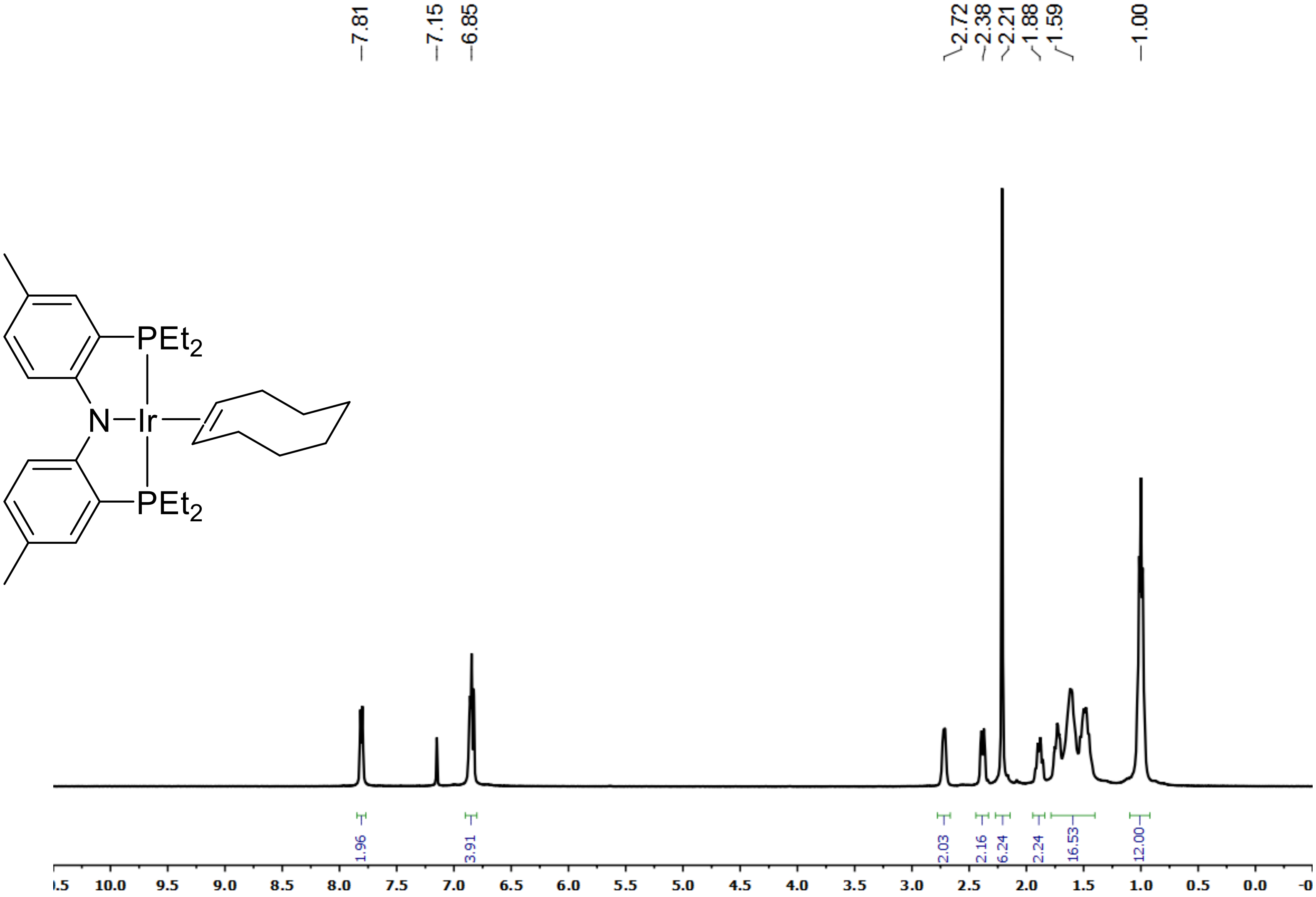

Figure S13. ^{1}H NMR (500 MHz, C_6D_6) spectrum of **15-Ir-COE**

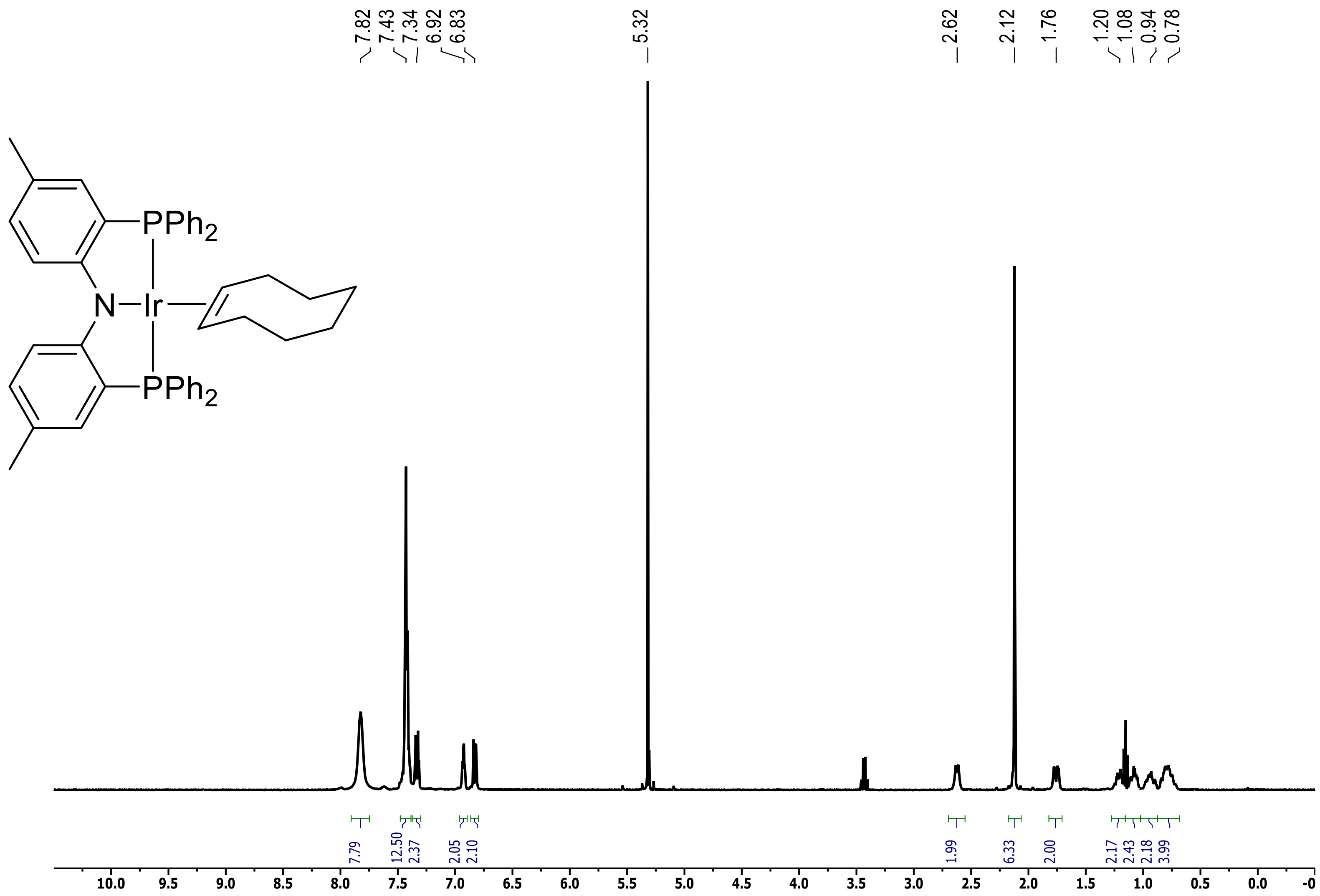

Figure S14. ^{1}H NMR (500 MHz, CD_2Cl_2) spectrum of **16-Ir-COE** (The peaks at 3.43 and 1.15 ppm belong to traces of Et_2O)

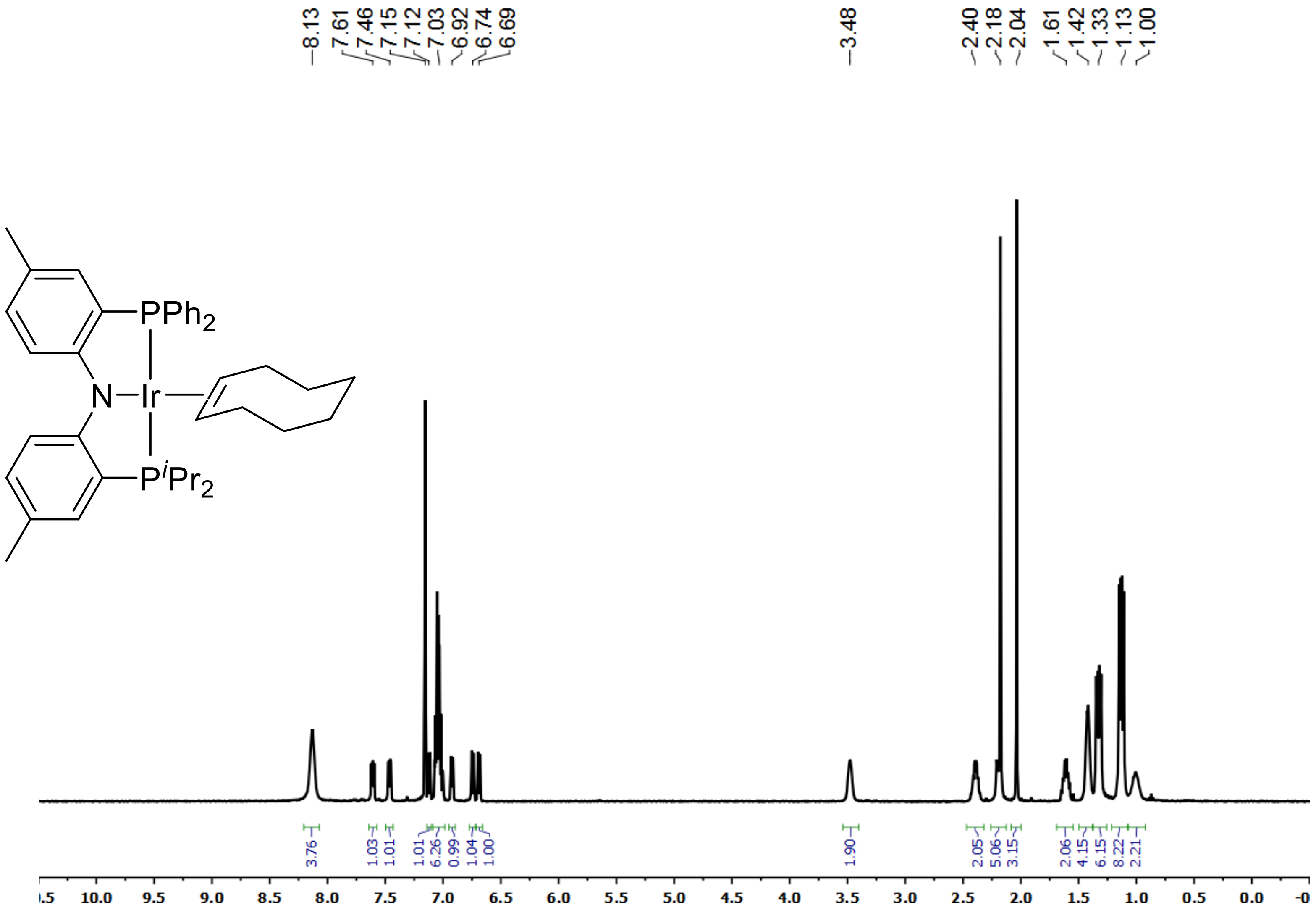

Figure S15. ¹H NMR (500 MHz, C_6D_6) spectrum of **17-Ir-COE**

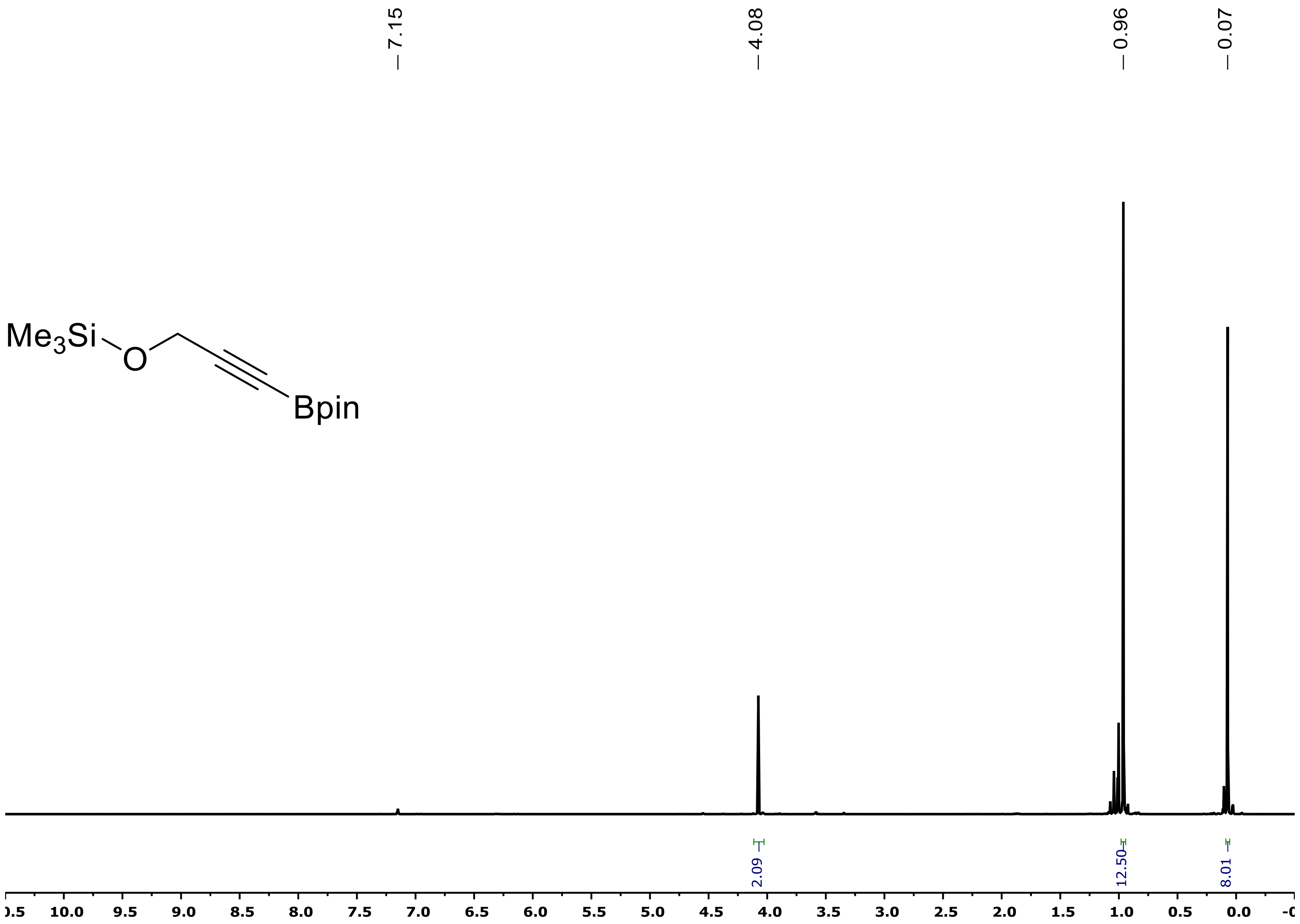

Figure S16. ^{1}H NMR (500 MHz, C_6D_6) spectrum of crude **A6-Bpin**

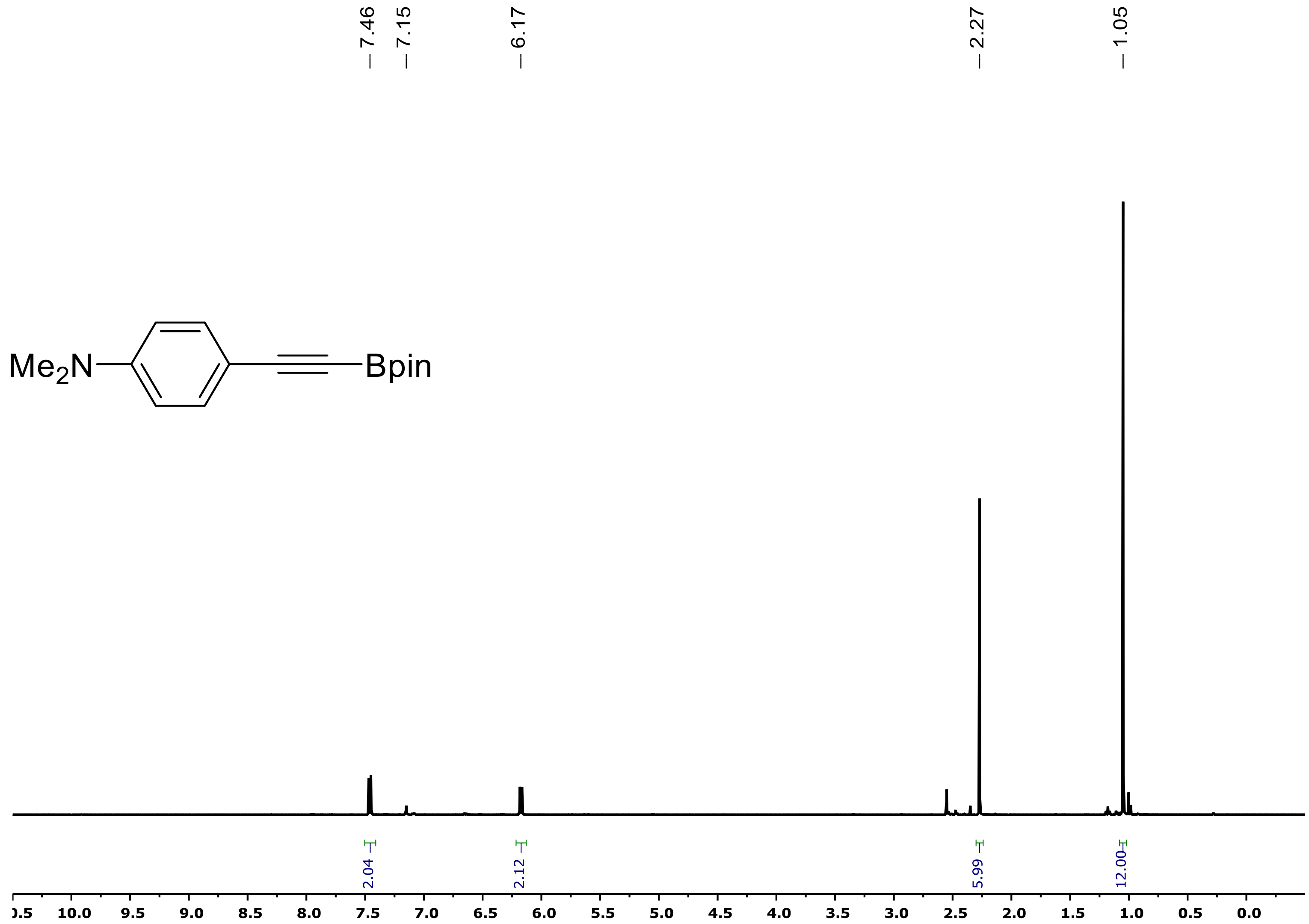

Figure S17. ^{1}H NMR (500 MHz, C_6D_6) spectrum of crude **A7-Bpin** (The peaks at 2.56, 2.52, 1.17 ppm belong to **A7-1**)

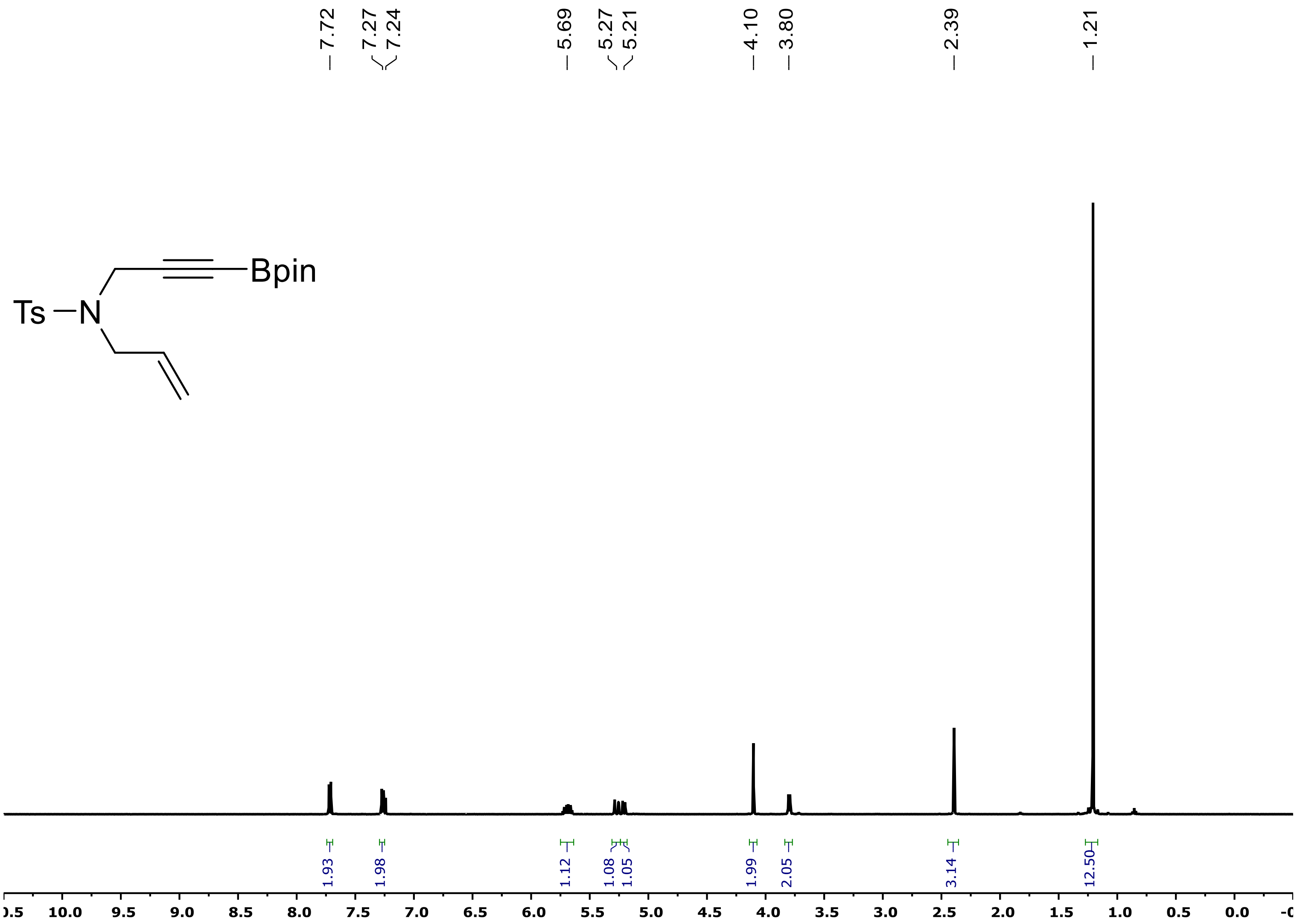

Figure S18. ^{1}H NMR (500 MHz, $CDCl_3$) spectrum of **A8-Bpin**

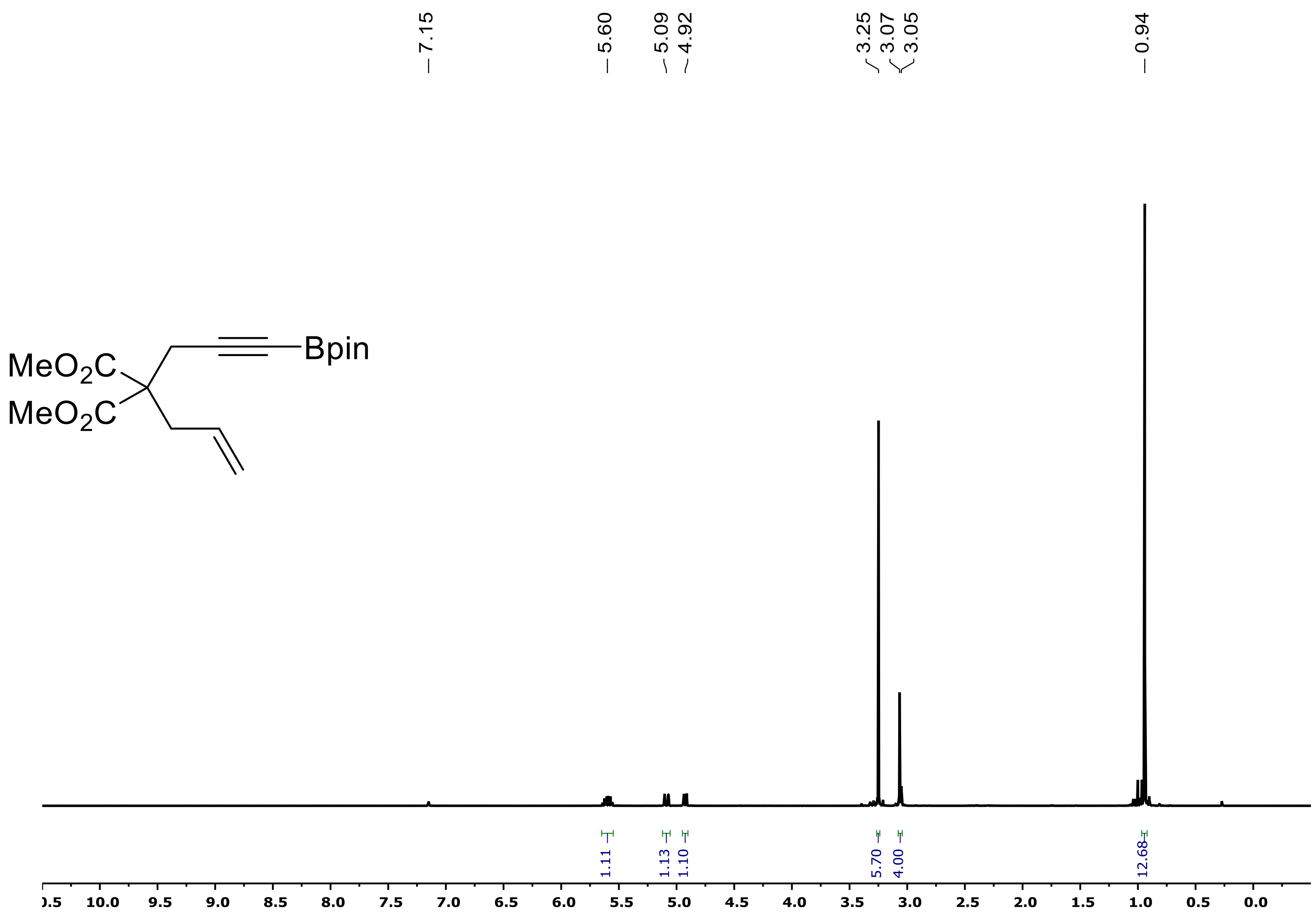

Figure S19. ^{1}H NMR (500 MHz, C_6D_6) spectrum of **A9-Bpin**

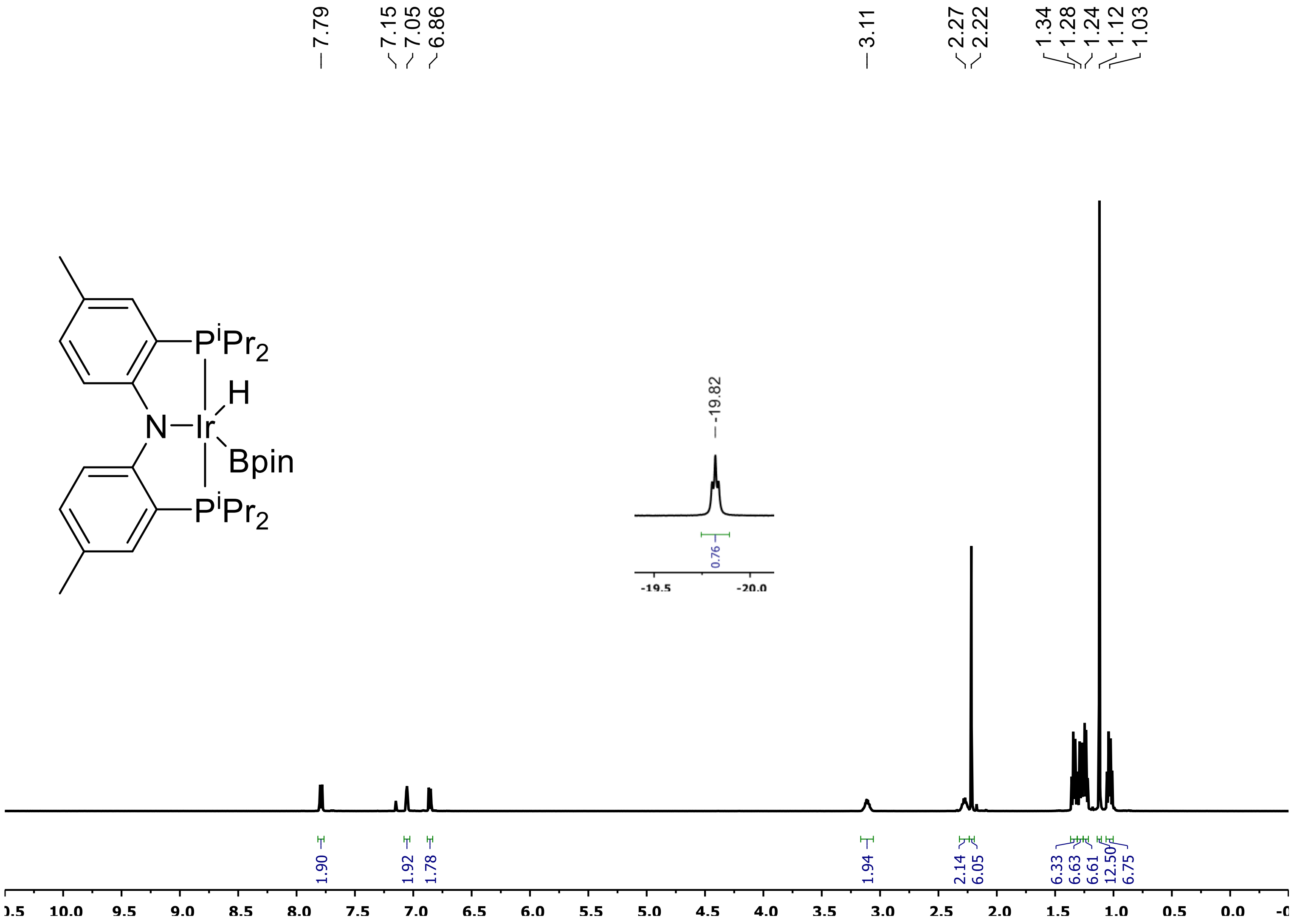

Figure S20. ^{1}H NMR (500 MHz, C_6D_6) spectrum of **10-Ir-HBpin**

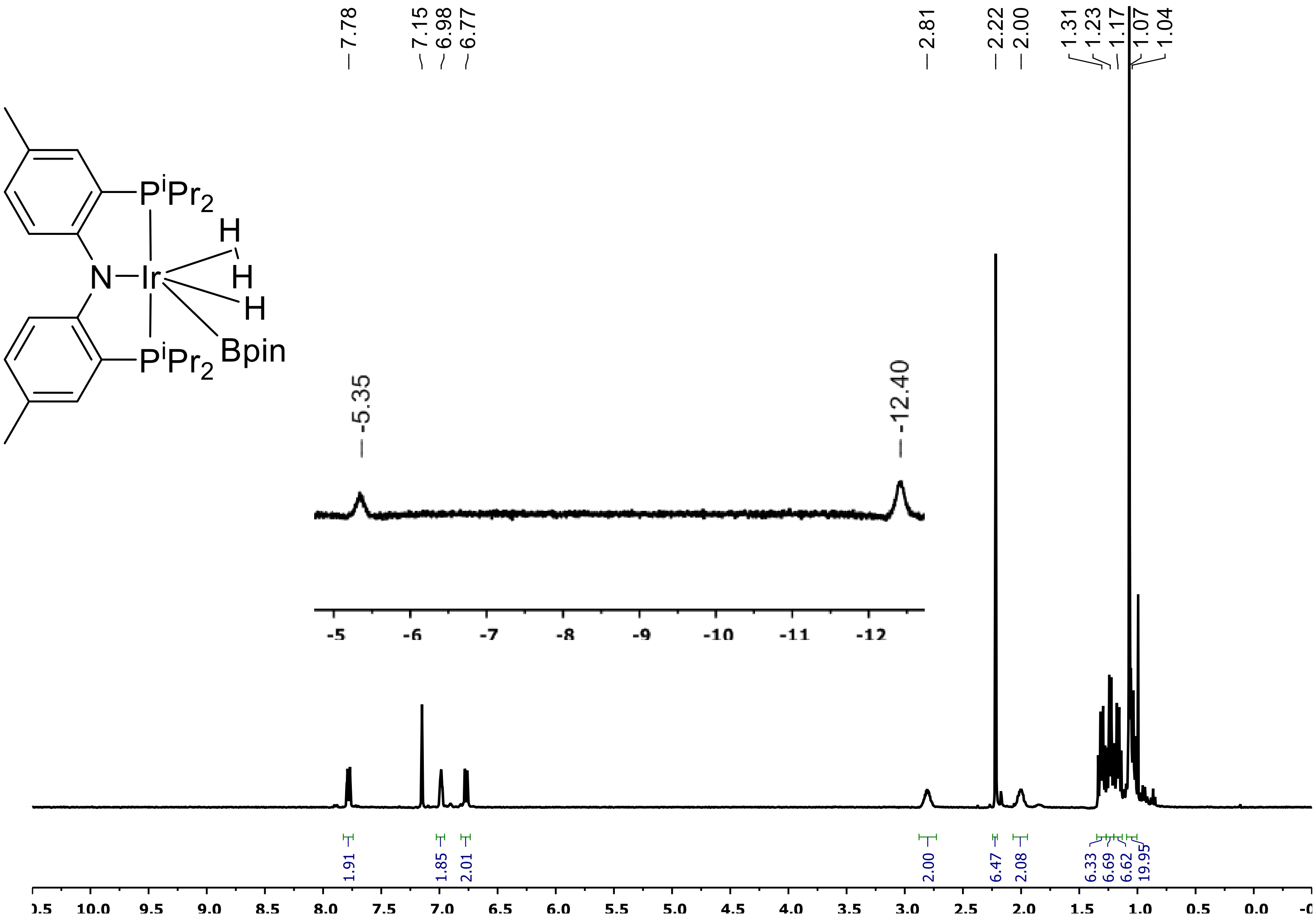

Figure S21. ^{1}H NMR (400 MHz, C_6D_6) spectrum of **10-Ir-H$_3$Bpin**

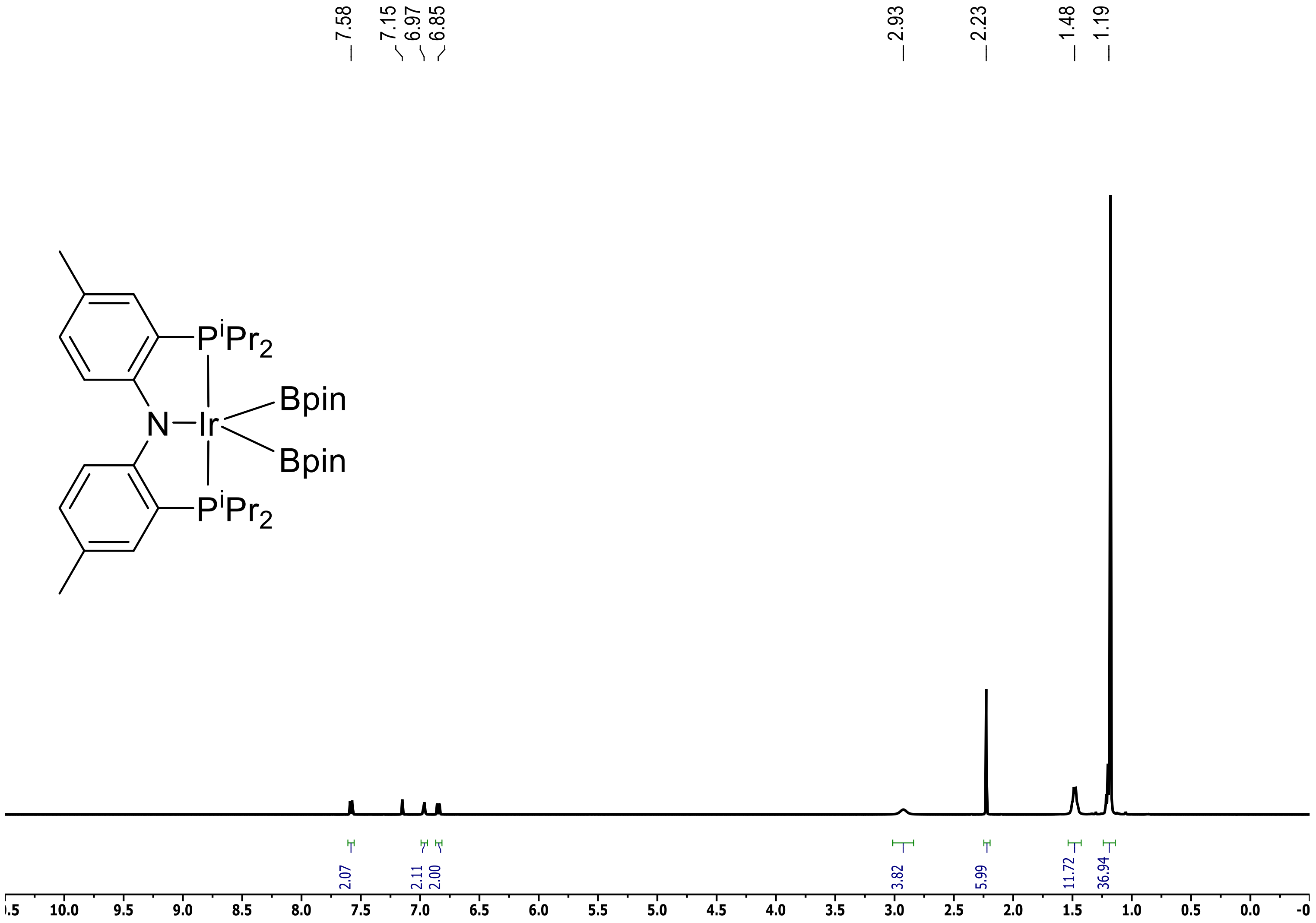

Figure S22. ^{1}H NMR (500 MHz, C_6D_6) spectrum of **10-Ir-Bpin$_2$**

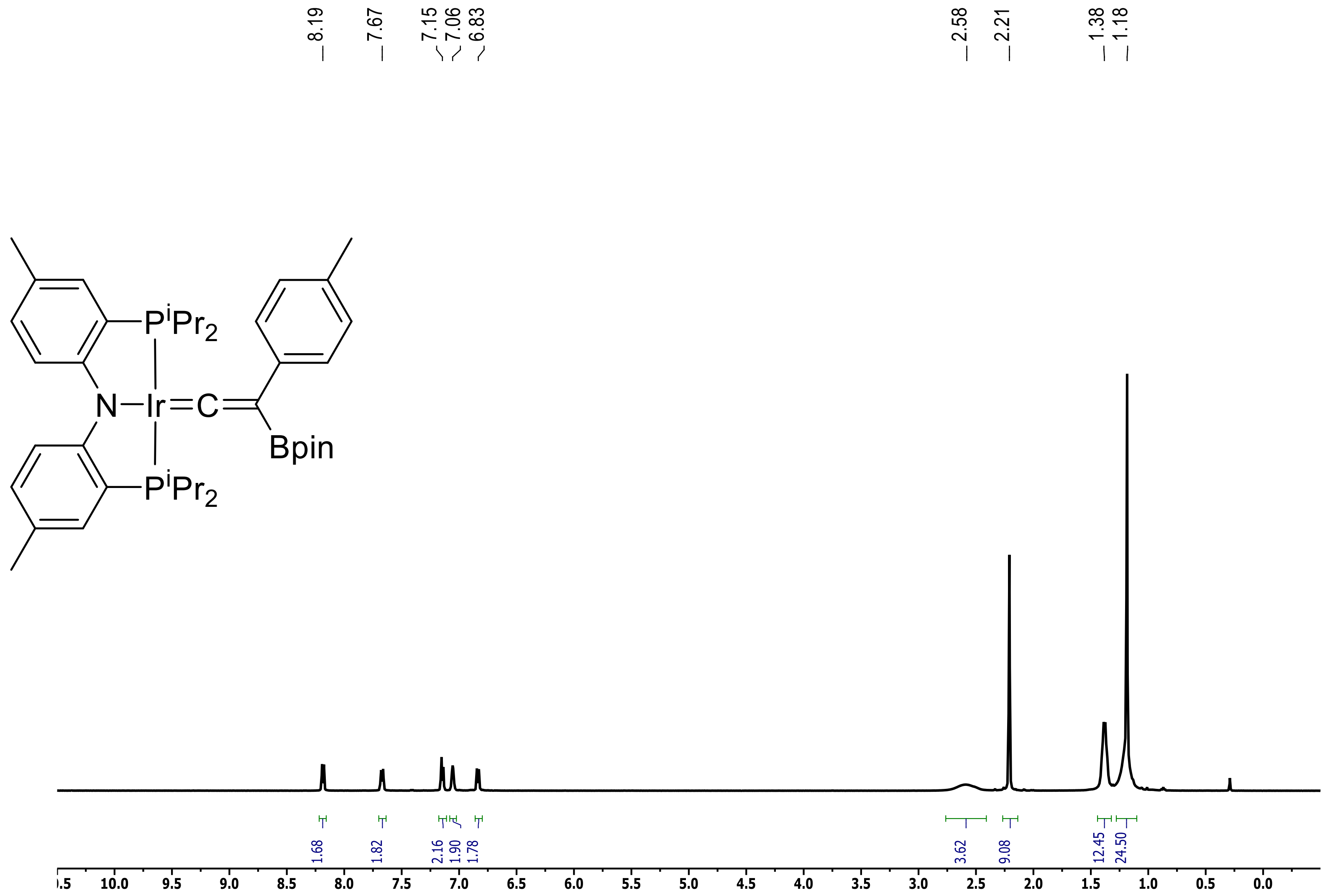

Figure S23. ^{1}H NMR (500 MHz, C_6D_6) spectrum of **10-Ir-v-tol** (The peaks at 0.28 ppm belongs to traces of silicone grease)

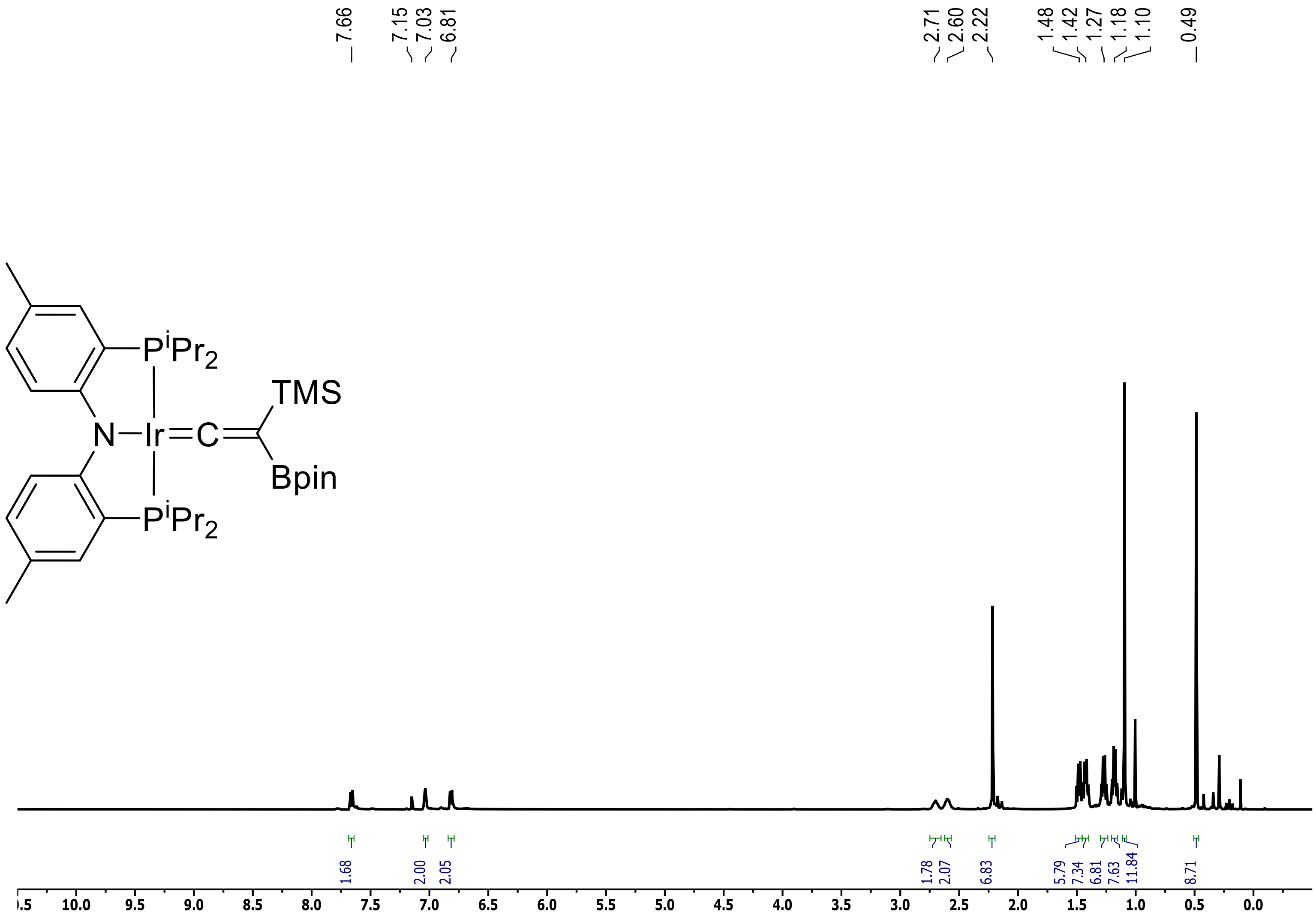

Figure S24. ^{1}H NMR (500 MHz, C_6D_6) spectrum of crude **10-Ir-v-TMS**

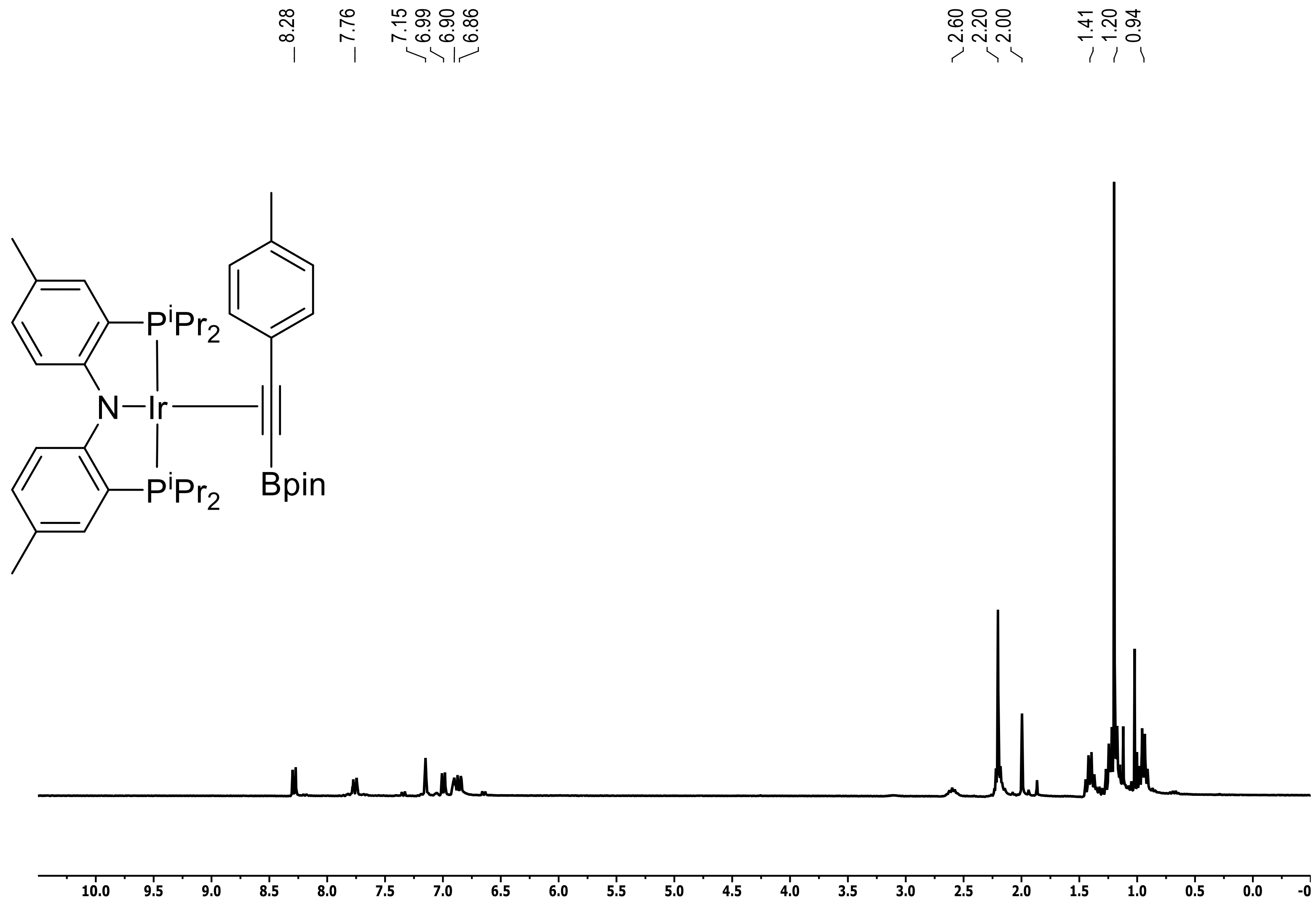

Figure S25. ^{1}H NMR (300 MHz, C_6D_6) spectrum of crude **10-Ir-p-tol**

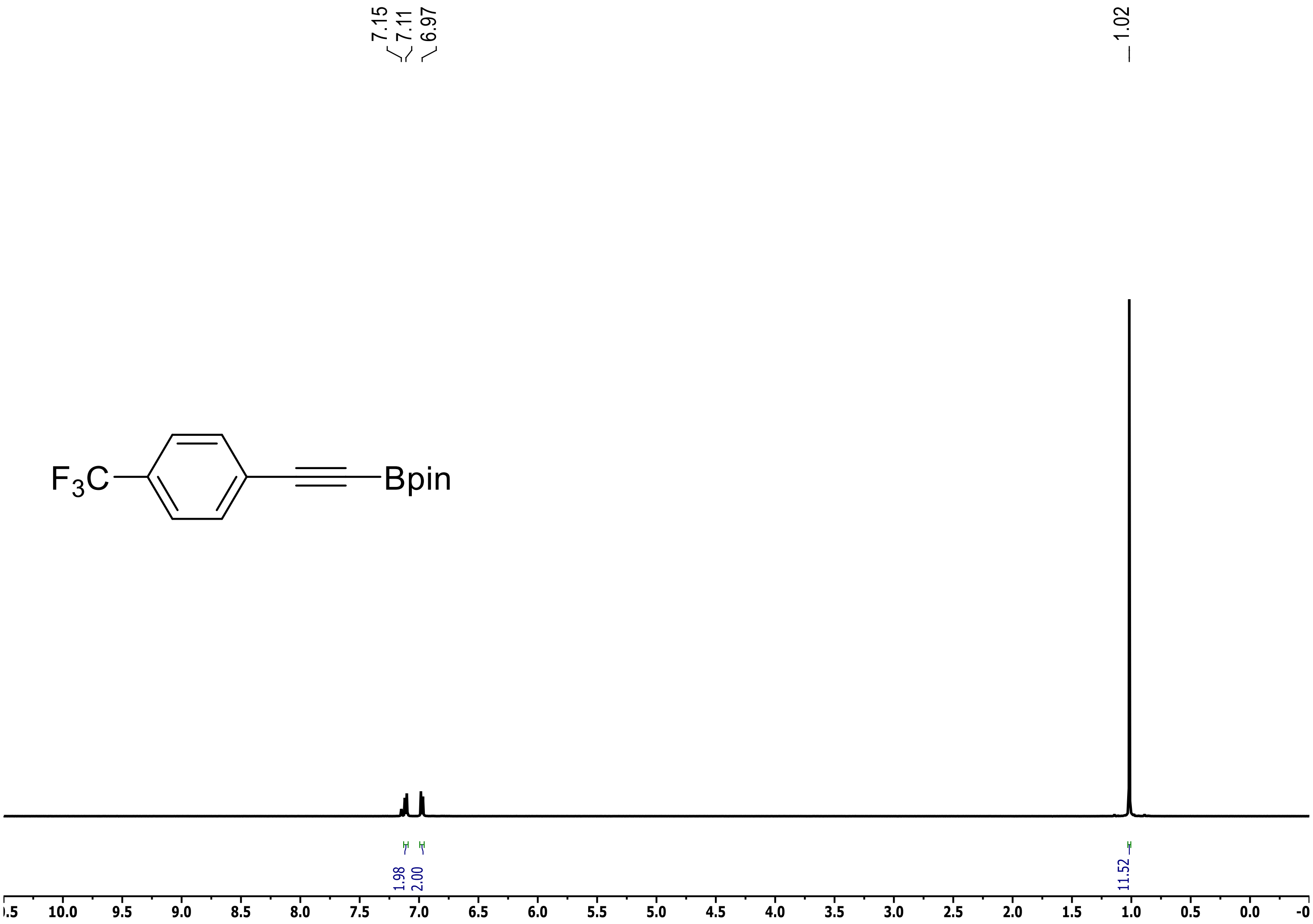

Figure S26. ^{1}H NMR (500 MHz, C_6D_6) spectrum of **A10-Bpin**

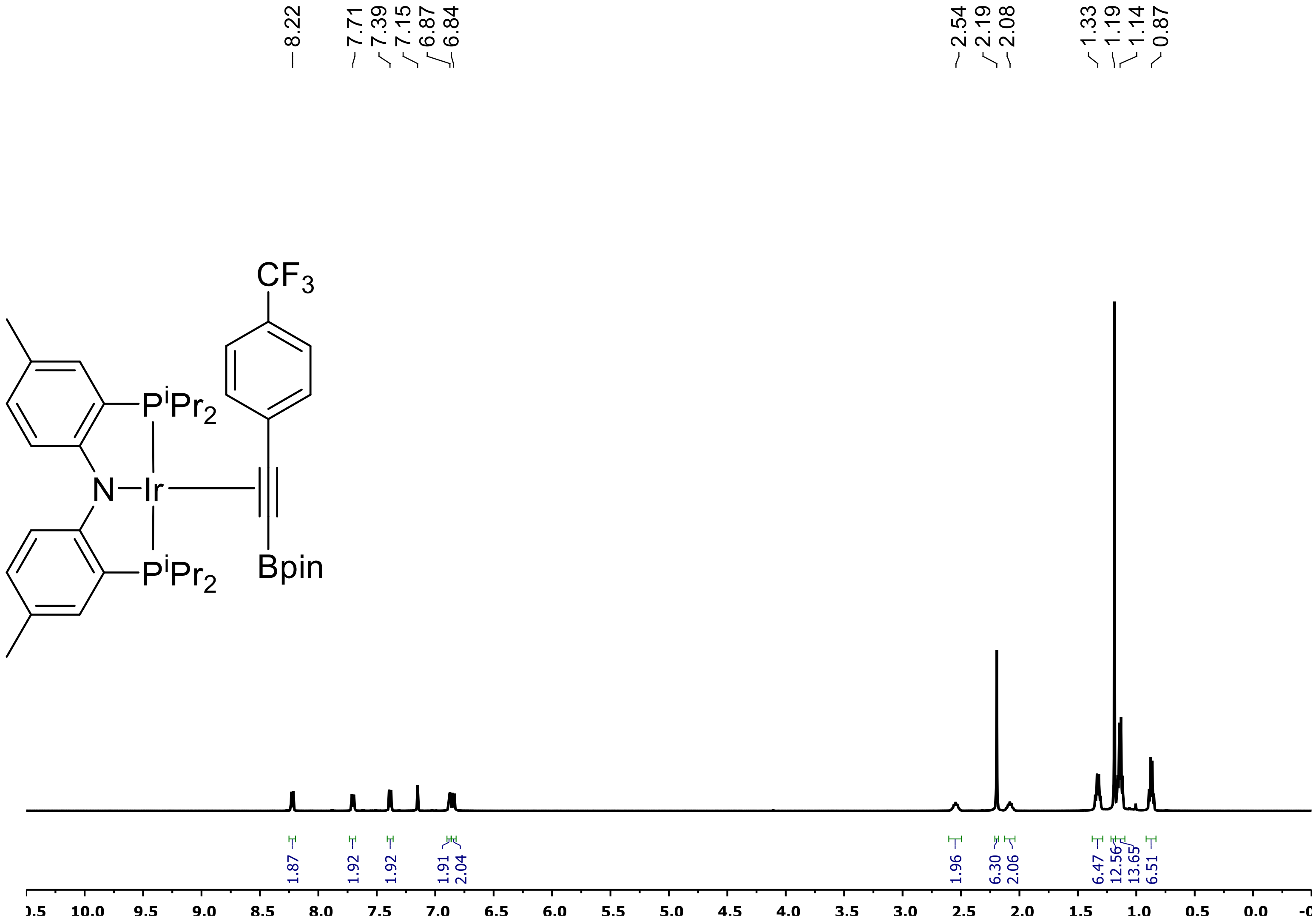

Figure S27. ^{1}H NMR (500 MHz, C_6D_6) spectrum of **10-Ir-p-F$_3$tol**

IX. SI References.

1 C.-I. Lee, J. Zhou, and O. V. Ozerov, *J. Am. Chem. Soc.*, 2013, **135**, 3560.

2 D. Liu, Y. Luo, W. Gao, and D. Cui, *Organometallics,* 2010, **29**, 1916.

3 V. V. Frolov, A. G. Mazhuga, E. K. Beloglazkina, N. V. Zyk, and M. P. Egorov, *Russ. Chem. Bull., Int. Ed.*, 2010, **59**, 544.

4 J. C. Peters, S. B. Harkins, S. D. Brown, and M. W. Day, *Inorg. Chem.*, 2001, **40**, 5083.

5 D. E. Herbert and O. V. Ozerov, *Organometallics*, 2011, **30**, 6641.

6 H. Gilman and E. A. Zuech, *J. Org. Chem.*, 1961, **26**, 3481.

7 L. Fan, S. Parkin, and O. V. Ozerov, *J. Am. Chem. Soc.*, 2005, **127**, 16772.

8 M. Gupta, C. Hagen, W. C. Kaska, R. E. Cramer, and C. M. Jensen, *J. Am. Chem. Soc.*, 1997, **119**, 840.

9 I. Göttker-Schnetmann, P. S. White, and M. Brookhart, *Organometallics,* 2004, **23**, 1766.

10 M. Montag, L. Schwartsburd, R. Cohen, G. Leitus, Y. Ben-David, J. M. L. Martin, and D. Milstein, *Angew. Chem., Int. Ed.*, 2007, **46**, 1901.

11 D. Morales-Morales, R. Redón, C. Yung, and C. M. Jensen, *Inorg. Chim. Acta*, 2004, **357**, 2953.

12 L. P Press and O. V. Ozerov, *manuscript in preparation*.

13 J. J. Davidson, J. C. DeMott, C. Douvris, N. Bhuvanesh, C.-H. Chen, D. E. Herbert, C.-I. Lee, B. J. McCulloch, B. M. Foxman, and O. V. Ozerov, *Inorg. Chem.*, 2015, **54**, 2916.

14 K. T. Sylvester, and P. J. Chirik, *J. Am. Chem. Soc.*, 2009, **131**, 8772.

15 R. Balamurugan, R. B. Kothapalli, and G. K. Thota, *Eur. J. Org. Chem.* 2011, 1557.

16 M. J. Frisch, G. W. Trucks, H. B. Schlegel, G. E. Scuseria, M. A. Robb, J. R. Cheeseman, G. Scalmani, V. Barone, B. Mennucci, G. A. Petersson, H. Nakatsuji, M. Caricato, X. Li, H. P.

Hratchian, A. F. Izmaylov, J. Bloino, G. Zheng, J. L. Sonnenberg, M. Hada, M. Ehara, K. Toyota, R. Fukuda, J. Hasegawa, M. Ishida, T. Nakajima, Y. Honda, O. Kitao, H. Nakai, T. Vreven, J. A. Montgomery, Jr., J. E. Peralta, F. Ogliaro, M. Bearpark, J. J. Heyd, E. Brothers, K. N. Kudin, V. N. Staroverov, R. Kobayashi, J. Normand, K. Raghavachari, A. Rendell, J. C. Burant, S. S. Iyengar, J. Tomasi, M. Cossi, N. Rega, J. M. Millam, M. Klene, J. E. Knox, J. B. Cross, V. Bakken, C. Adamo, J. Jaramillo, R. Gomperts, R. E. Stratmann, O. Yazyev, A. J. Austin, R. Cammi, C. Pomelli, J. W. Ochterski, R. L. Martin, K. Morokuma, V. G. Zakrzewski, G. A. Voth, P. Salvador, J. J. Dannenberg, S. Dapprich, A. D. Daniels, Ö. Farkas, J. B. Foresman, J. V. Ortiz, J. Cioslowski, and D. J. Fox, Gaussian, Inc., Wallingford CT, 2009.

17 Y. Zhao and D. Truhlar, *Theor. Chem. Acc.*, 2008, **120**, 215.

18 FRAMBO v. 4.1.05 "Program for Data Collection on Area Detectors" BRUKER-Nonius Inc., 5465 East Cheryl Parkway, Madison, WI 53711-5373 USA.

19 G. M. Sheldrick, "Cell_Now (version 2008/1): Program for Obtaining Unit Cell Constants from Single Crystal Data": University of Göttingen, Germany, 2008.

20 APEX2 "Program for Data Collection and Integration on Area Detectors" BRUKER AXS Inc., 5465 East Cheryl Parkway, Madison, WI 53711-5373 USA.

21 G. M. Sheldrick, "SADABS (version 2008/1): Program for Absorption Correction for Data from Area Detector Frames", University of Göttingen, Germany, 2008.

22 G. M. Sheldrick, *Acta Cryst.*, 2008, **A64**, 112.

23 O. V. Dolomanov, L. J. Bourhis, R. J. Gildea, J. A. K. Howard, and H. Puschmann, *J. Appl. Cryst.*, 2009, **42**, 339.

24 A. L. Spek, *J. Appl. Cryst.*, 2003, **36**, 7; A. L. Spek, Utrecht University, Utrecht, The Netherlands, 2008.

25 Our efforts to restrain the thermal parameters resulted in high correlation given the close proximity of the disordered group; subsequently EADP was used to keep the thermal ellipsoids of these disordered atoms meaningful.

26 Higher reliability factors R1 and wR2 were a result of poor quality crystals.

27 E. Calimano and T. D. Tilley, *Dalton Trans.*, 2010, **39**, 9250.
